# Ring-closing-metathesis-based synthesis of annellated coumarins from 8-allylcoumarins

**DOI:** 10.3762/bjoc.14.278

**Published:** 2018-12-05

**Authors:** Christiane Schultze, Bernd Schmidt

**Affiliations:** 1 Universität Potsdam, Institut fuer Chemie, Karl-Liebknecht-Straße 24–25, D-14476 Potsdam-Golm, Germany

**Keywords:** coumarins, heterocycles, isomerization, olefin metathesis, ruthenium

## Abstract

8-Allylcoumarins are conveniently accessible through a microwave-promoted tandem Claisen rearrangement/Wittig olefination/cyclization sequence. They serve as a versatile platform for the annellation of five- to seven-membered rings using ring-closing olefin metathesis (RCM). Furano-, pyrano-, oxepino- and azepinocoumarins were synthesized from the same set of precursors using Ru-catalyzed double bond isomerizations and RCM in a defined order. One class of products, pyrano[2,3-*f*]chromene-2,8-diones, were inaccessible through direct RCM of an acrylate, but became available from the analogous allyl ether via an assisted tandem catalytic RCM/allylic oxidation sequence.

## Introduction

**Figure 1:**
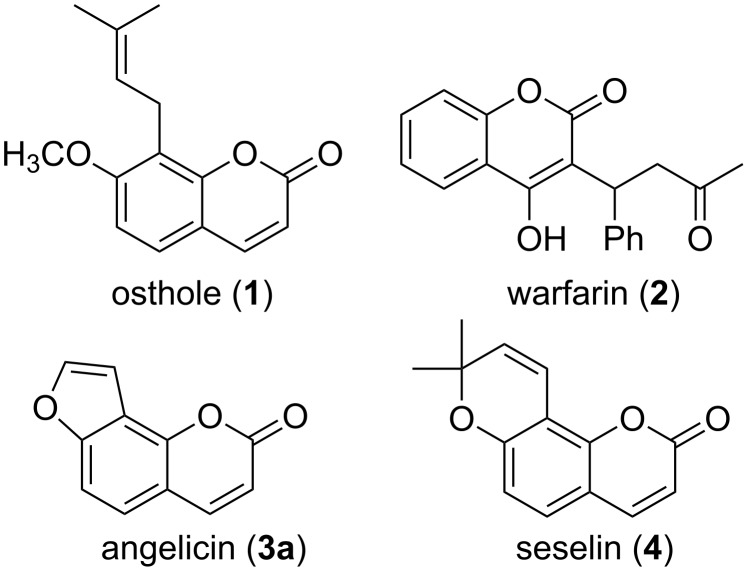
Illustration of coumarin taxonomy.

Naturally occurring coumarins and synthetic derivatives have attracted considerable attention, because many of these compounds are pharmacologically active [1–4]. Their activity profiles are quite diverse and range from anticoagulant via anti-infective, anticancer to antineurodegenerative activities [2–3]. The majority of natural coumarins are secondary metabolites isolated from plants [5–7]. A commonly used taxonomy for these natural products (which has been extended to the non-natural analogues) is based on the coumarin structure (Figure 1) [4,8].

It distinguishes between simple coumarins with substituents only at the benzene part (e.g., osthole, a natural product with Ca^2+^-channel antagonist activity) [9], coumarins with substituents at the pyrone part (e.g., warfarin, a synthetic clinically used anticoagulant) [10], and heteroannellated coumarins, in which a heterocycle is annellated to the benzene ring of the coumarin skeleton. In particular the latter group is often further divided into sections according to ring size (five-membered rings: furanocoumarins; six-membered rings: pyranocoumarins) and location of the annellated ring (linear vs angular). Angelicin (**3a**, also named isopsoralen), for instance, is an angular furanocoumarin from *Psoralea corylifolia* [11–12] that is moderately cytotoxic [13] and exhibits anti-oxidative activity [14], but is significantly less phototoxic than the linear isomer psoralen, due to its inability to cross link DNA [15]. This consideration has, for example, led to the development of substituted angelicins rather than psoralens as potential anti-influenza drugs [16]. Seselin (**4**) is an example of an angular pyranocoumarin found in various plants, e.g., from the family of *Rutaceae* [17]. Among other bioactivities, the compound itself and some natural and non-natural derivatives induce apoptosis in melanoma HTB-140 cells [18].

Synthetic approaches to substituted coumarins in general and heteroannellated coumarins [19] in particular can start from other naturally occurring coumarins [20] or may involve the construction of the coumarin skeleton. For the latter group of syntheses several classical methods, such as the Perkin condensation, are available, which have been covered in earlier reviews [5–6,8]. Unfavorable reaction conditions, low yields and a sometimes limited scope make the development of alternatives to these established methods necessary. Examples from the past 15 years include transition metal-catalyzed transformations [21–23], solid-phase synthesis directed at combinatorial library design [24] and organocatalytic annellation reactions [25–26].

Sparked by our interest in the development and application of sequential one-pot transformations and motivated by the relevance of prenylated and other substituted coumarins in natural products and medicinal chemistry, we [27–29] and others [30] have investigated a microwave-promoted tandem reaction for the synthesis of 8-substituted coumarins over the past few years. Starting materials are allyl ethers of salicylic aldehydes or ketones **5** and the stable ylide ethyl (triphosphoranylidene)acetate (**6**), which upon microwave irradiation undergo a tandem Claisen rearrangement/Wittig olefination/cyclization sequence. This sequence was pioneered by the groups of Harwood [31–32] and Mali [33–36], and its Wittig olefination/cyclization part has been employed in the synthesis of various coumarins without alkyl substituents at position 8 [37–39]. In all previous reports conventional heating was used to induce the tandem sequence.

In this contribution we report how 8-allylcoumarins obtained through the microwave-promoted tandem sequence can be elaborated into heteroannellated coumarins that are either natural products or close ring-expanded analogues, using ring-closing olefin metathesis (RCM) reactions. Precedence for the use of RCM [40] in the synthesis and functionalization of coumarins is scarce, considering the vast number of applications olefin metathesis has found [41] and taking into account the high relevance of coumarins. Construction of the coumarin by RCM has been reported by few groups [42–45] and heteroannellations to the coumarin scaffold based on RCM are also limited in number and have mostly not been surveyed systematically [46–53].

## Results and Discussion

To study the heteroannellation reactions, a set of four 8-allyl-7-hydroxycoumarins **8** were synthesized starting from the MOM-protected precursors **5a**–**d** using the conditions of the microwave-promoted tandem sequence [29]. The intermediate MOM-protected coumarins were not isolated but immediately deprotected by treatment with aq HCl in methanol. Isolation of the MOM-protected coumarins **7** [29] and deprotection in a separate step resulted in virtually identical overall yields of coumarins **8** and did therefore not offer any advantage (Table 1).

**Table 1:** Synthesis of 8-allyl-7-hydroxycoumarins **8**.

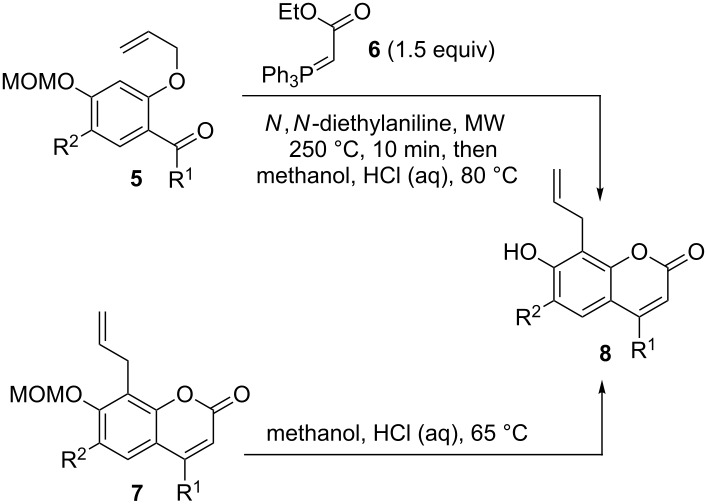						

entry	**5**	R^1^	R^2^	**8**	yield (%)^a^	yield (%)^b^

1	**5a**	H	H	**8a**	59	82
2	**5b**	C_6_H_5_	H	**8b**	70	91
3	**5c**	H	OCH_3_	**8c**	40	84
4	**5d**	CH_3_	H	**8d**	66	89

^a^Yield starting from **5** without isolation of **7**. ^b^Yield starting from **7**.

For the synthesis of furanocoumarins **3** and their ring-expanded oxepino analogues **11** the 8-allylcoumarins **8** were first O-allylated. The resulting allyl ethers **9** underwent ring-closing metathesis to oxepines **11** smoothly in the presence of second-generation Grubbs’ catalyst **A** [54] within one hour at 90 °C, except for the 4-phenyl-substituted derivative **9b**, which was recovered from the reaction mixture under these conditions. However, compound **9b** was successfully cyclized to **11b** using catalyst **A** in dichloromethane at ambient temperature, higher dilution and after prolonged reaction time. For the synthesis of furanocoumarins **3** the allyl ethers **9** were first subjected to a Ru hydride-catalyzed double bond isomerization [55–56] to furnish enol ethers **10** as inseparable mixtures of diastereoisomers. For these reasons a complete structural assignment turned out to be difficult, but the products with a 7-*Z*-propenyloxy- and an 8-*E*-propenyl substituent, as shown in Table 2, were in all cases predominant, followed by the *E*,*E*-configured products. The ratio of these two isomers was ca. 3:1 for compounds **10a**,**b**,**d** and ca. 10:1 for **10c** with an adjacent coordinating methoxy group. The other two diastereoisomers were present only in trace amounts.

**Table 2:** Synthesis of oxepino- **11** and furanocoumarins **3** from a common precursor **9**.

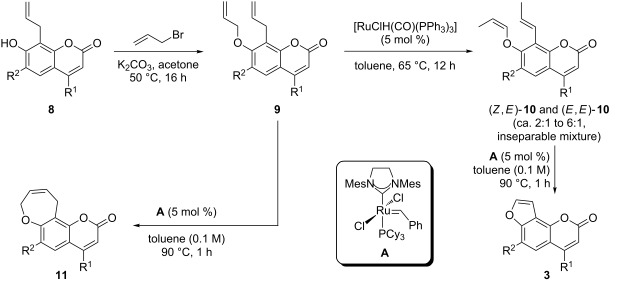											

entry	**8**	R^1^	R^2^	**9**	yield (%)	**10**	yield (%)	**11**	yield (%)	**3**	yield (%)

1	**8a**	H	H	**9a**	97	**10a**	93	**11a**	92	**3a**	95
2	**8b**	C_6_H_5_	H	**9b**	92	**10b**	94	**11b**	79^a^	**3b**	89
3	**8c**	H	OCH_3_	**9c**	95	**10c**	quant.	**11c**	79	**3c**	98
4	**8d**	CH_3_	H	**9d**	91	**10d**	quant.	**11d**	77	**3d**	quant.

^a^**A** (5 mol %), CH_2_Cl_2_ (0.05 M), 20 °C.

RCM of enol ethers [57–58] **10** under the same conditions used for the synthesis of the oxepino-annellated coumarins **11** gave furanocoumarins **3** in excellent yields (Table 2). Furanocoumarins **3a** (angelicin or isopsoralen, Table 2, entry 1) [11–12] and **3c** (sphondin, Table 2, entry 3) [59–60] are natural products. They have previously been synthesized from 7-hydroxy-8-iodocoumarins through Sonogashira coupling and cyclization [61] or via Dötz benzannellation [62] of furanyl carbene complexes and acetylenes [63]. Angelicin (**3a**) was also obtained via RCM of 8-(1-propenyl)-7-vinyloxycoumarin, but the synthesis of this precursor required four steps, starting from umbelliferone, and proceeded only with moderate regioselectivity for the second step [47].

**Scheme 1:**
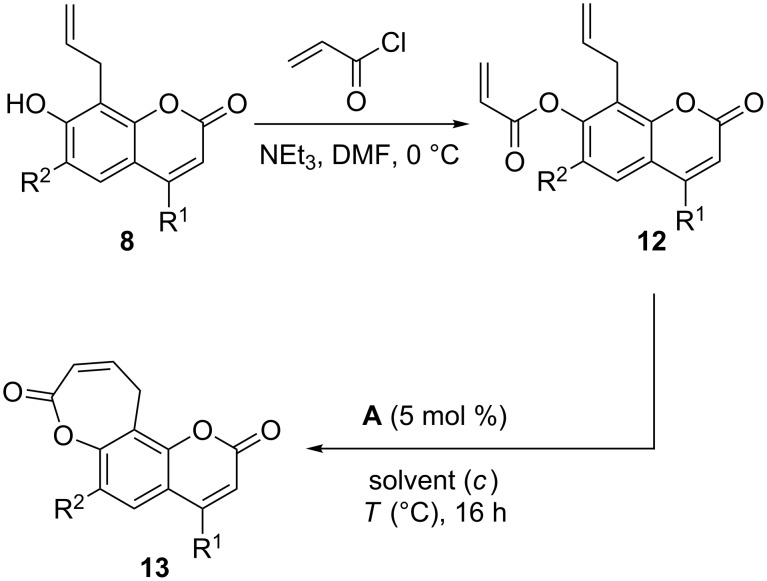
Synthesis of oxepin-2-one-annellated coumarins **13** by RCM of acrylates **12**.

Next, we investigated the synthesis of coumarins with annellated unsaturated lactones starting from the same 8-allylcoumarins **8** (Scheme 1). For the synthesis of oxepin-2-one-annellated coumarins **13** compounds **8** were first converted to the corresponding acrylates **12** with acryloyl chloride (Table 3). RCM of these acrylates turned out to be not straightforward but required some optimization (Table 4).

**Table 3:** Synthesis of acrylates **12**.

entry	**8**	R^1^	R^2^	**12**	yield (%)

1	**8a**	H	H	**12a**	92
2	**8b**	C_6_H_5_	H	**12b**	93
3	**8c**	H	OCH_3_	**12c**	89
4	**8d**	CH_3_	H	**12d**	86

**Table 4:** Optimization of RCM conditions and synthesis of annellated coumarins **13**.

entry	**12**	R^1^	R^2^	solvent	*c* (mol·L^−1^)	*T* (° C)	**13**	yield (%)

1	**12a**	H	H	CH_2_Cl_2_	0.05	20	**13a**	16
2	**12a**	H	H	CH_2_Cl_2_	0.01	40	**13a**	35
3^a^	**12a**	H	H	CH_2_Cl_2_	0.01	40	**13a**	–^b^
4^c^	**12a**	H	H	CH_2_Cl_2_	0.01	40	**13a**	–^b^
5	**12a**	H	H	toluene	0.01	20	**13a**	20
6	**12a**	H	H	toluene	0.01	110	**13a**	81
7	**12b**	C_6_H_5_	H	toluene	0.01	110	**13b**	79
8	**12c**	H	OCH_3_	toluene	0.01	110	**13c**	91
9	**12d**	CH_3_	H	toluene	0.01	110	**13d**	86

^a^Additive Ti(OiPr)_4_ (1.0 equiv). ^b^No conversion. ^c^Additive Ti(OiPr)_4_ (2.0 equiv).

In particular, a reduced initial substrate concentration of 0.01 M and reaction temperatures of 110 °C (Table 4, entry 6) led to a smooth conversion to the desired oxepin-2-ones **13**, whereas ambient or slightly elevated temperatures in CH_2_Cl_2_ or toluene as a solvent (Table 4, entries 1, 2 and 5) resulted in incomplete conversions and low yields. Addition of the Lewis acid Ti(OiPr)_4_, which had previously been reported to prevent the formation of inactive catalyst–substrate chelates [64], inhibited the RCM reaction completely in this case (Table 4, entries 3 and 4). The beneficial effect of low initial substrate concentrations on RCM reactions with acrylates has previously been described [65] and was later systematically investigated by one of us [66].

**Scheme 2:**
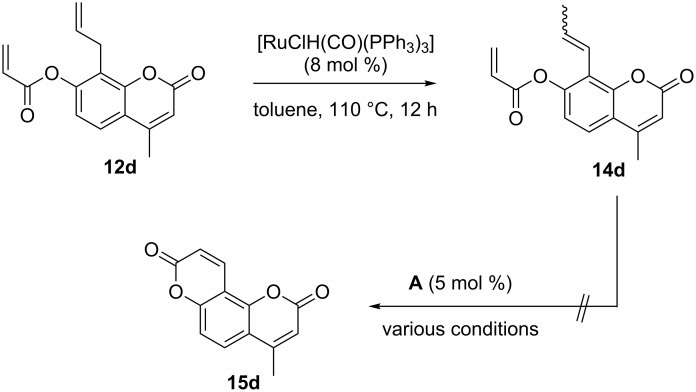
Attempted synthesis of pyran-2-one-annellated coumarin **15d** via isomerization-RCM.

A possible access to the pyran-2-one-annellated coumarin substitution pattern **15** was investigated starting from acrylate **12d** (Scheme 2). The isomerization of the 8-allyl substituent to a prop-1-enyl substituent under the conditions used for the synthesis of precursors **10** (Table 2) stopped at 50% conversion. Higher catalyst loading and an increased reaction temperature, however, resulted in a quantitative conversion to **14d** as a mixture of *E*- and *Z*-isomers. Although the RCM of similarly substituted acrylates to coumarins was previously described in the literature, this reaction failed completely for the envisaged synthesis of **15d** from **14d** under various conditions. Initial substrate concentrations varying from 0.01 M to 0.10 M, the solvents dichloromethane and toluene, and reaction temperatures between ambient temperature and 110 °C were tested, but to no avail.

As a method to circumvent notoriously difficult acrylate RCM steps we [45] and others [67] have developed an assisted tandem catalytic [68] RCM/allylic oxidation sequence. Such tandem sequences are characterized by the combination of two mechanistically distinct catalytic reactions in a defined order, which proceed with a single precatalyst that undergoes a transformation in situ upon addition of a suitable reagent, a “chemical trigger” [69]. In the case of the RCM/allylic oxidation sequence *tert*-butyl hydroperoxide is added after completion of the metathesis reaction, which most likely induces a conversion of the metathesis active Ru–carbene species to a Ru(IV)–oxo species. The latter are known to catalyze allylic and benzylic oxidation reactions through a radical mechanism [70].

To implement this tandem sequence in the synthesis of pyran-2-one-annellated coumarins **15** an isomerization of the 8-allyl substituent to a prop-1-enyl substituent was first required. When 8-allyl-7-hydroxycoumarin (**8a**) was subjected to the isomerization conditions previously used for the synthesis of furanocoumarin precursors **10** (see Table 2) we observed no conversion. A plausible explanation is the formation of a stable six-membered Ru–O–chelate complex following hydroruthenation, which inhibits a subsequent β-hydride elimination and thus interrupts the catalytic cycle. For these reasons we started from the MOM-protected 8-allylcoumarins **7**, which underwent the Ru-hydride catalyzed double bond migration smoothly. The MOM group was cleaved off without isolation of the intermediate products and the required 7-hydroxy-8-(prop-1-enyl)coumarins **16** were isolated in high overall yields and *E*-selectivities. Allylation of phenols **16** furnished the RCM precursors **17**, which underwent the tandem RCM/allylic oxidation sequence to compounds **15** in fair yields (Table 5). All pyran-2-one-annellated coumarins **15** synthesized in the course of this study were previously described in the literature: compound **15a** was used to investigate the regioselectivity of [2 + 2]-photocycloadditions [71], compound **15d** was included in a comparative investigation into the fluorescence properties of 4-methylcoumarins [72], compounds **15b** and **15c** were tested for bacteriostatic activity [73] and insect-antifeedant activity [74], respectively. A common denominator of these reports is that a thorough investigation into the spectral and biological properties of these compounds is hampered by unsatisfactory yields and selectivities if classical coumarin syntheses are used. Compound **15a**, for instance, was obtained in only 14% yield from umbelliferone and malic acid in a Pechmann synthesis [71].

**Table 5:** Synthesis of pyran-2-one-annellated coumarins **15** via tandem RCM/allylic oxidation.

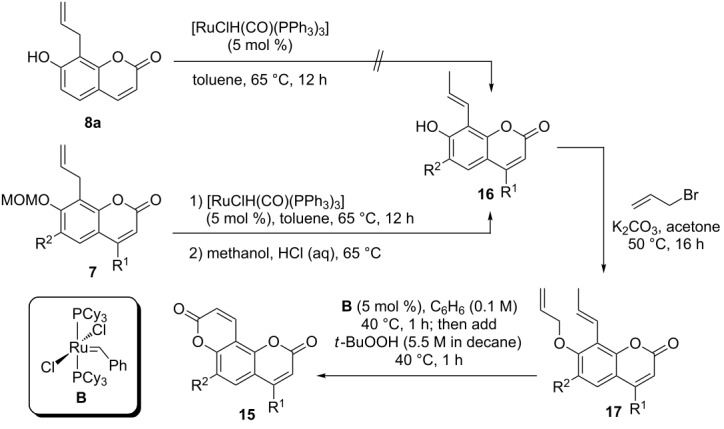									

entry	**7**	R^1^	R^2^	**16**	yield (%)	**17**	yield (%)	**15**	yield (%)

1	**7a**	H	H	**16a**	95	**17a**	93	**15a**	56
2	**7b**	C_6_H_5_	H	**16b**	81	**17b**	89	**15b**	41
3	**7c**	H	OCH_3_	**16c**	quant.	**17c**	90	**15c**	45
4	**7d**	CH_3_	H	**16d**	92	**17d**	quant.	**15d**	47

**Scheme 3:**
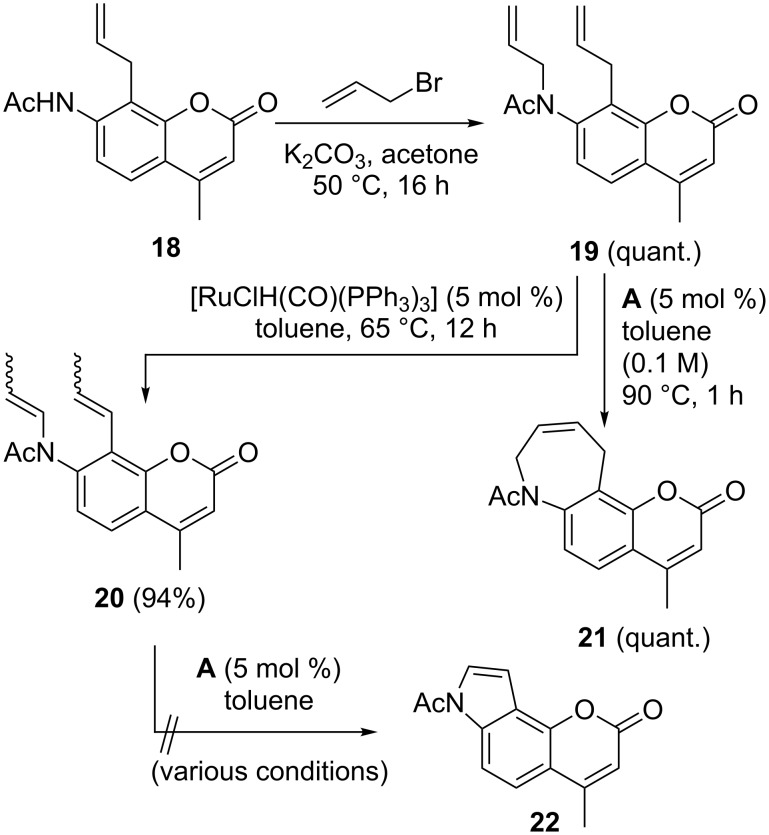
Synthesis of aza-annellated coumarin **21** and attempted synthesis of indole **22**.

We concluded our study by investigating the possibility to transfer the syntheses of oxa-annellated coumarins described above to the aza-annellated derivatives. Starting point was the 7-acetamido-substituted coumarin **18** [29], which was first N-allylated to the allylamide **19**. Dual double bond migration was accomplished with the Ru–hydride complex used previously and furnished the enamide **20** in high yield and predominantly as the *E*,*E*-isomer. In light of previous work by Arisawa et al. [75], who reported a synthesis of indoles by RCM of sterically less encumbered enamides, we investigated the RCM of **20**. Unfortunately, no conversion to the indole **22** could be observed under various conditions. Ring-closing metathesis of **19** was, in contrast, successful and furnished the azepinocoumarin **21** in quantitative yield (Scheme 3).

## Conclusion

In summary, we demonstrated that annellated coumarins become accessible from appropriately substituted benzene derivatives in few steps, using a microwave-promoted tandem Wittig olefination/Claisen rearrangement/cyclization sequence for the construction of the 8-allylcoumarin scaffold and combinations of double bond isomerization and ring-closing olefin metathesis for the annellation of a second heterocycle. Pyran-2-one-annellated coumarins, which are scarcely available in synthetically useful yields through classical methods, became accessible through a tandem RCM/allylic oxidation sequence.

## Supporting Information

File 1 Full experimental procedures, characterization data and copies of ^1^H and ^13^C NMR spectra of all compounds.
