# Supplementary material for: Ring-closing-metathesis-based synthesis of annellated coumarins from 8-allylcoumarins
Source: Beilstein J Org Chem. 2018 Dec 5;14:2991–8. doi: 10.3762/bjoc.14.278 (PMC6296409; doi:10.3762/bjoc.14.278)

BEILSTEIN JOURNAL OF ORGANIC CHEMISTRY

Supporting Information

for

Ring-closing-metathesis-based synthesis of annellated coumarins from 8-allylcoumarins

Christiane Schultze and Bernd Schmidt

Beilstein J. Org. Chem. **2018,** *14,* 2991–2998. doi:10.3762/bjoc.14.278

Full experimental procedures, characterization data and copies of ^{1}H and ^{13}C NMR spectra of all compounds

License and Terms: This is a supporting information file under the terms of the Creative Commons Attribution License (http://creativecommons.org/licenses/by/4.0). Please note that the reuse, redistribution and reproduction in particular requires that the authors and source are credited.
The license is subject to the *Beilstein Journal of Organic Chemistry* terms and conditions: (https://www.beilstein-journals.org/bjoc)

Contents:

General methods S3

General procedures and analytical data S3

References S25

Copies of spectra S27

General methods. All experiments were conducted in dry reaction vessels under an atmosphere of dry nitrogen. Solvents were purified by standard procedures. ^{1}H NMR spectra were obtained at 300 MHz in $CDCl_3$ with $C\mathit{H}Cl_3$ (δ = 7.26 ppm) as an internal standard. Coupling constants are given in Hz. ^{13}C NMR spectra were recorded at 75 MHz in $CDCl_3$ with $CDCl_3$ (δ = 77.1 ppm) as an internal standard. Whenever the solubility or stability of the sample or signal separation were insufficient in $CDCl_3$, it was replaced by one of the following solvents: acetone-d_6 (acetone-d_5 as internal standard for ^{1}H NMR spectroscopy, δ = 2.05 ppm, $\mathit{C}D_3COCD_3$ as internal standard for ^{13}C NMR spectroscopy, δ = 29.8 ppm); DMSO-d_6 (DMSO-d_5 as internal standard for ^{1}H NMR spectroscopy, δ = 2.50 ppm, DMSO-d_6 as internal standard for ^{13}C NMR spectroscopy, δ = 39.5 ppm). IR spectra were recorded as ATR-FTIR spectra. Wavenumbers (ν) are given in cm^{-1}. The peak intensities are defined as strong (s), medium (m) or weak (w). Low- and high-resolution mass spectra were obtained by EI–TOF or ESI–TOF. The general procedures for the synthesis of compounds **8**, **9** and **10** stated below follow closely procedures previously reported by us [1].

General procedure for the synthesis of coumarins 8 from MOM-protected coumarins 7. To a solution of the appropriate MOM-ether **7** (1.00 mmol) in methanol (20 mL) was added aq. HCl (3 M, 100 μL) and the solution was heated to 65 °C for 1 h. Upon cooling to ambient temperature, water (30 mL) and ethyl acetate (30 mL) were added. The organic layer was separated, and the aqueous layer extracted twice with ethyl acetate (30 mL each). The combined organic extracts were dried with $MgSO_4$, filtered, and evaporated. The residue was purified by column chromatography on silica, using hexanes-MTBE mixtures of increasing polarity.

General procedure for the synthesis of 8-allylcoumarins 8 from allyl ethers 5. Ylide **6** (522 mg, 1.50 mmol) and the corresponding allyl ether **5** (1.00 mmol) were dissolved in *N*,*N*-

diethylaniline (10 mL) in a vessel suitable for microwave irradiation. The vessel was sealed and irradiated in a dedicated microwave reactor for 10 min. at 250 °C. Upon cooling to ambient temperature the mixture was diluted with ethyl acetate (50 mL) and washed three times with aq. HCl (2 M, 30 mL each). After evaporation of all volatiles the residue was redissolved in methanol (20 mL) and aq. HCl (100 µL) was added. The mixture was heated to 65 °C for 1 h and then cooled to ambient temperature. From this point work-up was carried out as described above for the synthesis starting from compounds **7**.

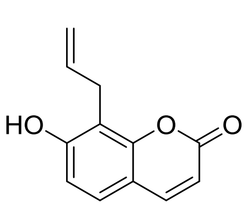

8a

8-Allyl-7-hydroxy-2*H*-chromen-2-one (8a)[1]. Starting from **7a** (246 mg, 1.00 mmol) compound **8a** (166 mg, 0.82 mmol, 82%) was obtained. Starting from **5a** (222 mg, 1.00 mmol) compound **8a** (119 mg, 0.59 mmol, 59%) was obtained. Colourless solid, mp 162 – 163 °C (literature: 162 – 163 °C[1]). Other analytical data have been published previously[1].

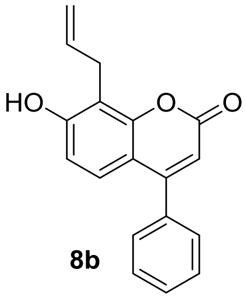

8b

8-Allyl-7-hydroxy-4-phenyl-2*H*-chromen-2-one (8b)[2]. Starting from **7b** (322 mg, 1.00 mmol) compound **8b** (253 mg, 0.91 mmol, 91%) was obtained. Starting from **5b** (298 mg, 1.00 mmol) compound **8b** (195 mg, 0.70 mmol, 70%) was obtained. Yellowish solid, mp 190 - 193 °C (literature: 190 – 192 °C[3]); ^{1}H NMR (300 MHz, acetone-d_6) δ 7.59 – 7.48 (5H), 7.19 (d, J = 8.8 Hz, 1H), 6.89 (d, J = 8.8 Hz, 1H), 6.09 (s, 1H), 6.02 (ddt, J = 16.4, 10.0, 6.3 Hz, 1H), 5.09 (dm, J = 17.1 Hz, 1H), 4.98 (dm, J = 10.0 Hz, 1H), 3.61 (dm, J = 6.3 Hz, 2H); ^{13}C NMR (75 MHz, acetone-d_6) δ 161.0, 159.6, 157.0, 154.7, 136.9, 136.2, 130.2, 129.6, 129.3, 126.6, 115.6, 114.9, 112.8, 112.6, 111.5, 27.7; IR (ATR) ν 3210 (bm), 1683 (s), 1600 (s), 1561 (s), 1444 (w), 1372 (s), 1312 (s), 1111 (m), 1058 (m); HRMS (EI) calcd for $C_{18}H_{14}O_3$ [M^+] 278.0943, found 278.0946.

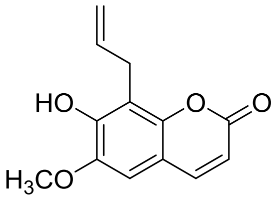

8c

8-Allyl-7-hydroxy-6-methoxy-2*H*-chromen-2-one (8c)[4]. Starting from **7c** (276 mg, 1.00 mmol) compound **8c** (195 mg, 0.84 mmol, 84%) was obtained. Starting from **5c** (252 mg, 1.00 mmol) compound **8c** (93 mg, 0.40 mmol, 40%) was obtained. Yellowish solid, mp 146 - 148 °C (no mp or other data reported in the literature); ^{1}H NMR (300 MHz, $CDCl_3$) δ 7.57 (d, *J* = 9.4 Hz, 1H), 6.74 (s, 1H), 6.37 (s, 1H), 6.23 (d, *J* = 9.4 Hz, 1H), 5.98 (ddt, *J* = 16.6, 10.0, 6.3 Hz, 1H), 5.09 (dm, *J* = 17.1 Hz, 1H), 4.99 (dm, *J* = 10.0 Hz, 1H), 3.92 (s, 3H), 3.60 (d, *J* = 6.3 Hz, 2H); ^{13}C NMR (75 MHz, $CDCl_3$) δ 161.7, 148.4, 147.8, 143.9, 143.8, 134.8, 115.7, 114.4, 113.1, 111.2, 105.6, 56.5, 27.1; IR (ATR) ν 3335 (bm), 3016 (w), 1703 (s), 1605 (m), 1570 (s), 1489 (m), 1419 (m), 1282 (s), 1152 (m), 1094 (m); HRMS (EI) calcd for $C_{13}H_{12}O_4$ [M^+] 232.0736, found 232.0732.

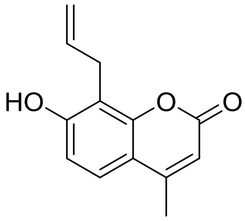

8d

8-Allyl-7-hydroxy-4-methyl-2*H*-chromen-2-one (8d)[5]. Starting from **7d** (260 mg, 1.00 mmol) compound **8d** (192 mg, 0.89 mmol, 89%) was obtained. Starting from **5d** (236 mg, 1.00 mmol) compound **8d** (143 mg, 0.66 mmol, 66%) was obtained. Yellowish solid, mp 193 - 195 °C (literature: 198 – 199 °C[5]); ^{1}H NMR (300 MHz, DMSO-d_6) δ 10.44 (s, 1H), 7.46 (d, *J* = 8.7 Hz, 1H), 6.87 (d, *J* = 8.7 Hz, 1H), 6.10 (s, 1H), 5.90 (ddt, *J* = 17.9, 9.3, 6.1 Hz, 1H), 4.97 – 4.89 (m, 2H), 3.43 (d, *J* = 6.1 Hz, 2H), 2.34 (d, *J* = 1.0 Hz, 3H); ^{13}C NMR (75 MHz, DMSO-d_6) δ 160.3, 158.7, 153.7, 152.6, 135.4, 123.9, 115.0, 112.7, 112.0, 111.9, 110.0, 26.5, 18.2; IR (ATR) ν 3217 (bw), 3006 (w), 1683 (m), 1603 (m), 1572 (m), 1385 (m), 1311 (w), 1275 (m); HRMS (EI) calcd for $C_{13}H_{12}O_3$ [M^+] 216.0786, found 216.0794.

General procedure for the synthesis of allyl ethers 9. The corresponding 7-hydroxycoumarin 8 (1.00 mmol) and allyl bromide (128 μL, 1.50 mmol) were dissolved in acetone (5 mL) and K_2CO_3 (280 mg, 2.00 mmol) was added. The mixture was heated to 50 °C and stirred for 16 h, cooled to ambient temperature, and brine (20 mL) and ethyl acetate (30

mL) were added. The organic layer was separated and the aqueous layer was extracted twice with ethyl acetate (30 mL each). The combined organic extracts were dried with $MgSO_4$, filtered and evaporated. The residue was purified by column chromatography on silica using hexanes-MTBE mixtures of increasing polarity.

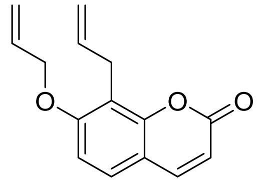

9a

8-Allyl-7-(allyloxy)-2*H*-chromen-2-one (9a)[6]. Starting from **8a** (202 mg, 1.00 mmol) compound **9a** (235 mg, 0.97 mmol, 97%) was obtained. Yellowish solid, mp 96 - 98 °C (literature: 82 – 84 °C[6]); ^{1}H NMR (300 MHz, $CDCl_3$) δ 7.61 (d, J = 9.5 Hz, 1H), 7.29 (d, J = 8.6 Hz, 1H), 6.82 (d, J = 8.6 Hz, 1H), 6.23 (d, J = 9.4 Hz, 1H), 6.12 – 5.89 (m, 2H), 5.43 (dm, J = 17.3 Hz, 1H), 5.30 (dm, J = 10.6 Hz, 1H), 5.08 (dm, J = 17.1 Hz, 1H), 4.98 (dm, J = 10.0 Hz, 1H), 4.63 (dm, J = 5.0 Hz, 2H), 3.62 (dm, J = 6.4 Hz, 2H); ^{13}C NMR (75 MHz, $CDCl_3$) δ 161.4, 159.4, 153.1, 143.8, 135.2, 132.6, 126.7, 117.8, 116.6, 115.6, 113.2, 113.1, 108.7, 69.4, 27.1; IR (ATR) ν 3069 (w), 2980 (w), 1715 (s), 1602 (s), 1493 (m), 1408 (m), 1275 (s), 1245 (s), 1116 (s), 1066 (s), 1027 (s); HRMS (EI) calcd for $C_{15}H_{14}O_3$ [M^+] 242.0943, found 242.0954.

8-Allyl-7-(allyloxy)-4-phenyl-2*H*-chromen-2-one (9b). Starting from **8b** (278 mg, 1.00 mmol) compound **9b** (293 mg, 0.92 mmol, 92%) was obtained. Orange-coloured solid, mp 94 – 96 °C; ^{1}H NMR (300 MHz, $CDCl_3$) δ 7.58 – 7.50 (m, 3H), 7.49 – 7.42 (m, 2H), 7.33 (d, J = 8.9 Hz, 1H), 6.80 (d, J = 8.9 Hz, 1H), 6.24 (s, 1H), 6.14 – 5.97 (m, 2H), 5.46 (dm, J = 17.3 Hz, 1H), 5.33 (dm, J = 10.6 Hz, 1H), 5.17 (dm, J = 17.1 Hz, 1H), 5.05 (dm, J = 10.0 Hz, 1H), 4.67 (dm, J = 4.9 Hz, 2H), 3.72 (dm, J = 6.4 Hz, 2H); ^{13}C NMR (75 MHz, $CDCl_3$) δ 161.3, 159.3, 156.1, 153.2, 135.9, 135.3, 132.7, 129.6, 128.9, 128.5, 125.9, 117.8, 116.8, 115.6, 113.2, 112.1, 108.4, 69.4, 27.3; IR (ATR) ν 3078 (w), 2979 (w), 1717 (s), 1596 (s), 1490 (m), 1445 (m), 1371 (s), 1275 (s), 1116 (s), 1068 (m), 991 (m); HRMS (EI) calcd for $C_{21}H_{18}O_3$ [M^+] 318.1256, found 318.1248.

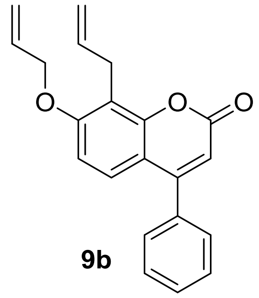

9b

8-Allyl-7-(allyloxy)-6-methoxy-2-*H*-chromen-2-one (9c). Starting from **8c** (232 mg, 1.00 mmol) compound **9c** (258 mg, 0.95 mmol, 95%) was obtained. Yellowish solid, mp 73 - 75 °C; ^{1}H NMR (300 MHz, $CDCl_3$) δ 7.61 (d, *J* = 9.5 Hz, 1H), 6.81 (s, 1H), 6.31 (d, *J* = 9.5 Hz, 1H), 6.15 – 5.91 (m, 2H), 5.38 (dm, *J* = 17.2 Hz, 1H), 5.24 (dm, *J* = 10.4 Hz, 1H), 5.06 (dm, *J* = 17.1 Hz, 1H), 5.00 (dm, *J* = 10.0 Hz, 1H), 4.58 (dm, *J* = 5.8 Hz, 2H), 3.88 (s, 3H), 3.63 (dm, *J* = 6.3 Hz, 2H); ^{13}C NMR (75 MHz, $CDCl_3$) δ 161.3, 150.0, 149.7, 147.6, 143.5, 135.5, 133.8, 122.9, 118.0, 115.9, 115.0, 114.7, 107.5, 74.4, 56.2, 28.0; IR (ATR) ν 3079 (w), 2938 (w), 1716 (s), 1605 (m), 1565 (s), 1480 (m), 1462 (m), 1402 (s), 1285 (s), 1142 (s), 1100 (s), 1057 (m); HRMS (EI) calcd for $C_{16}H_{16}O_4$ [M^+] 272.1049, found 242.1054.

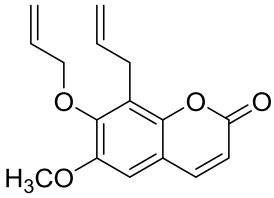

9c

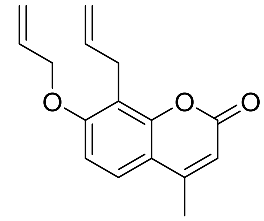

9d

8-Allyl-7-(allyloxy)-4-methyl-2*H*-chromen-2-one (9d).[7] Starting from **8d** (216 mg, 1.00 mmol) compound **9d** (233 mg, 0.91 mmol, 91%) was obtained. Yellowish solid, mp 92 – 94 °C (literature: 94 °C[7]); ^{1}H NMR (300 MHz, $CDCl_3$) δ 7.42 (d, *J* = 8.8 Hz, 1H), 6.83 (d, *J* = 8.8 Hz, 1H), 6.11 (s, 1H), 6.09 – 5.88 (m, 2H), 5.43 (dm, *J* = 17.3 Hz, 1H), 5.30 (dm, *J* = 10.6 Hz, 1H), 5.07 (dm, *J* = 17.1 Hz, 1H), 4.97 (dm, *J* = 10.0 Hz, 1H), 4.64 (dm, *J* = 4.9 Hz, 2H), 3.63 (dm, *J* = 6.4 Hz, 2H), 2.37 (d, *J* = 1.0 Hz, 3H); ^{13}C NMR (75 MHz, $CDCl_3$) δ 161.4, 159.2, 152.7, 152.6, 135.3, 132.7, 123.3, 117.7, 116.6, 115.5, 114.1, 112.2, 108.4, 69.4, 27.2, 18.8; IR (ATR) ν 3092 (w), 2973 (w), 1709 (s), 1602 (s), 1567 (s), 1503 (m), 1432 (m), 1382 (s), 1277 (s), 1223 (s), 1120 (s), 1054 (s); HRMS (EI) calcd for $C_{16}H_{16}O_3$ [M^+] 256.1099, found 256.1095.

General procedure for the synthesis of enol ethers 10. To a solution of the corresponding allyl ether **9** (1.00 mmol) in toluene (10 mL) was added [$RuClH(CO)(PPh_3)_3$] (48 mg, 0.05 mmol, 5 mol %). The solution was heated to 65 °C and stirred for 12 h, cooled to ambient

temperature and all volatiles were evaporated in vacuo. The residue was purified by column chromatography on silica using hexanes-MTBE mixtures of increasing polarity as eluent.

8-(Prop-1-en-1-yl)-7-(prop-1-en-1-yloxy)-2*H*-chromen-2-one (10a). Starting from **9a** (242 mg, 1.00 mmol) compound **10a** (225 mg, 0.93 mmol, 93%) was obtained as a mixture of diastereoisomers. Brownish oil; NMR-data of the major isomer (*Z*,*E*)-**10a** obtained from the mixture: ^{1}H NMR (300 MHz, $CDCl_3$) δ 7.64 (d, J = 9.5 Hz, 1H), 7.26 (d, J = 8.6 Hz, 1H), 6.97 – 6.82 (m, 2H), 6.80 – 6.68 (m, 1H), 6.44 – 6.36 (m, 1H), 6.30 (d, J = 9.4 Hz, 1H), 5.06 (dq, J = 6.8, 6.5 Hz, 1H), 2.00 (dd, J = 6.3, 1.2 Hz, 3H), 1.77 (dd, J = 6.9, 1.8 Hz, 3H); ^{13}C NMR (75 MHz, $CDCl_3$) δ 160.9, 157.5, 152.4, 143.8, 140.1, 134.1, 126.1, 118.8, 114.1, 113.7, 111.4, 109.7, 20.2, 9.6, one quaternary carbon was not unambigously assignable. Characteristic signals of (*E*,*E*)-**10a** obtained from the mixture: ^{1}H NMR (300 MHz, $CDCl_3$) δ 5.50 (dq, J = 12.1, 7.0 Hz, 1H); ^{13}C NMR (75 MHz, $CDCl_3$) δ 12.2.

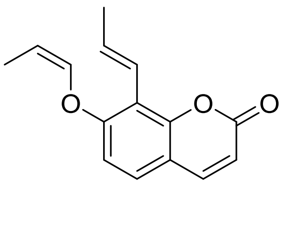

10a

4-Phenyl-8-(prop-1-en-1-yl)-7-(prop-1-en-1-yloxy)-2*H*-chromen-2-one (10b). Starting from **9b** (318 mg, 1.00 mmol) compound **10b** (299 mg, 0.94 mmol, 94%) was obtained as a mixture of diastereoisomers. Brownish solid; NMR-data of the major isomer (*Z*,*E*)-**10b** obtained from the mixture: ^{1}H NMR (300 MHz, $CDCl_3$) δ 7.54 – 7.48 (m, 3H), 7.45 – 7.39 (m, 2H), 7.23 (d, J = 9.0 Hz, 1H), 6.96 – 6.74 (m, 3H), 6.42 – 6.43 (m, 1H), 6.26 (s, 1H), 5.04 (dq, J = 6.9, 6.5 Hz, 1H), 2.05 – 1.97 (m, 3H), 1.75 (dd, J = 6.9, 1.6 Hz, 3H); ^{13}C NMR (150 MHz, $CDCl_3$) δ 161.1, 157.6, 156.2, 152.6, 140.2, 135.9, 134.3, 129.6, 128.9, 128.6, 125.5, 119.1, 115.9, 114.3, 112.7, 111.1, 109.9, 20.4, 9.8. Characteristic signals of (*E*,*E*)-**10b** obtained from the mixture: ^{1}H NMR (300 MHz, $CDCl_3$) δ 5.48 (dq, J = 12.1, 7.0 Hz, 1H); ^{13}C NMR (150 MHz, $CDCl_3$) δ 12.4.

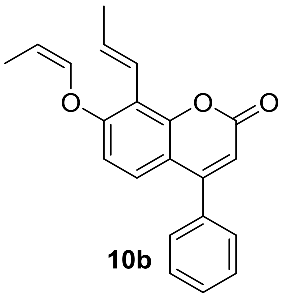

10b

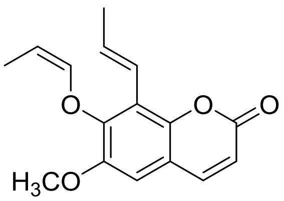

10c

6-Methoxy-8-(prop-1-en-1-yl)-7-(prop-1-en-1-yloxy)-2*H*-chromen-2-one (10c). Starting from **9c** (272 mg, 1.00 mmol) compound **10c** (272 mg, 1.00 mmol, quant.) was obtained as a mixture of diastereoisomers. Brownish solid; NMR-data of the major isomer (*Z*,*E*)-**10c** obtained from the mixture: ^{1}H NMR (300 MHz, $CDCl_3$) δ 7.62 (d, *J* = 9.5 Hz, 1H), 6.90 – 6.74 (m, 2H), 6.62 (dm, *J* = 16.1 Hz, 1H), 6.36 (d, *J* = 9.4 Hz, 1H), 6.09 (dq, *J* = 6.1, 1.6 Hz, 1H), 4.68 (dq, *J* = 6.7, 6.5 Hz, 1H), 3.87 (s, 3H), 1.97 (dd, *J* = 6.6, 1.8 Hz, 3H), 1.79 (dd, *J* = 6.9, 1.6 Hz, 3H); ^{13}C NMR (150 MHz, $CDCl_3$) δ 161.0, 160.9, 149.3, 144.6, 143.7, 135.3, 120.9, 119.1, 115.4, 115.3, 107.8, 103.5, 56.7, 20.3, 9.3, one quaternary carbon not observed due to signal overlap. Characteristic signals of (*E*,*E*)-**10c** obtained from the mixture: ^{1}H NMR (300 MHz, $CDCl_3$) δ 4.96 (dq, *J* = 12.4, 7.0 Hz, 1H).

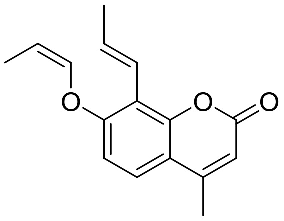

10d

4-Methyl-8-(prop-1-en-1-yl)-7-(prop-1-en-1-yloxy)-2*H*-chromen-2-one (10d). Starting from **9d** (256 mg, 1.00 mmol) compound **10d** (256 mg, 1.00 mmol, quant.) was obtained as a mixture of diastereoisomers. Brownish oil; NMR-data of the major isomer (*Z*,*E*)-**10d** obtained from the mixture: ^{1}H NMR (300 MHz, $CDCl_3$) δ 7.36 (d, *J* = 8.9 Hz, 1H), 6.90 (d, *J* = 8.9 Hz, 1H), 6.87 – 6.65 (m, 2H), 6.43 – 6.33 (m, 1H), 6.16 (s, 1H), 5.03 (dq, *J* = 6.8, 6.2 Hz, 1H), 2.38 (d, *J* =1.0 Hz, 3H), 1.98 (dd, *J* = 5.7, 0.8 Hz, 3H), 1.74 (dd, *J* = 6.9, 1.8 Hz, 3H); ^{13}C NMR (150 MHz, $CDCl_3$) δ 161.1, 157.4, 152.8, 151.9, 140.3, 134.1, 122.8, 119.1, 115.2, 112.7, 111.1, 110.7, 109.7, 20.3, 19.1, 9.7. Characteristic signals of (*E*,*E*)-**10d** obtained from the mixture: ^{1}H NMR (300 MHz, $CDCl_3$) δ 5.47 (dq, *J* = 12.2, 7.0 Hz, 1H); ^{13}C NMR (150 MHz, $CDCl_3$) δ 12.4.

General procedure for the synthesis of furanocoumarins 3 and oxepinocoumarins 11. The appropriate precursors **9** or **10** (1.00 mmol) were dissolved in toluene (10 mL) and precatalyst **A** (42 mg, 0.05 mmol, 5 mol %) was added. The mixture was heated to 90 °C until

TLC indicated complete conversion (ca. 1 h), cooled to ambient temperature and evaporated in vacuo. The residue was purified by column chromatography on silica using hexanes-MTBE mixtures of increasing polarity as eluents to furnish the products **3** or **11**, respectively.

8,11-Dihydro-2*H*-oxepino[2,3-*h*]chromen-2-one (11a)[6]. Starting from **9a** (242 mg, 1.00 mmol) compound **11a** (197 mg, 0.92 mmol, 92%) was obtained. Colourless solid, mp 103 - 105 °C (literature: 119 – 121 °C[6]); ^{1}H NMR (300 MHz, $CDCl_3$) δ 7.62 (d, *J* = 9.5 Hz, 1H), 7.20 (d, *J* = 8.5 Hz, 1H), 7.07 (dm, *J* = 12.2 Hz, 1H), 6.88 (d, *J* = 8.5 Hz, 1H), 6.27 (d, *J* = 9.5 Hz, 1H), 6.17 (dt, *J* = 12.1, 4.5 Hz, 1H), 4.30 (t, *J* = 4.9 Hz, 2H), 2.75 (qd, *J* = 4.6, 1.9 Hz, 2H); ^{13}C NMR (75 MHz, $CDCl_3$) δ 162.6, 160.9, 153.0, 144.0, 132.7, 126.7, 119.0, 117.1, 115.5, 113.9, 113.5, 70.6, 34.3; IR (ATR) ν 2965 (w), 2890 (w), 1712 (s), 1590 (s), 1490 (m), 1404 (m), 1338 (m), 1286 (m), 1229 (s), 1109 (s), 1067 (m); HRMS (EI) calcd for $C_{13}H_{10}O_3$ [M^+] 214.0630, found 214.0625.

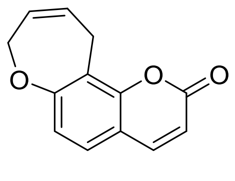

11a

4-Phenyl-8,11-dihydro-2*H*-oxepino[2,3-*h*]chromen-2-one (11b). Starting from **9b** (318 mg, 1.00 mmol) compound **11b** (229 mg, 0.79 mmol, 79%) was obtained. Deviating from the general procedure, the reaction was run in CH_2Cl_2 (20 mL) at ambient temperature. Colourless solid, mp 117 - 120 °C; ^{1}H NMR (300 MHz, $CDCl_3$) δ 7.53 – 7.48 (m, 3H), 7.44 – 7.39 (m, 2H), 7.29 (d, *J* = 8.6 Hz, 1H), 6.93 (d, *J* = 8.6 Hz, 1H), 6.27 (s, 1H), 5.94 (dm, *J* = 11.1 Hz, 1H), 5.58 (dm, *J* = 11.3 Hz, 1H), 4.66 (tm, *J* = 4.1 Hz, 2H), 3.84 (dm, *J* = 5.3 Hz, 2H); ^{13}C NMR (75 MHz, $CDCl_3$) δ 162.2, 161.0, 156.3, 151.7, 135.8, 129.7, 128.9, 128.5, 127.5, 126.3, 126.1, 123.8, 118.0, 115.6, 113.2, 71.0, 22.6; IR (ATR) ν 3057 (w), 2845 (w), 1718 (s), 1592 (s), 1488 (m), 1444 (m), 1368 (s), 1270 (s), 1164 (m), 1069 (s); HRMS (EI) calcd for $C_{19}H_{14}O_3$ [M^+] 290.0943, found 290.0952.

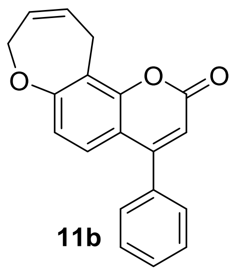

11b

6-Methoxy-8,11-dihydro-2*H*-oxepino[2,3-*h*]chromen-2-one (11c).

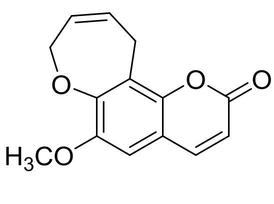

11c

Starting from **9c** (272 mg, 1.00 mmol) compound **11c** (193 mg, 0.79 mmol, 79%) was obtained. Colourless solid, mp 118 - 120 °C; ^{1}H NMR (300 MHz, $CDCl_3$) δ 7.62 (d, *J* = 9.5 Hz, 1H), 6.81 (s, 1H), 6.32 (d, *J* = 9.5 Hz, 1H), 5.88 (dm, *J* = 11.0 Hz, 1H), 5.52 (dm, *J* = 11.4 Hz, 1H), 4.65 (dq, *J* = 4.4, 2.2 Hz, 2H), 3.88 (s, 3H), 3.75 (dq, *J* = 5.4, 1.8 Hz, 2H); ^{13}C NMR (75 MHz, $CDCl_3$) δ 161.2, 151.1, 149.4, 145.6, 143.8, 127.7, 126.0, 125.5, 115.1, 115.0, 107.4, 70.5, 56.4, 22.3; IR (ATR) ν 2938 (w), 2841 (w), 1712 (s), 1604 (m), 1568 (m), 1486 (m), 1406 (s), 1284 (s), 1144 (s), 1108 (s), 1056 (s); HRMS (EI) calcd for $C_{14}H_{12}O_4$ [M^+] 244.0736, found 244.0725.

4-Methyl-8,11-dihydro-2*H*-oxepino[2,3-*h*]chromen-2-one (11d)[6,7]. Starting from **9d** (256 mg, 1.00 mmol) compound **11d** (176 mg, 0.77 mmol, 77%) was obtained. Colourless solid, mp 79 - 81 °C (literature: 109 – 111 °C[6]); ^{1}H NMR (300 MHz, $CDCl_3$) δ 7.42 (d, *J* = 8.6 Hz, 1H), 7.00 (d, *J* = 8.6 Hz, 1H), 6.19 (s, 1H), 5.90 (dm, *J* = 11.0 Hz, 1H), 5.55 (dm, *J* = 11.2 Hz, 1H), 4.64 (dq, *J* = 4.2, 2.1 Hz, 2H), 3.78 (dq, *J* = 5.3, 1.8 Hz, 2H), 2.40 (d, *J* = 1.1 Hz, 3H); ^{13}C NMR (75 MHz, $CDCl_3$) δ 162.1, 161.1, 152.8, 151.0, 127.5, 126.2, 123.7, 123.4, 118.0, 116.5, 113.2, 71.1, 22.5, 19.0; IR (ATR) ν 2978 (w), 2929 (w), 1723 (s), 1594 (s), 1494 (m), 1426 (m), 1382 (s), 1270 (s), 1220 (m), 1173 (m), 1069 (s); HRMS (EI) calcd for $C_{14}H_{12}O_3$ [M^+] 228.0786, found 228.0782.

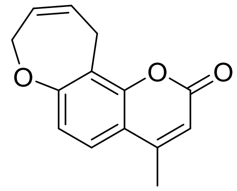

11d

2*H*-Furo[2,3-h]chromen-2-one (angelicin, 3a)[8,9]. Starting from **10a** (242 mg, 1.00 mmol) compound **3a** (177 mg, 0.95 mmol, 95%) was obtained. Colourless solid, mp 137 – 139 °C (literature: 139 °C[8]); ^{1}H NMR (300 MHz, $CDCl_3$) δ 7.78 (d, *J* = 9.6 Hz, 1H), 7.67 (d, *J* = 2.2 Hz, 1H), 7.40 (dd, *J* = 8.5, 0.8 Hz, 1H), 7.34 (d, *J* = 8.5 Hz, 1H), 7.09 (dd, *J* = 2.2, 0.8 Hz, 1H), 6.36 (d, *J* = 9.6 Hz, 1H); ^{13}C

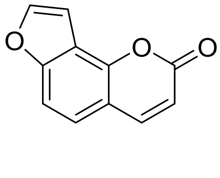

3a

NMR (75 MHz, $CDCl_3$) δ 160.8, 157.4, 148.5, 145.9, 144.6, 123.9, 116.9, 114.1, 113.6, 108.8, 104.1; IR (ATR) ν 3164 (w), 3082 (w), 1711 (s), 1615 (s), 1535 (m), 1442 (m), 1402 (m), 1336 (m), 1270 (s), 1114 (s), 1055 (s); HRMS (EI) calcd for $C_{11}H_6O_3$ [M^+] 186.0317, found 186.0312.

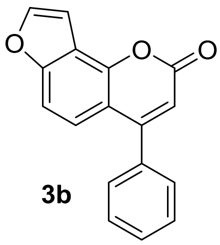

3b

4-Phenyl-2*H*-furo[2,3-*h*]chromen-2-one (3b).[10] Starting from **10b** (318 mg, 1.00 mmol) compound **3b** (233 mg, 0.89 mmol, 89%) was obtained. Colourless solid, mp 182 - 183 °C (literature: 165 – 166 °C[10]); ^{1}H NMR (300 MHz, $CDCl_3$) δ 7.70 (d, J = 2.2 Hz, 1H), 7.56 – 7.51 (m, 3H), 7.50 – 7.45 (m, 2H), 7.40 (d, J = 8.9 Hz, 1H), 7.36 (dd, J = 8.9, 0.8 Hz, 1H), 7.19 (dd, J = 2.2, 0.8 Hz, 1H), 6.34 (s, 1H); ^{13}C NMR (75 MHz, $CDCl_3$) δ 160.9, 157.5, 157.0, 148.8, 146.0, 136.0, 129.8, 129.0, 128.6, 123.2, 117.3, 113.8, 113.0, 108.5, 104.5; IR (ATR) ν 3122 (w), 3062 (w), 1713 (s), 1606 (s), 1569 (m), 1443 (m), 1370 (s), 1265 (m), 1243 (m), 1159 (m), 1064 (s); HRMS (EI) calcd for $C_{17}H_{10}O_3$ [M^+] 262.0630, found 262.0634.

6-Methoxy-2*H*-furo[2,3-*h*]chromen-2-one (sphondin, 3c).[9] Starting from **10c** (272 mg, 1.00 mmol) compound **3c** (212 mg, 0.98 mmol, 98%) was obtained. Colourless solid, mp 190 – 192 °C (literature: 190 – 192 °C[11]); ^{1}H NMR (300 MHz, $CDCl_3$) δ 7.74 (d, J = 9.6 Hz, 1H), 7.70 (d, J = 2.1 Hz, 1H), 7.12 (d, J = 2.1 Hz, 1H), 6.77 (s, 1H), 6.39 (d, J = 9.6 Hz, 1H), 4.04 (s, 3H); ^{13}C NMR (75 MHz, $CDCl_3$) δ 161.1, 147.1, 146.1, 144.5, 143.3, 143.2, 118.7, 114.6, 113.7, 104.7, 103.9, 56.7; IR (ATR) ν 3115 (w), 3066 (w), 1732 (s), 1582 (m), 1395 (m), 1345 (m), 1308 (m), 1336 (m), 1192 (m), 1172 (m), 1040 (m); HRMS (EI) calcd for $C_{12}H_8O_4$ [M^+] 216.0423, found 216.0420.

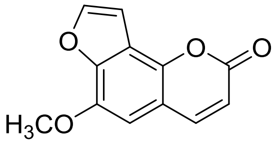

3c

4-Methyl-2*H*-furo[2,3-*h*]chromen-2-one (3d).[10] Starting from **10d** (256 mg, 1.00 mmol) compound **3d** (200 mg, 1.00 mmol, quant.) was obtained. Colourless solid, mp 190 - 192 °C (literature: 190 – 192 °C[12]); ^{1}H NMR (300 MHz, $CDCl_3$) δ 7.66 (d, *J* = 2.2 Hz, 1H), 7.49 (d, *J* = 8.8 Hz, 1H), 7.42 (d, *J* = 8.8 Hz, 1H), 7.11 (d, *J* = 2.1 Hz, 1H), 6.24 (s, 1H), 2.48 (d, *J* = 0.9 Hz, 3H); ^{13}C NMR (75 MHz, $CDCl_3$) δ 160.9, 157.3, 153.7, 148.0, 145.9, 120.6, 117.0, 114.6, 112.9, 108.5, 104.4, 19.5; IR (ATR) ν 3135 (w), 3108 (w), 1704 (s), 1614 (s), 1529 (m), 1437 (m), 1373 (s), 1262 (s), 1143 (m), 1063 (s), 1035 (m); HRMS (EI) calcd for $C_{12}H_8O_3$ [M^+] 200.0473, found 200.0476.

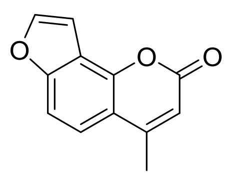

3d

General procedure for the synthesis of acrylates 12. The appropriate 8-allylcoumarin **8** (1.00 mmol) was dissolved in DMF (5.0 mL) and NEt_3 (180 μL, 1.30 mmol) was added. The solution was cooled to 0 °C and a solution of acryloyl chloride (117 μL, 1.30 mmol) in DMF (1.0 mL) was added dropwise. The mixture was stirred for 12 h at ambient temperature and the reaction was then quenched by addition of aq. NH_4Cl (5 mL). Ethyl acetate (10 mL) was added, the organic layer was separated and the aqueous layer was extracted twice with ethyl acetate (10 mL each). The combined organic layers were dried with $MgSO_4$, filtered and evaporated. The residue was purified by column chromatography on silica using hexanes-ethyl acetate mixtures as eluents to furnish the acrylates **12**.

8-Allyl-2-oxo-2*H*-chromen-7-ylacrylate (12a). Starting from **8a** (202 mg, 1.00 mmol) compound **12a** (236 mg, 0.92 mmol, 92%) was obtained. Colourless solid, mp 74 - 76 °C; ^{1}H NMR (300 MHz, $CDCl_3$) δ 7.69 (d, *J* = 9.6 Hz, 1H), 7.39 (d, *J* = 8.5 Hz, 1H), 7.08 (d, *J* = 8.5 Hz, 1H), 6.64 (dd, *J* = 17.3, 1.2 Hz, 1H), 6.39 (d, *J* = 9.6 Hz, 1H), 6.34 (dd, *J* = 17.3, 10.5 Hz, 1H), 6.08 (dd, *J* = 10.4, 1.2 Hz, 1H), 5.88 (ddt, *J* = 16.4, 10.0, 6.3 Hz, 1H), 5.04 (dm, *J* = 17.0 Hz, 1H), 5.01 (dm, *J* = 10.0 Hz, 1H), 3.55 (d, *J* = 6.3 Hz, 2H); ^{13}C NMR (75 MHz,

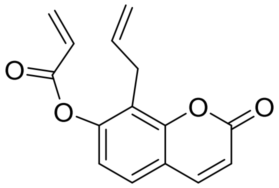

12a

$CDCl_3$) δ 164.0, 160.4, 153.0, 151.7, 143.4, 134.1, 133.6, 127.4, 126.3, 121.2, 119.2, 117.0, 116.5, 116.0, 27.9; IR (ATR) ν 3080 (w), 1728 (s), 1602 (s), 1400 (s), 1292 (m), 1230 (s), 1138 (s), 1104 (s), 1052 (m), 1019 (m); HRMS (EI) calcd for $C_{15}H_{12}O_4$ [M^+] 256.0736, found 256.0725.

8-Allyl-2-oxo-4-phenyl-2*H*-chromen-7-ylacrylate (12b). Starting from **8b** (278 mg, 1.00 mmol) compound **12b** (309 mg, 0.93 mmol, 93%) was obtained. Colourless oil; ^{1}H NMR (300 MHz, $CDCl_3$) δ 7.54 – 7.48 (m, 3H), 7.46 – 7.41 (m, 2H), 7.39 (d, J = 8.8 Hz, 1H), 7.02 (d, J = 8.8 Hz, 1H), 6.65 (d, J = 17.3 Hz, 1H), 6.35 (dd, J = 17.3, 10.5 Hz, 1H), 6.34 (s, 1H), 6.08 (d, J = 10.5 Hz, 1H), 5.92 (ddt, J = 16.8, 10.2, 6.6 Hz, 1H), 5.13 – 4.95 (m, 2H), 3.61 (d, J = 6.3 Hz, 2H); ^{13}C NMR (75 MHz, $CDCl_3$) δ 163.9, 160.4, 155.8, 153.0, 151.6, 135.4, 134.2, 133.6, 129.8, 129.0, 128.5, 127.3, 125.6, 121.3, 118.7, 117.2, 116.4, 114.5, 28.2; IR (ATR) ν 2925 (w), 1724 (s), 1597 (m), 1402 (m), 1370 (m), 1233 (m), 1134 (s), 982 (m), 907(m); HRMS (EI) calcd for $C_{21}H_{16}O_4$ [M^+] 332.1049, found 332.1040.

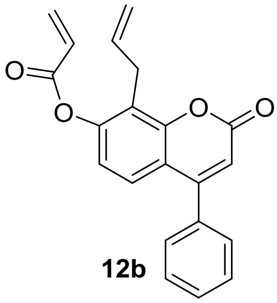

12b

8-Allyl-6-methoxy-2-oxo-2*H*-chromen-7-ylacrylate (12c). Starting from **8c** (232 mg, 1.00 mmol) compound **12c** (255 mg, 0.89 mmol, 89%) was obtained. Yellowish oil; ^{1}H-NMR (300 MHz, $CDCl_3$) δ 7.64 (d, J = 9.5 Hz, 1H), 6.87 (s, 1H), 6.64 (dd, J = 17.3, 1.2 Hz, 1H), 6.39 (d, J = 9.9 Hz, 1H), 6.36 (dd, J = 17.3, 10.3 Hz, 1H), 6.07 (dd, J = 10.4, 1.3 Hz, 1H), 5.86 (ddt, J = 16.5, 10.0, 6.4 Hz, 1H), 5.06 (dm, J = 17.1 Hz, 1H), 5.01 (dm, J = 10.0 Hz, 1H), 3.84 (s, 3H), 3.54 (d, J = 6.4 Hz, 2H); ^{13}C-NMR (75 MHz, $CDCl_3$) δ 163.3, 160.7, 148.6, 146.9, 143.4, 141.7, 134.0, 133.4, 127.1, 122.9, 116.8, 116.7, 116.4, 107.5, 56.5, 28.2; IR (ATR) ν 3091 (w), 2941 (w), 1719 (s), 1636 (m), 1575 (m), 1464 (m), 1400 (s), 1351 (m),

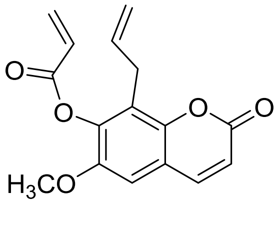

12c

1290 (s), 1240 (m), 1131 (s), 1102 (s); HRMS (EI) calcd for $C_{16}H_{14}O_5$ [M^+] 286.0841, found 286.0848.

8-Allyl-4-methyl-2-oxo-2*H*-chromen-7-ylacrylate (12d). Starting from **8d** (216 mg, 1.00 mmol) compound **12d** (232 mg, 0.86 mmol, 86%) was obtained. Colourless solid, mp 67 - 69 °C; ^{1}H NMR (300 MHz, $CDCl_3$) δ 7.52 (d, J = 8.7 Hz, 1H), 7.10 (d, J = 8.7 Hz, 1H), 6.65 (dd, J = 17.3, 1.1 Hz, 1H), 6.35 (dd, J = 17.3, 10.5 Hz, 1H), 6.27 (q, J = 1.0 Hz, 1H), 6.09 (dd, J = 10.4, 1.1 Hz, 1H), 5.88 (ddt, J = 16.4, 10.0, 6.3 Hz, 1H), 5.03 (dm, J = 17.0 Hz, 1H), 5.00 (dm, J = 10.0 Hz, 1H), 3.56 (d, J = 6.3 Hz, 2H), 2.43 (d, J = 1.1 Hz, 3H); ^{13}C NMR (75 MHz, $CDCl_3$) δ 164.0, 160.5, 152.5, 152.4, 151.5, 134.3, 133.6, 127.4, 123.0, 121.2, 118.8, 118.1, 116.4, 114.5, 28.1, 19.0; IR (ATR) ν 3082 (w), 2982 (w), 1723 (s), 1631 (m), 1599 (s), 1402 (m), 1383 (m), 1233 (m), 1137 (s), 1046 (s); HRMS (EI) calcd for $C_{16}H_{14}O_4$ [M^+] 270.0892, found 270.0883.

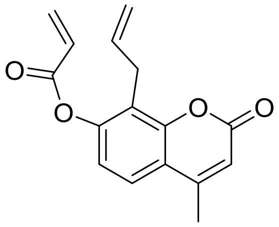

12d

General procedure for the synthesis of oxepin-2-one coumarins 13. The appropriate acrylate **12** (1.00 mmol) was dissolved in toluene (100 mL) and heated to 110 °C. At this temperature precatalyst **A** (42 mg, 0.05 mmol, 5 mol %) was added and the solution was stirred at 110 °C for 16 h. After cooling to ambient temperature, the solution was evaporated and the residue was purified by column chromatography on silica, using hexanes-ethyl acetate mixtures of increasing polarity as eluents, to furnish compounds **13**.

2*H*-Oxepino[2,3-*h*]chromen-2,8(11*H*)-dione (13a). Starting from **12a** (256 mg, 1.00 mmol) compound **13a** (185 mg, 0.81 mmol, 81%) was obtained. Colourless solid, mp 211 - 213 °C; ^{1}H NMR (300 MHz, $CDCl_3$) δ 7.75 (d, J = 9.6 Hz, 1H), 7.51 (d, J = 8.6 Hz, 1H), 7.30 (d, J = 9.6 Hz, 1H), 7.19 (d, J = 8.6 Hz, 1H), 6.45 (d, J = 9.6 Hz, 1H), 6.24 (dt, J = 9.8, 6.7 Hz, 1H), 3.15 (d, J = 6.7 Hz, 2H); ^{13}C NMR (75 MHz, $CDCl_3$) δ 167.0, 159.9, 152.1, 151.9, 143.1,

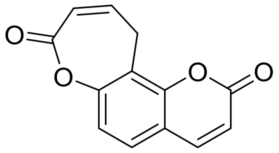

13a

128.0, 124.6, 124.2, 117.6, 116.6, 116.1, 115.5, 34.6; IR (ATR) ν 3082 (w), 2925 (w), 1768 (s), 1723 (s), 1597 (s), 1399 (m), 1238 (m), 1204 (m), 1108 (s), 1066 (s); HRMS (EI) calcd for $C_{13}H_{18}O_4$ [M^+] 228.0423, found 228.0431.

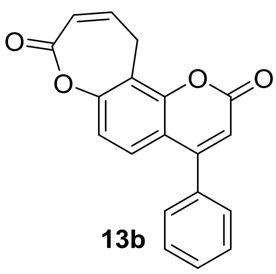

13b

4-Phenyl-2*H*-oxepino[2,3-*h*]chromen-2,8(11*H*)-dione (13b). Starting from **12b** (332 mg, 1.00 mmol) compound **13b** (240 mg, 0.79 mmol, 79%) was obtained. Colourless solid, mp 194 – 196 °C; ^{1}H NMR (300 MHz, $CDCl_3$) δ 7.57 – 7.52 (m, 3H), 7.50 (d, J = 8.9 Hz, 1H), 7.46 – 7.41 (m, 2H), 7.35 (d, J = 9.8 Hz, 1H), 7.12 (d, J = 8.9 Hz, 1H), 6.38 (s, 1H), 6.24 (dt, J = 9.8, 6.7 Hz, 1H), 3.15 (d, J = 6.7 Hz, 2H); ^{13}C NMR (75 MHz, $CDCl_3$) δ 167.1, 160.0, 155.6, 152.1, 152.0, 135.1, 130.1, 129.2, 128.5, 127.3, 124.6, 124.5, 117.2, 116.8, 115.8, 114.5, 34.7; IR (ATR) ν 3059 (w), 2923 (w), 1773 (s), 1718 (s), 1586 (s), 1445 (m), 1419 (m), 1370 (s), 1233 (m), 1208 (m), 1117 (m), 1068 (s), 1031 (m); HRMS (EI) calcd for $C_{19}H_{12}O_4$ [M^+] 304.0736, found 304.0732.

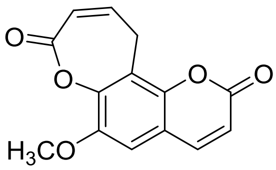

13c

6-Methoxy-2*H*-oxepino[2,3-*h*]chromen-2,8(11*H*)-dione (13c). Starting from **12c** (286 mg, 1.00 mmol) compound **13c** (235 mg, 0.91 mmol, 91%) was obtained. Colourless solid, mp 245 - 247 °C; ^{1}H NMR (300 MHz, $CDCl_3$) δ 7.68 (d, J = 9.5 Hz, 1H), 7.28 (d, J = 9.5 Hz, 1H), 6.96 (s, 1H), 6.44 (d, J = 9.5 Hz, 1H), 6.26 (dt, J = 9.7, 6.7 Hz, 1H), 3.95 (s, 3H), 3.13 (d, 6.7 Hz, 2H); ^{13}C NMR (75 MHz, $CDCl_3$) δ 167.0, 160.2, 147.7, 145.5, 143.1, 142.1, 125.3, 124.3, 117.9, 116.6, 114.9, 108.3, 56.7, 34.9; IR (ATR) ν 2918 (w), 2854 (w), 1726 (s), 1568 (m), 1456 (m), 1408 (m), 1281 (s), 1123 (s), 1074 (s); HRMS (EI) calcd for $C_{14}H_{10}O_5$ [M^+] 258.0528, found 258.0533.

4-Methyl-2*H*-oxepino[2,3-*h*]chromen-2,8(11*H*)-dione (13d). Starting from **12d** (270 mg, 1.00 mmol) compound **13d** (208 mg, 0.86 mmol, 86%) was obtained.

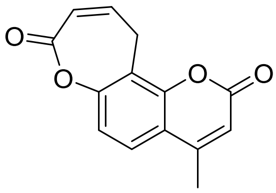

13d

Colourless solid, mp 213 - 215 °C; ^{1}H NMR (300 MHz, $CDCl_3$) δ 7.61 (d, J = 8.8 Hz, 1H), 7.29 (d, J = 9.8 Hz, 1H), 7.19 (d, J = 8.8 Hz, 1H), 6.30 (s, 1H), 6.21 (dt, J = 9.6, 6.8 Hz, 1H), 3.12 (d, J = 6.7 Hz, 2H), 2.46 (s, 3H); ^{13}C NMR (75 MHz, $CDCl_3$) δ 167.2, 160.0, 152.2, 152.0, 151.3, 124.8, 124.6, 124.4, 117.3, 116.7, 116.6, 114.5, 34.6, 19.0; IR (ATR) ν 3082 (w), 2926 (w), 1764 (s), 1732 (s), 1593 (s), 1383 (m), 1235 (m), 1206 (m), 1174 (m), 1065 (s); HRMS (EI) calcd for $C_{14}H_{10}O_4$ [M^+] 242.0579, found 242.0575.

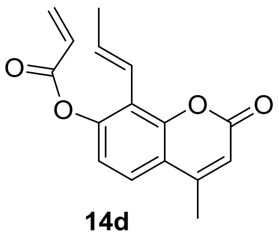

14d

(*E*)-4-Methyl-2-oxo-8-(prop-1-en-1-yl)-2*H*-chromen-7-ylacrylate (14d). Compound **12d** (270 mg, 1.00 mmol) was dissolved in toluene (10 mL) and [RuClH(CO)(PPh_3)$_3$] (77 mg, 0.08 mmol, 8 mol %) was added. The mixture was heated at 110 °C for 12 h, cooled to ambient temperature and evaporated. The residue was purified by column chromatography on silica using hexanes-MTBE mixtures as eluent to furnish the title compound **14d** (270 mg, 1.00 mmol, quant.) as a mixture of E/Z-isomers. Brownish oil; ^{1}H NMR (300 MHz, $CDCl_3$) δ 7.46 (d, J = 8.7 Hz, 1H), 7.05 (d, J = 8.7 Hz, 1H), 6.65 (d, J = 17.2 Hz, 1H), 6.59 – 6.49 (m, 1H), 6.35 (dd, J = 17.3, 10.4 Hz, 1H), 6.27 (s, 1H), 6.08 (d, J = 10.5 Hz, 1H), 2.43 (s, 3H), 1.91 (d, J = 5.5 Hz, 3H); ^{13}C NMR (75 MHz, $CDCl_3$) δ 164.0, 160.5, 152.4, 151.9, 150.4, 134.8, 134.3, 133.5, 127.5, 127.4, 122.7, 119.0, 118.2, 114.4, 20.0, 19.1.

General procedure for the synthesis of 8-(prop-1-enyl)coumarins 16. To a solution of the corresponding MOM-protected 8-allylcoumarin **7** (1.00 mmol) in toluene (10 mL) was added [RuClH(CO)(PPh_3)$_3$] (48 mg, 0.05 mmol, 5 mol %). It was heated at 65 °C for 12 h, cooled to ambient temperature and evaporated. The residue was redissolved in methanol (20 mL) and aq. HCl (3 M, 100 μL) was added. The solution was heated at 65 °C for 1 h, cooled to ambient temperature and diluted with water (30 mL) and ethyl acetate (30 mL). The organic layer was separated and the aqueous layer was extracted twice with ethyl acetate (30 mL

each). The combined organic layers were dried with $MgSO_4$, filtered and evaporated. The residue was purified by column chromatography on silica using hexanes-ethyl acetate mixtures as eluent to furnish the compounds **16**.

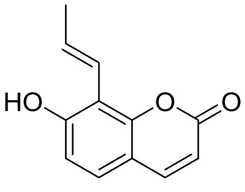

16a

(*E*)-7-Hydroxy-8-(prop-1-en-1-yl)-2*H*-chromen-2-one (16a). Starting from **7a** (246 mg, 1.00 mmol) compound **16a** (192 mg, 0.95 mmol, 95%) was obtained. Yellowish solid, mp 180 – 182 °C; ^{1}H NMR (300 MHz, acetone-d_6) δ 9.42 (s(br), 1H), 7.84 (d, J = 9.5 Hz, 1H), 7.34 (d, J = 8.5 Hz, 1H), 6.91 (d, J = 8.5 Hz, 1H), 6.84 (dq, J =16.1, 6.4 Hz, 1H), 6.73 (dq, J = 16.1, 1.4 Hz, 1H), 6.18 (d, J = 9.5 Hz, 1H), 1.94 (dd, J =6.3, 1.4 Hz, 3H); ^{13}C NMR (75 MHz, acetone-d_6) δ 161.0, 159.2, 153.9, 145.3, 132.7, 127.8, 120.6, 113.5, 113.2, 113.1, 112.6, 20.1; IR (ATR) ν 3314 (br), 2957 (w), 1690 (s), 1590 (s), 1558 (s), 1405 (m), 1298 (s), 1247 (s), 1107 (s), 1026 (m); HRMS (EI) calcd for $C_{12}H_{10}O_3$ [M^+] 202.0630, found 202.0633.

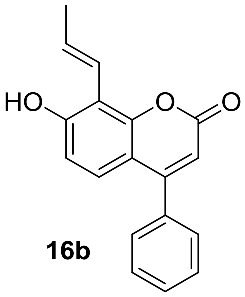

16b

(*E*)-7-Hydroxy-4-phenyl-8-(prop-1-en-1-yl)-2*H*-chromen-2-one (16b). Starting from **7b** (322 mg, 1.00 mmol) compound **16b** (225 mg, 0.81 mmol, 81%) was obtained. Colourless solid, mp 204 – 206 °C; ^{1}H NMR (300 MHz, DMSO-d_6) δ 10.80 (s, 1H), 7.56 – 7.45 (m, 5H), 7.10 (d, J = 8.8 Hz, 1H), 6.86 (d, J = 8.8 Hz, 1H), 6.79 (dq, J = 16.1, 6.3 Hz, 1H), 6.66 (dq, J = 16.1, 1.3 Hz, 1H), 6.14 (s, 1H), 1.93 (dd, J =6.3, 1.1 Hz, 3H); ^{13}C NMR (75 MHz, DMSO-d_6) δ 159.9, 158.8, 155.9, 152.4, 135.4, 131.3, 129.4, 128.7, 128.3, 125.4, 119.8, 112.6, 111.8, 110.8, 110.0, 19.8; IR (ATR) ν 3104 (br), 2955 (w), 1666 (s), 1593 (m), 1547 (s), 1371 (s), 1304 (m), 1282 (m), 1107 (m), 1071 (m); HRMS (EI) calcd for $C_{18}H_{14}O_3$ [M^+] 278.0943, found 278.0941.

(*E*)-7-Hydroxy-6-methoxy-8-(prop-1-en-1-yl)-2*H*-chromen-2-one (16c). Starting from **7c** (276 mg, 1.00 mmol) compound **16c** (232 mg, 1.00 mmol, quant.) was obtained. Yellowish solid, mp 152 – 154 °C; ^{1}H NMR (300 MHz, DMSO-d_6) δ 9.92 (s, 1H), 7.90 (d, *J* = 9.4 Hz, 1H), 7.13 (s, 1H), 6.77 (dq, *J* = 16.1, 6.5 Hz, 1H), 6.61 (dq, *J* = 16.1, 1.4 Hz, 1H), 6.24 (d, *J* = 9.4 Hz, 1H), 3.86 (s, 3H), 1.92 (dd, *J* = 6.2, 1.2 Hz, 3H); ^{13}C NMR (75 MHz, DMSO-d_6) δ 160.4, 148.2, 147.0, 144.9, 144.5, 131.5, 119.8, 111.9, 111.5, 110.3, 107.2, 56.2, 19.7; IR (ATR) ν 3347 (br), 2943 (w), 1702 (s), 1566 (s), 1484 (m), 1418 (m), 1288 (s), 1152 (m), 1102 (m); HRMS (EI) calcd for $C_{13}H_{12}O_4$ [M$^+$] 232.0736, found 232.0746.

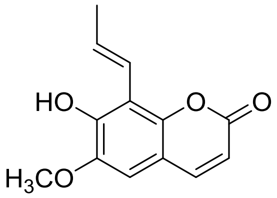

16c

(*E*)-7-Hydroxy-4-methyl-8-(prop-1-en-1-yl)-2*H*-chromen-2-one (16d)[5]. Starting from **7d** (260 mg, 1.00 mmol) compound **16d** (199 mg, 0.92 mmol, 92%) was obtained. Colourless solid, mp 216 – 218 °C (no mp or other characterization data published in the literature); ^{1}H NMR (300 MHz, DMSO-d_6) δ 10.68 (s, 1H), 7.43 (d, *J* = 8.7 Hz, 1H), 6.88 (d, *J* = 8.7 Hz, 1H), 6.75 (dq, *J* = 16.3, 6.3 Hz, 1H), 6.61 (d, *J* = 16.2 Hz, 1H), 6.13 (s, 1H), 2.35 (s, 3H), 1.91 (d, *J* =6.3 Hz, 3H); ^{13}C NMR (75 MHz, DMSO-d_6) δ 160.1, 158.6, 153.9, 151.8, 131.2, 123.8, 120.0, 112.4, 112.1, 111.3, 110.0, 19.9, 18.4; IR (ATR) ν 3269 (br), 2955 (w), 1676 (s), 1600 (m), 1566 (s), 1443 (m), 1389 (m), 1367 (m), 1282 (m), 1058 (m); HRMS (EI) calcd for $C_{13}H_{12}O_3$ [M$^+$] 216.0786, found 216.0797.

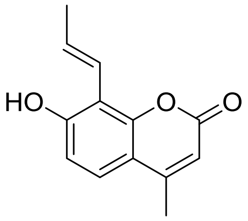

16d

General procedure for the synthesis of allyl ethers 17. The corresponding coumarin **16** (1.00 mmol) was dissolved in acetone (5 mL) and K_2CO_3 (280 mg, 2.00 mmol) and allyl bromide (128 μL, 1.50 mmol) were added. The mixture was heated at 50 °C for 16 h, cooled to ambient temperature and brine (20 mL) and ethyl acetate (30 mL) were added. The organic layer was separated and the aqueous layer was extracted twice with ethyl acetate (30 mL each). The combined organic extracts were dried with $MgSO_4$, filtered and evaporated. The

residue was purified by column chromatography on silica, using hexanes-MTBE mixtures as eluent, to furnish compounds **17**.

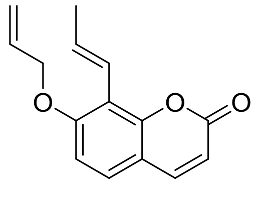

17a

(*E*)-7-(Allyloxy)-8-(prop-1-en-1-yl)-2*H*-chromen-2-one (17a). Starting from **16a** (202 mg, 1.00 mmol) compound **17a** (225 mg, 0.93 mmol, 93%) was obtained. Yellowish oil; ^{1}H NMR (300 MHz, $CDCl_3$) δ 7.60 (d, J = 9.5 Hz, 1H), 7.22 (d, J = 8.6 Hz, 1H), 6.87 (dq, J = 16.2, 6.3 Hz, 1H), 6.82 (d, J = 8.6 Hz, 1H), 6.73 (dq, J = 16.2, 1.3 Hz, 1H), 6.23 (d, J = 9.4 Hz, 1H), 6.07 (ddt, J = 17.2, 10.5, 5.1 Hz, 1H), 5.43 (dm, J = 17.3 Hz, 1H), 5.32 (dm, J = 10.5 Hz, 1H), 4.65 (dm, J = 5.0 Hz, 2H), 1.97 (dd, J = 6.2, 1.3 Hz, 3H); ^{13}C NMR (75 MHz, $CDCl_3$) δ 161.2, 159.0, 152.5, 144.0, 133.7, 132.6, 126.3, 119.2, 118.2, 115.1, 113.2, 113.1, 109.0, 69.8, 20.3; IR (ATR) ν 2912 (w), 1716 (s), 1594 (s), 1557 (m), 1490 (m), 1404 (m), 1272 (s), 1244 (s), 1114 (s); HRMS (EI) calcd for $C_{15}H_{14}O_3$ [M^+] 242.0943, found 242.0937.

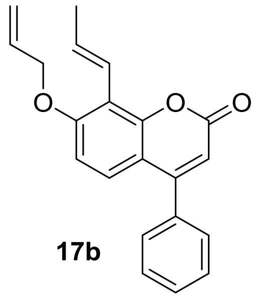

17b

(*E*)-7-(Allyloxy)-4-phenyl-8-(prop-1-en-1-yl)-2*H*-chromen-2-one (17b). Starting from **16b** (278 mg, 1.00 mmol) compound **17b** (283 mg, 0.89 mmol, 89%) was obtained. Yellowish oil; ^{1}H NMR (300 MHz, $CDCl_3$) δ 7.52 – 7.48 (m, 3H), 7.44 – 7.39 (m, 2H), 7.23 (d, J = 8.9 Hz, 1H), 6.95 – 6.74 (m, 3H), 6.21 (s, 1H), 6.07 (ddt, J = 17.2, 10.4, 5.1 Hz, 1H), 5.43 (dm, J = 17.3 Hz, 1H), 5.32 (dm, J = 10.6 Hz, 1H), 4.65 (dm, J = 5.1 Hz, 2H), 1.99 (d, J = 5.9 Hz, 3H); ^{13}C NMR (75 MHz, $CDCl_3$) δ 161.2, 159.0, 156.3, 152.6, 136.0, 133.7, 132.7, 129.6, 128.8, 128.5, 125.6, 119.5, 118.1, 117.1, 113.3, 112.1, 108.7, 69.8, 20.3; IR (ATR) ν 3070 (w), 2911 (w), 1714 (s), 1588 (s), 1552 (m), 1445 (m), 1370 (s), 1273 (s), 1112 (s), 1074 (s); HRMS (EI) calcd for $C_{21}H_{18}O_3$ [M^+] 318.1256, found 318.1246.

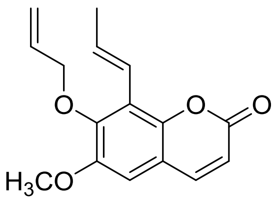

17c

(*E*)-7-(Allyloxy)-6-methoxy-8-(prop-1-en-1-yl)-2*H*-chromen-2-one (17c). Starting from **16c** (232 mg, 1.00 mmol) compound **17c** (245 mg, 0.90 mmol, 90%) was obtained. Yellowish oil; ^{1}H NMR (300 MHz, $CDCl_3$) δ 7.59 (d, J = 9.5 Hz, 1H), 6.89 (dq, J = 16.2, 6.6 Hz, 1H), 6.75 (s, 1H), 6.65 (dq, J = 16.2, 1.6 Hz, 1H), 6.31 (d, J = 9.5 Hz, 1H), 6.06 (ddt, J = 17.1, 10.4, 5.9 Hz, 1H), 5.35 (dm, J = 17.1 Hz, 1H), 5.23 (dm, J = 10.5 Hz, 1H), 4.52 (d, J = 5.9 Hz, 2H), 3.87 (s, 3H), 1.95 (d, J = 6.6, 1.6 Hz, 3H); ^{13}C NMR (75 MHz, $CDCl_3$) δ 161.1, 150.1, 149.1, 147.0, 143.8, 134.6, 133.8, 121.2, 119.7, 118.1, 114.9, 114.8, 107.4, 74.2, 56.3, 20.1; IR (ATR) ν 2936 (w), 1715 (s), 1597 (m), 1558 (s), 1460 (m), 1400 (m), 1288 (s), 1142 (s), 1102 (s). HRMS (EI) calcd for $C_{16}H_{16}O_4$ [M^+] 272.1049, found 272.1046.

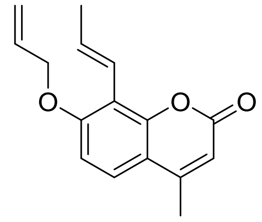

17d

(*E*)-7-(Allyloxy)-4-methyl-8-(prop-1-en-1-yl)-2*H*-chromen-2-one (17d)[13]. Starting from **16d** (216 mg, 1.00 mmol) compound **17d** (256 mg, 1.00 mmol, quant.) was obtained. Yellowish oil; ^{1}H NMR (300 MHz, $CDCl_3$) δ 7.35 (d, J = 8.9 Hz, 1H), 6.82 (dq, J = 16.2, 6.0 Hz, 1H), 6.82 (d, J = 8.8 Hz, 1H), 6.73 (dm, J = 16.3 Hz, 1H), 6.12 (s, 1H), 6.07 (ddt, J = 17.2, 10.6, 5.2 Hz, 1H), 5.43 (dm, J = 17.3 Hz, 1H), 5.32 (dm, J = 10.5 Hz, 1H), 4.65 (dm, J = 5.0 Hz, 2H), 2.37 (d, J = 1.1 Hz, 3H), 1.96 (d, J = 5.0 Hz, 3H); ^{13}C NMR (75 MHz, $CDCl_3$) δ 161.2, 158.8, 152.8, 151.9, 133.5, 132.7, 122.9, 119.5, 118.1, 114.9, 114.1, 112.1, 108.6, 69.7, 20.3, 18.9; IR (ATR) ν 2917 (w), 1724 (s), 1593 (s), 1384 (m), 1277 (m), 1112 (m), 1071 (m); HRMS (EI) calcd for $C_{16}H_{16}O_3$ [M^+] 256.1099, found 256.1093.

General procedure for the synthesis of pyran-2-one coumarins 15. A solution of the corresponding diene **17** (1.00 mmol) in benzene (10 mL) was heated to 40 °C and precatalyst **B** (42 mg, 0.05 mmol, 5 mol %) was added. The solution was heated at 40 °C until the starting material was fully consumed (TLC, ca 1 h). A solution of *tert*-BuOOH in decane (5.5

M, 364 μL, 4.00 mmol) was then slowly added via syringe and stirring at 40 °C was continued for 1 h. After cooling to ambient temperature the reaction mixture was diluted with ethyl acetate (30 mL), dried with $MgSO_4$, filtered and evaporated. The residue was purified by column chromatography on silica, using hexanes-ethyl acetate mixtures of increasing polarity as eluent, to furnish compounds **15**.

2*H*,8*H*-Pyrano[2,3-*f*]chromen-2,8-dione (15a)[14]. Starting from **17a** (242 mg, 1.00 mmol) compound **15a** (120 mg, 0.56 mmol, 56%) was obtained. Colourless solid, mp 200 – 203 °C (267 – 269 °C[14]); ^{1}H NMR (300 MHz, DMSO-d_6) δ 8.29 (d, J = 9.7 Hz, 1H), 8.13 (d, J = 9.6 Hz, 1H), 7.94 (d, J = 8.6 Hz, 1H), 7.39 (d, J = 8.8 Hz, 1H), 6.61 (d, J = 9.8 Hz, 1H), 6.54 (d, J = 9.6 Hz, 1H); ^{13}C NMR (75 MHz, DMSO-d_6) δ 159.0, 159.0, 155.4, 150.0, 144.1, 136.8, 131.3, 116.7, 115.0, 114.7, 112.9, 107.7; IR (ATR) ν 3078 (w), 1731 (s), 1623 (m), 1570 (m), 1405 (w), 1250 (w), 1167 (m), 1116 (m); HRMS (EI) calcd for $C_{12}H_6O_4$ [M^+] 214.0266, found 214.0261.

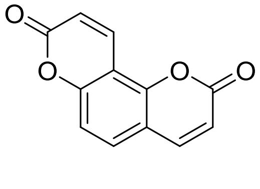

15a

4-Phenyl-2*H*,8*H*-pyrano[2,3-*f*]chromen-2,8-dione (15b)[15]. Starting from **17b** (318 mg, 1.00 mmol) compound **15b** (119 mg, 0.41 mmol, 41%) was obtained. Colourless solid, mp 216 – 218 °C (no mp or other data reported in the literature); ^{1}H NMR (300 MHz, $CDCl_3$) δ 8.38 (d, J = 9.8 Hz, 1H), 7.64 (d, J = 9.0 Hz, 1H), 7.58 – 7.53 (m, 3H), 7.47 – 7.42 (m, 2H), 7.19 (d, J = 9.0 Hz, 1H), 6.55 (d, J = 9.8 Hz, 1H), 6.39 (s, 1H); ^{13}C NMR (75 MHz, $CDCl_3$) δ 159.5, 159.4, 156.3, 155.8, 150.8, 137.0, 134.8, 130.3, 129.8, 129.3, 128.5, 117.2, 115.0, 114.1, 113.1, 108.8; IR (ATR) n 3068 (w), 2934 (w), 1721 (s), 1621 (s), 1565 (m), 1384 (m), 1279 (m), 1161 (m), 1104 (s); HRMS (EI) calcd for $C_{18}H_{10}O_4$ [M^+] 290.0579, found 290.0576.

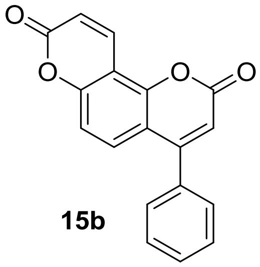

15b

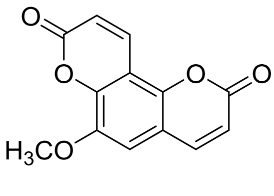

15c

6-Methoxy-2*H*,8*H*-pyrano[2,3-*f*]chromen-2,8-dione (15c) [4]. Starting from **17c** (272 mg, 1.00 mmol) compound **15c** (110 mg, 0.45 mmol, 45%) was obtained. Colourless solid, mp 269 – 271 °C (no mp reported in the literature [4]); ^{1}H NMR (300 MHz, $CDCl_3$) δ 8.28 (d, J = 9.8 Hz, 1H), 7.70 (d, J = 9.6 Hz, 1H), 7.07 (s, 1H), 6.55 (d, J = 9.8 Hz, 1H), 6.45 (d, J = 9.6 Hz, 1H), 4.00 (s, 3H); ^{13}C NMR (75 MHz, $CDCl_3$) δ 159.5, 158.7, 146.9, 144.9, 144.7, 143.0, 136.8, 117.6, 116.3, 114.0, 111.2, 109.5, 57.1; IR (ATR) ν 3120 (w), 1732 (s), 1622 (m), 1574 (m), 1469 (m), 1416 (m), 1285 (m), 1135 (m), 1075 (m); HRMS (EI) calcd for $C_{13}H_8O_5$ [M^+] 244.0372, found 244.0367.

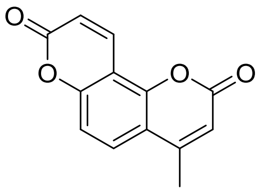

15d

4-Methyl-2*H*,8*H*-pyrano[2,3-*f*]chromen-2,8-dione (15d) [16]. Starting from **17d** (256 mg, 1.00 mmol) compound **15d** (107 mg, 0.47 mmol, 47%) was obtained. Colourless solid, mp 222 – 224 °C (269 – 272 °C[16]); ^{1}H NMR (300 MHz, $CDCl_3$) δ 8.32 (d, J = 9.8 Hz, 1H), 7.74 (d, J = 8.9 Hz, 1H), 7.26 (d, J = 8.9 Hz, 1H), 6.53 (d, J = 9.8 Hz, 1H), 6.32 (s, 1H), 2.48 (s, 3H); ^{13}C NMR (75 MHz, $CDCl_3$) δ 159.5, 156.2, 153.0, 152.5, 150.2, 137.1, 127.4, 121.5, 117.1, 115.9, 114.2, 113.2, 19.1; IR (ATR) ν 2972 (w), 2926 (w), 1720 (s), 1601 (s), 1387 (m), 1264 (m), 1184 (m), 1074 (s); HRMS (EI) calcd for $C_{13}H_8O_4$ [M^+] 228.0423, found 228.0425.

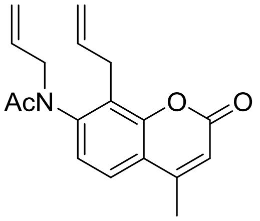

19

***N*-Allyl-*N*-(8-allyl-4-methyl-2-oxo-2*H*-chromen-7-yl)acetamide (19)**. To a solution of **18** (257 mg, 1.00 mmol) in acetone (5 mL) was added K_2CO_3 and allyl bromide (256 μL, 3.00 mmol). The mixture was heated to 50 °C for 16 h, cooled to ambient temperature and brine (20 mL) and ethyl acetate (30 mL) were added. The organic layer was separated and the aqueous layer was extracted twice with ethyl acetate (30 mL each). The combined organic extracts were dried with $MgSO_4$, filtered and evaporated. The residue was purified by column chromatography on

silica with hexanes-ethyl acetate (1 : 1 (v/v)) mixture as eluent to furnish compound **19** (322 mg, 1.00 mmol, quant.). Yellow solid, mp 102 - 104 °C; ^{1}H NMR (300 MHz, DMSO-d_6) δ 7.74 (d, J = 8.4 Hz, 1H), 7.25 (d, J = 8.4 Hz, 1H), 6.45 (s, 1H), 5.94 (ddt, J = 16.9, 10.2, 6.3 Hz, 1H), 5.82 (dddd, J = 17.3, 10.3, 7.4, 5.4 Hz, 1H), 5.10 – 4.94 (m, 4H), 4.72 (dd, J = 14.8, 5.3 Hz, 1H), 3.61 (dd, J = 14.8, 7.5 Hz, 1H), 3.46 (d, J = 6.1 Hz, 2H), 2.45 (s, 3H), 1.67 (s, 3H); ^{13}C NMR (75 MHz, DMSO-d_6) δ 169.3, 159.8, 153.6, 152.4, 144.1, 135.0, 133.6, 126.2, 125.8, 124.6, 119.9, 118.7, 117.0, 115.0, 51.3, 29.9, 22.9, 18.7; IR (ATR) ν 3079 (w), 2980 (w), 1730 (s), 1663 (s), 1599 (s), 1419 (m), 1386 (s), 1299 (m), 1254 (m); HRMS (EI) calcd for $C_{18}H_{19}O_3N$ [M$^+$] 297.1365, found 297.1354.

(*E*,*E*)-*N*-(4-Methyl-2-oxo-8-(prop-1-en-1-yl)-2*H*-chromen-7-yl)-*N*-(prop-1-en-1-yl)acetamide (20). To a solution of **19** (297 mg, 1.00 mmol) in toluene (10 mL) was added [RuClH(CO)(PPh$_3$)$_3$] (48 mg, 0.05 mmol, 5 mol %). The solution was heated to 65 °C for 12 h, cooled to ambient temperature and evaporated. The residue was purified by column chromatography on silica, using hexanes-ethyl acetate mixture (1 : 1 (v/v)) as eluent to furnish compound **20** (280 mg, 0.94 mmol, 94%) as a mixture of diastereoisomers. NMR data

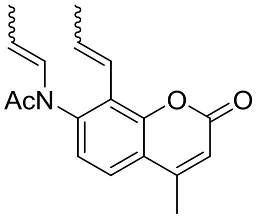

20

for the major isomer (*E*,*E*)-**20**: ^{1}H NMR (300 MHz, CDCl$_3$) δ 7.53 (d, J = 8.3 Hz, 1H), 7.40 (dm, J = 14.6 Hz, 1H), 7.06 (d, J = 8.3 Hz, 1H), 6.72 (dq, J = 16.1, 6.6 Hz, 1H), 6.35 (s, 1H), 6.29 (dm, J = 16.3 Hz, 1H), 4.38 (dq, J = 14.5, 6.9 Hz, 1H), 2.46 (s, 3H), 1.91 (d, J = 6.7 Hz, 3H), 1.76 (s, 3H), 1.62 (d, J = 6.9 Hz, 3H); ^{13}C NMR (75 MHz, CDCl$_3$) δ 167.9, 160.1, 152.2, 152.1, 140.2, 136.3, 128.0, 125.5, 125.4, 123.4, 120.4, 119.5, 115.6, 109.6, 23.0, 20.3, 19.2, 15.2.

7-Acetyl-4-methyl-8,11-dihydrochromeno[7,8-*b*]azepin-2(7*H*)-one (21). To a solution of **19** (297 mg, 1.00 mmol) in toluene (10 mL) was added precatalyst **A** (42 mg, 0.05 mmol, 5 mol %) and the solution was heated to 90 °C until the starting material was fully consumed

(TLC, ca 1 h). The mixture was evaporated and the residue was purifed by column chromatography on silica, using hexanes-ethyl acetate mixture (1 : 1 (v/v)) as eluent, to furnish compound **21** (266 mg, 0.99 mmol, 99%). Colourless solid, mp 147 - 150 °C; ^{1}H NMR (300 MHz, $CDCl_3$) δ 7.53 (d, J = 8.3 Hz, 1H), 7.15 (d, J = 8.3 Hz, 1H), 6.28 (s, 1H), 5.81 – 5.70 (m, 1H), 5.54 – 5.44 (m, 1H), 5.41 – 5.30 (m, 1H), 3.91 (dd, J = 16.3, 8.6 Hz, 1H), 3.44 – 3.30 (2H), 2.43 (s, 3H), 1.84 (s, 3H); ^{13}C NMR (75 MHz, $CDCl_3$) δ 169.4, 160.2, 152.2, 150.4, 145.5, 128.4, 127.3, 123.6, 123.5, 123.4, 119.8, 115.2, 44.7, 22.2, 22.1, 19.0; IR (ATR) ν 3026 (w), 2982 (w), 1726 (s), 1666 (s), 1599 (s), 1426 (m), 1381 (s), 1306 (m), 1234 (m); HRMS (EI) calcd for $C_{16}H_{15}O_3N$ [M^+] 269.1052, found 269.1056.

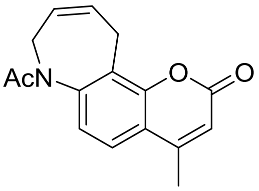

21

References

1. Schultze, C.; Schmidt, B. *J. Org. Chem.* **2018,** *83*, 5210-5224.
2. Nichols, D. B.; Leão, R. A. C.; Basu, A.; Chudayeu, M.; Moraes, P. d. F.; Talele, T. T.; Costa, P. R. R.; Kaushik-Basu, N. *Chem. Biol. Drug Design* **2013,** *81*, 607-614.
3. Ahuja, M.; Bandopad, M.; Seshadri, T. R. *Indian J. Chem.* **1974,** *12*, 292-294.
4. Nagaiah, K.; Krupadanam, G. L. D.; Srimannarayana, G. *Indian J. Chem.* **2002,** *41B*, 858-862.
5. Zak, J.; Ron, D.; Riva, E.; Harding, H. P.; Cross, B. C. S.; Baxendale, I. R. *Chem. Eur. J.* **2012,** *18*, 9901-9910.
6. Litinas, K. E.; Mangos, A.; Nikkou, T. E.; Hadjipavlou-Litina, D. J. *J. Enzyme Inhib. Med. Chem.* **2011,** *26*, 805-812.
7. Chattopadhyay, S. K.; Maity, S.; Panja, S. *Tetrahedron Lett.* **2002,** *43*, 7781-7783.
8. Tsai, T.-W.; Wang, E.-C. *J. Chin. Chem. Soc.* **2004,** *51*, 1019-1023.

9. Wulff, W. D.; McCallum, J. S.; Kunng, F. A. *J. Am. Chem. Soc.* **1988,** *110*, 7419-7434.

10. Kitamura, T.; Otsubo, K. *J. Org. Chem.* **2012,** *77*, 2978-2982.

11. Coassini Lokar, L. R.; Delben, S. *Phytochemistry* **1988,** *27*, 1073-1077.

12. Bhayani, R. J.; Thakor, V. M. *J. Indian Chem. Soc.* **1990,** *67*, 71-72.

13. Chattopadhyay, S. K.; Mondal, P.; Ghosh, D. *Synthesis* **2014,** *46*, 3331-3340.

14. Bethke, J.; Margaretha, P.; M. Wynne, A.; A. Caldwell, R. *J. Chem. Res. (S)* **1998**, 142-143.

15. Raju, M. S.; Subba Rao, N. V. *Proc. Indian Acad. Sci. A* **1974,** *79*, 223-229.

16. Maruyama, J.; Ito, K. *Chem. Pharm. Bull.* **1984,** *32*, 1178-1182.

^{1}H NMR (300 MHz, acetone-d_6) of **8b**

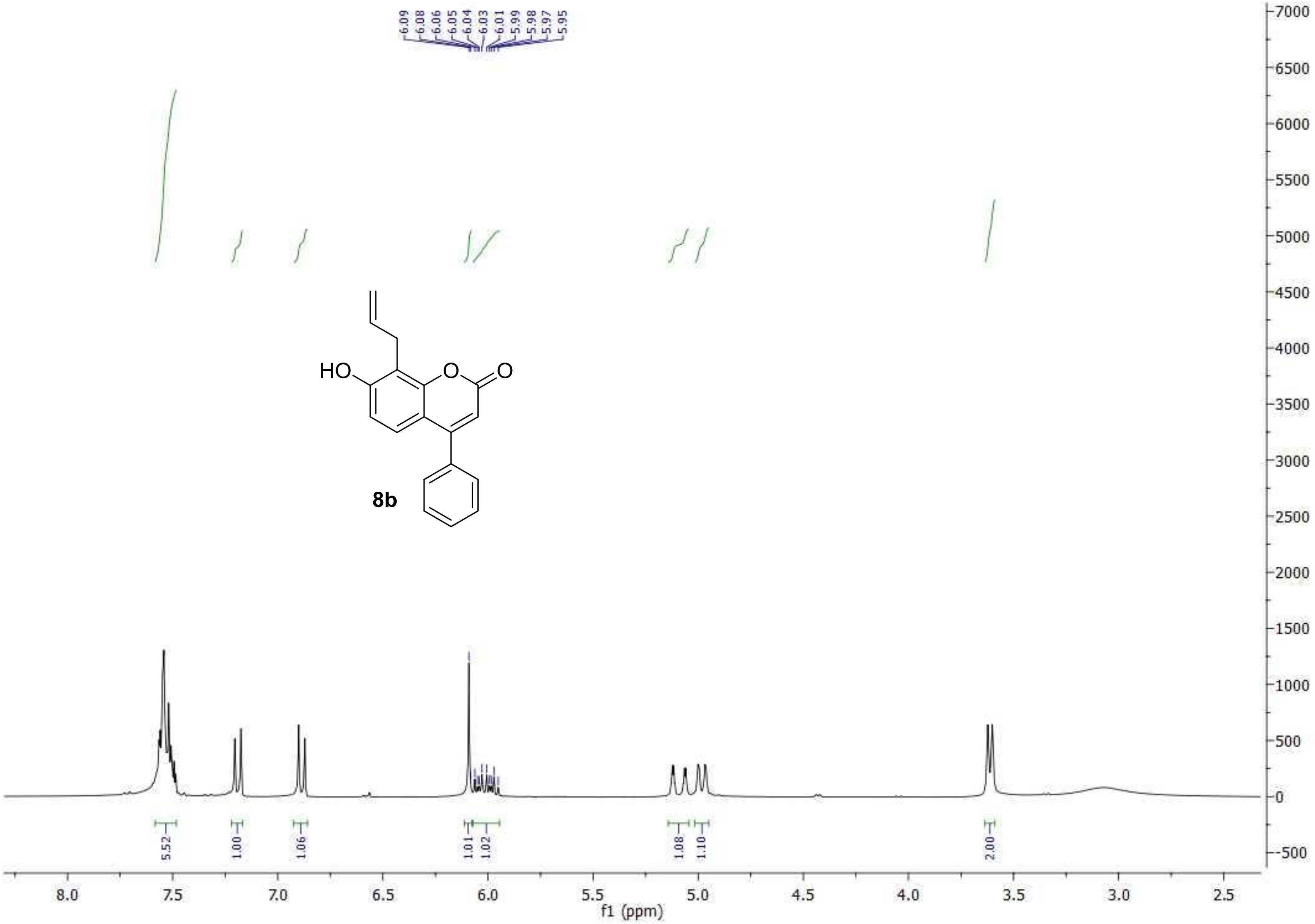

^{13}C NMR (75 MHz, acetone-d_6) of **8b**

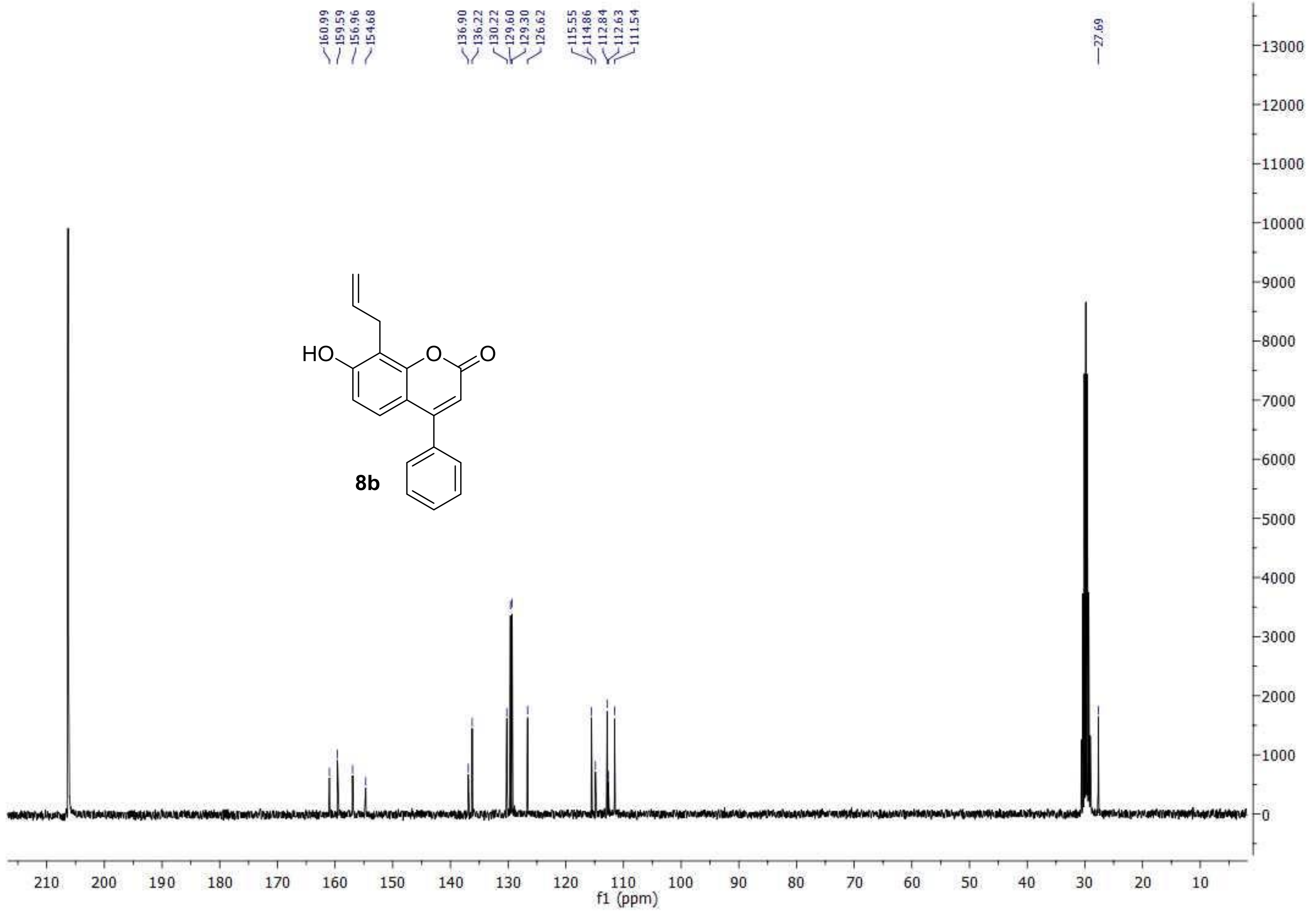

^{1}H NMR (300 MHz, $CDCl_3$) of **8c**

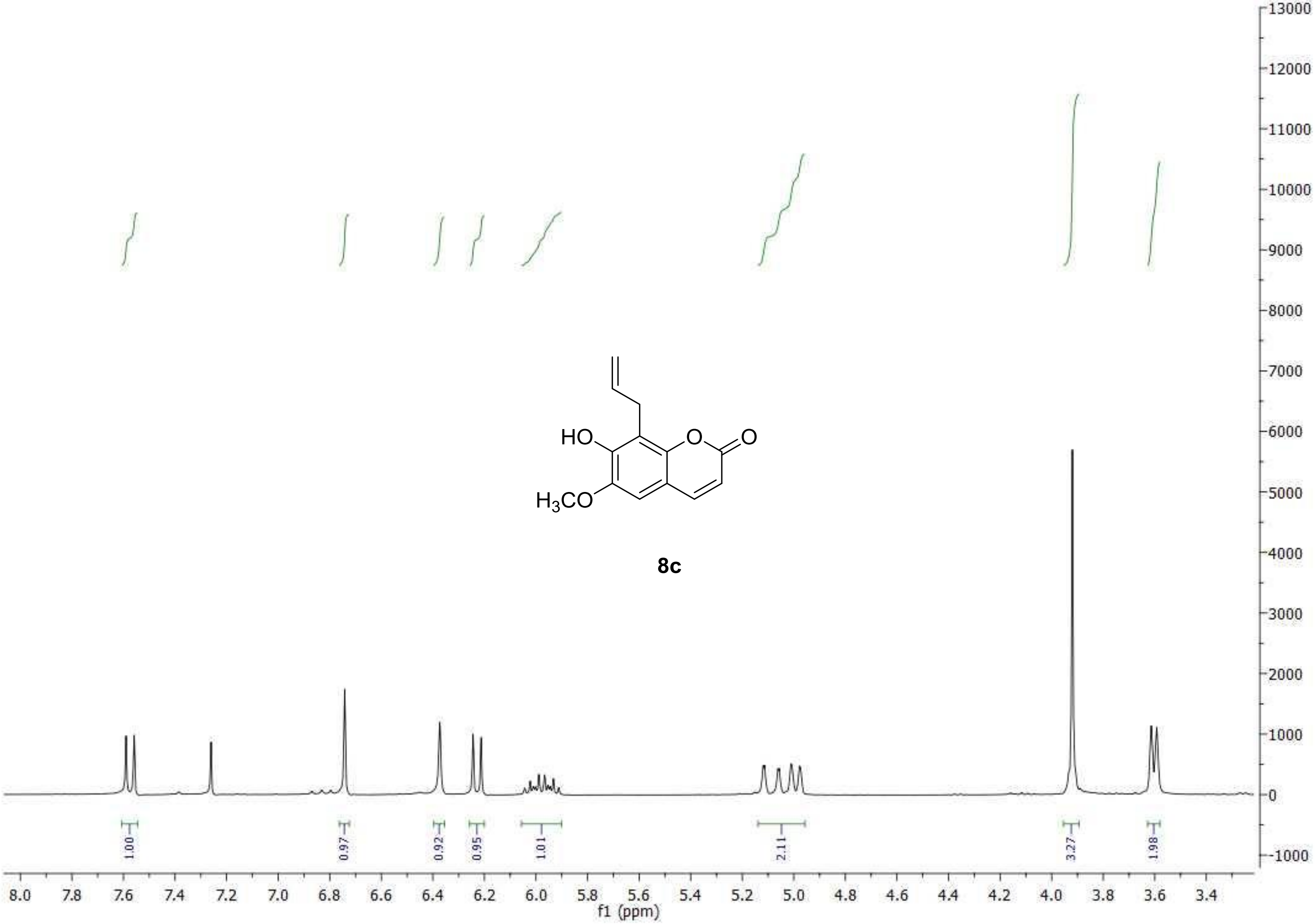

^{13}C NMR (75 MHz, $CDCl_3$) of **8c**

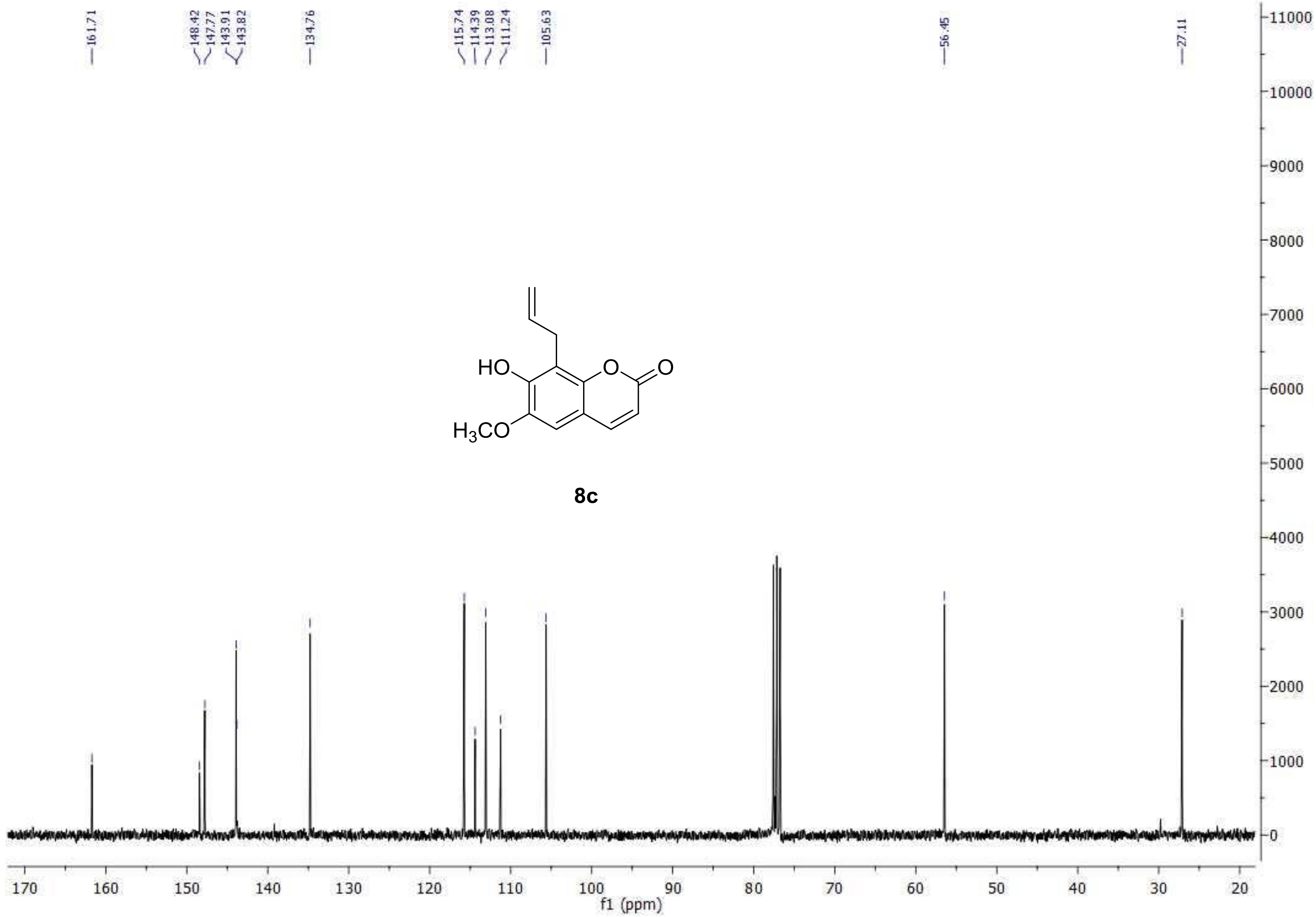

^{1}H NMR (300 MHz, DMSO-d_6) of **8d**

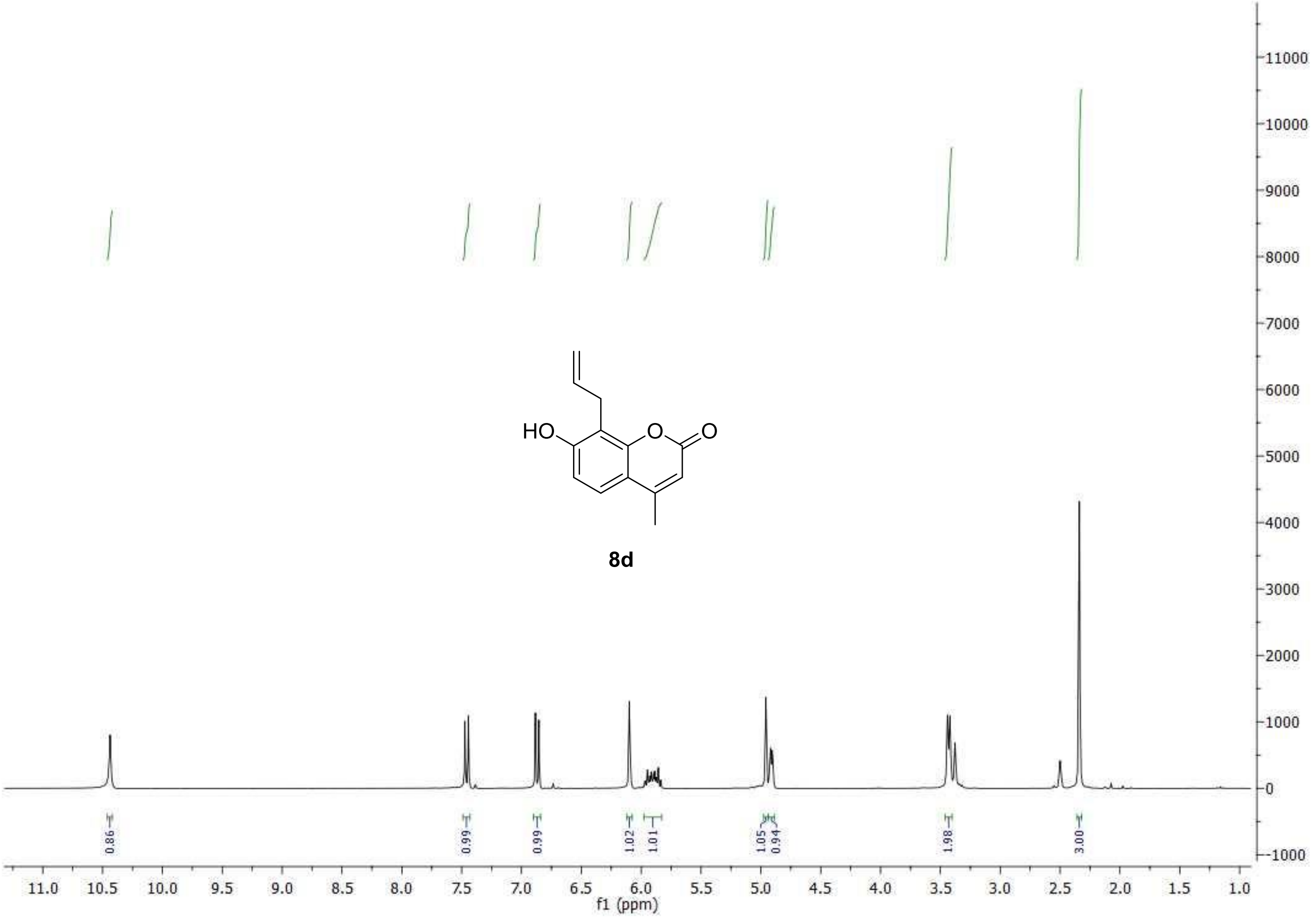

^{13}C NMR (75 MHz, DMSO-d_6) of **8d**

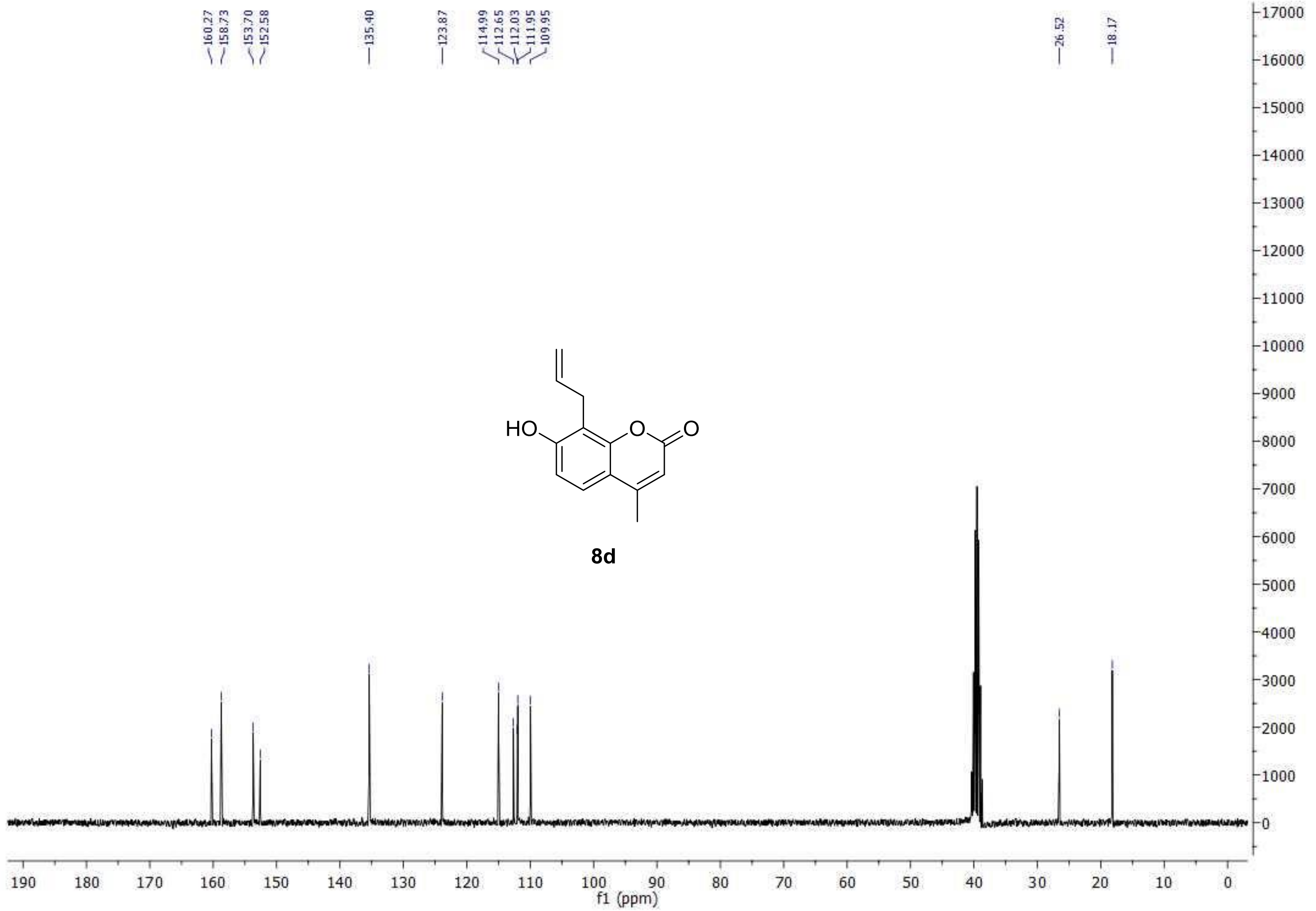

^{1}H NMR (300 MHz, $CDCl_3$) of **9a**

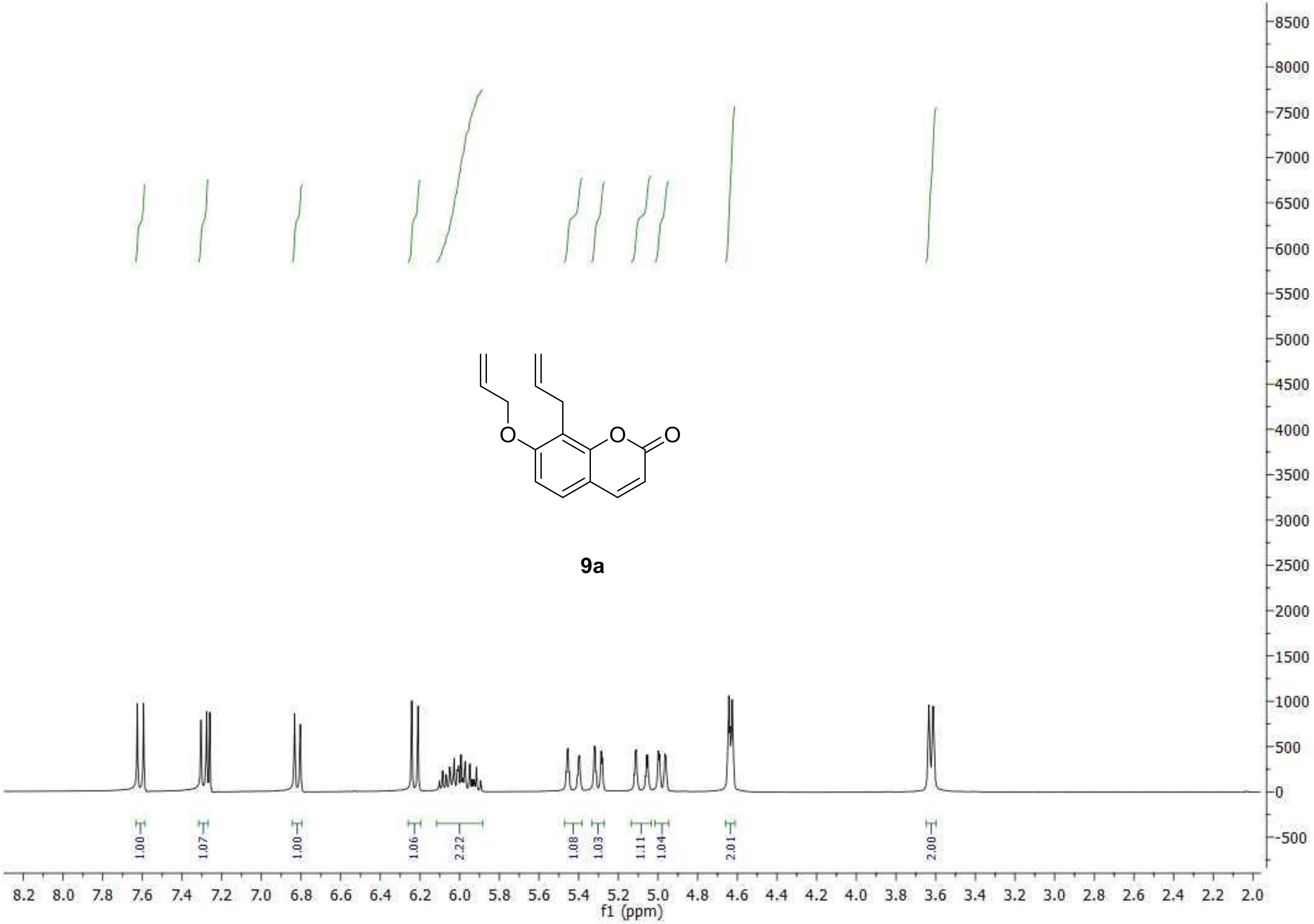

^{13}C NMR (75 MHz, $CDCl_3$) of **9a**

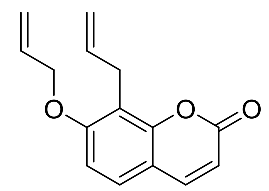

9a

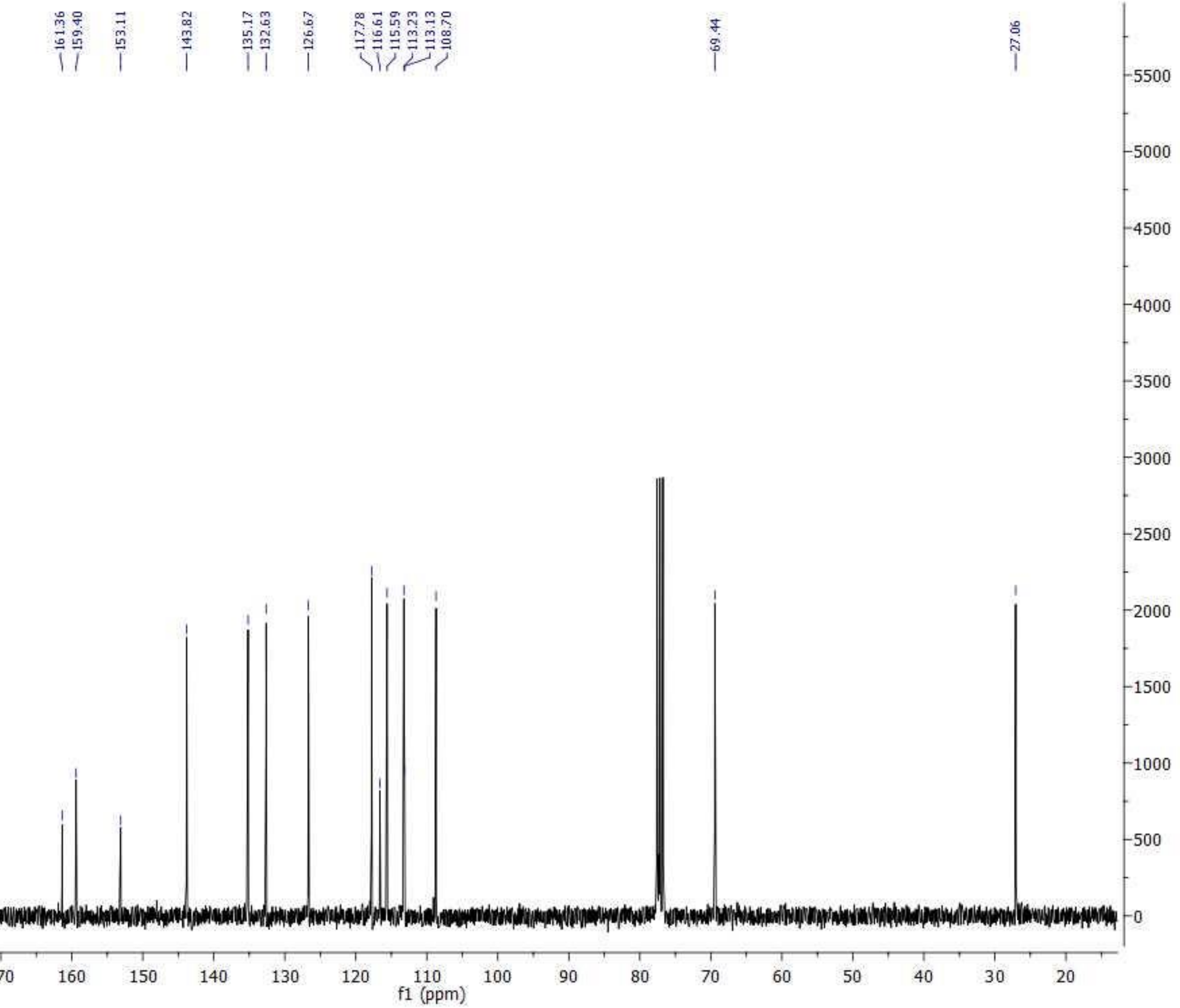

^{1}H NMR (300 MHz, $CDCl_3$) of **9b**

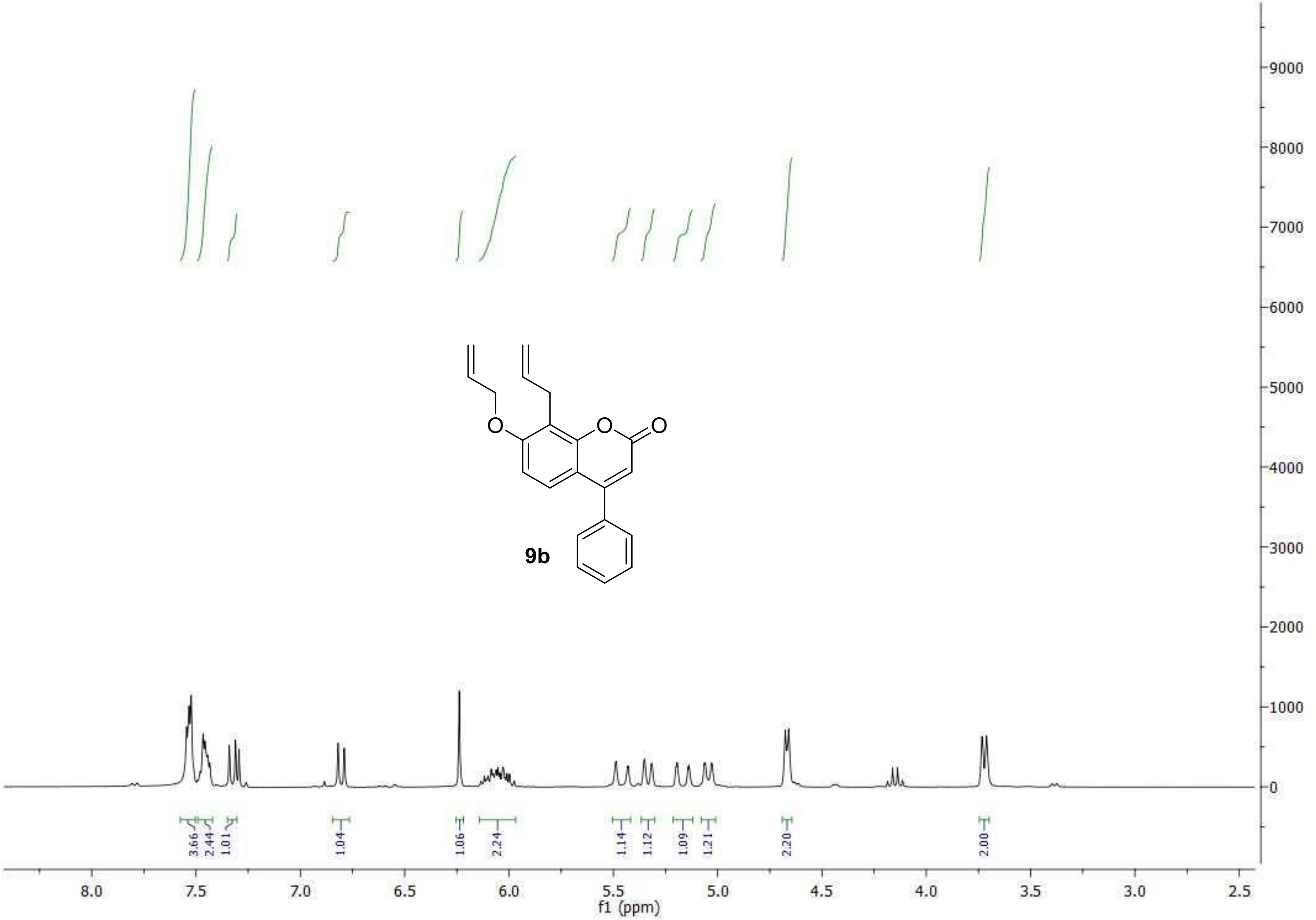

^{13}C NMR (75 MHz, $CDCl_3$) of **9b**

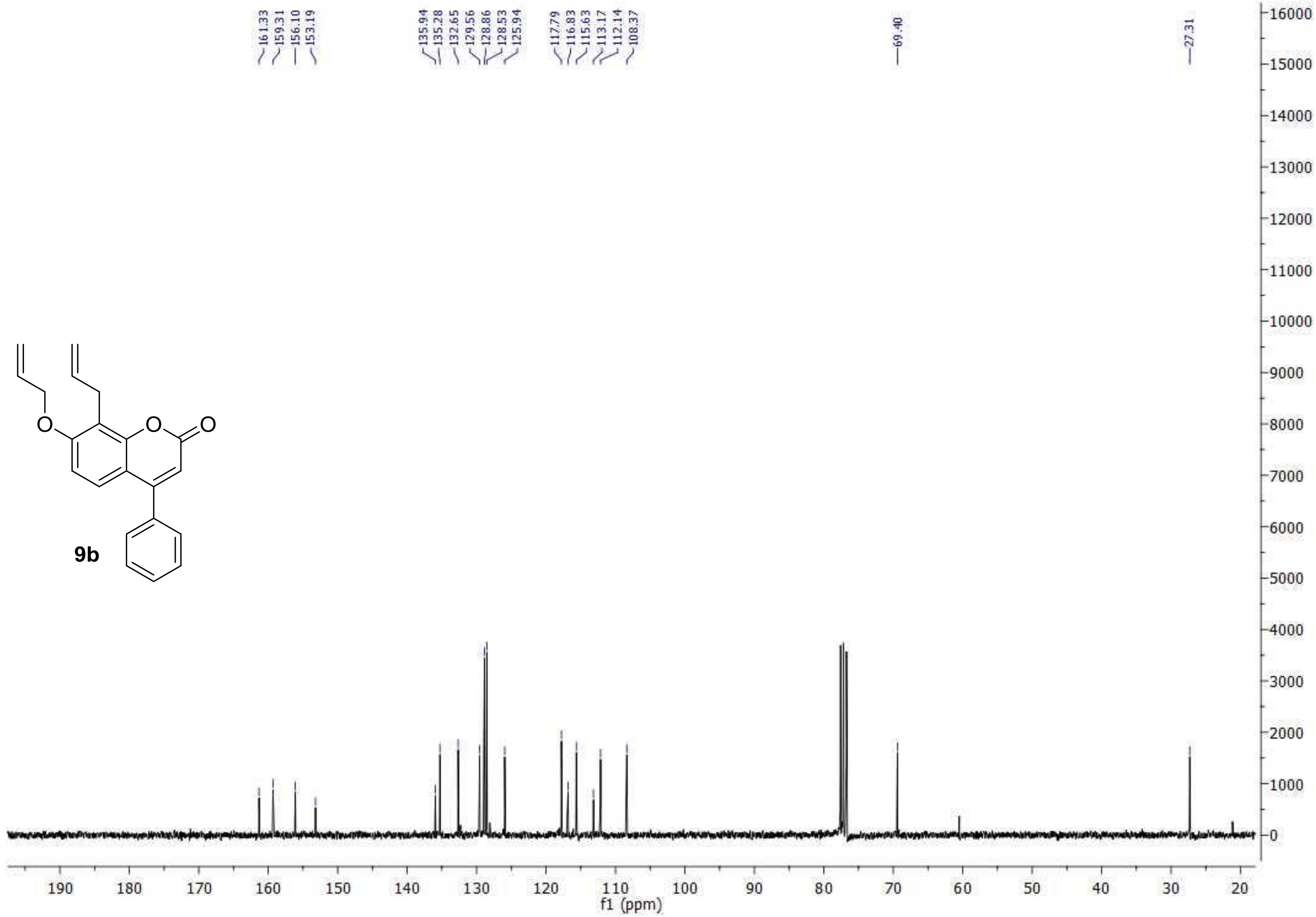
9b

^{1}H NMR (300 MHz, $CDCl_3$) of **9c**

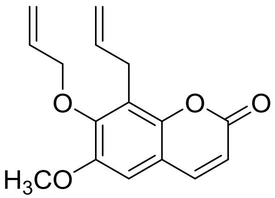

9c

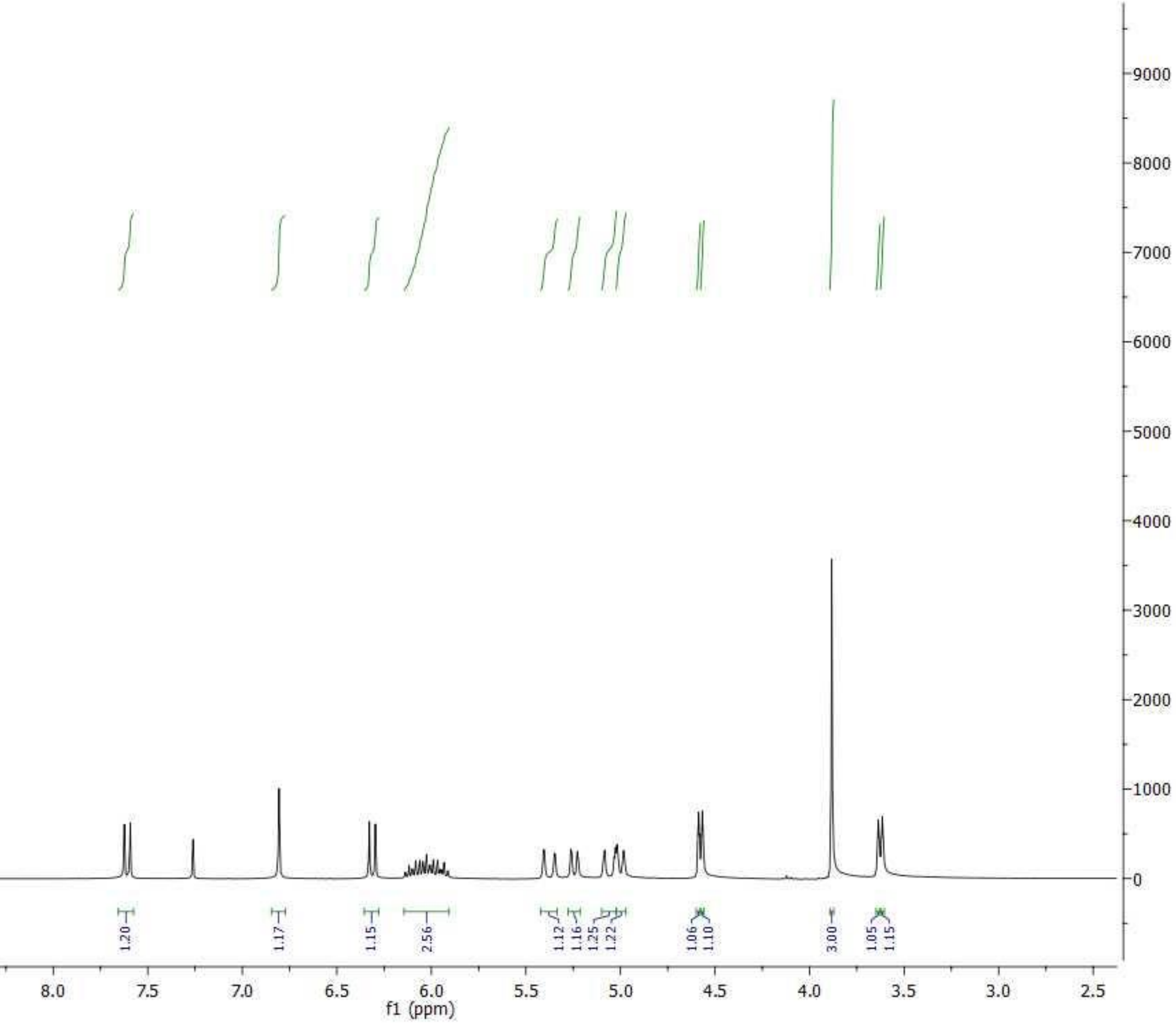

^{13}C NMR (75 MHz, $CDCl_3$) of **9c**

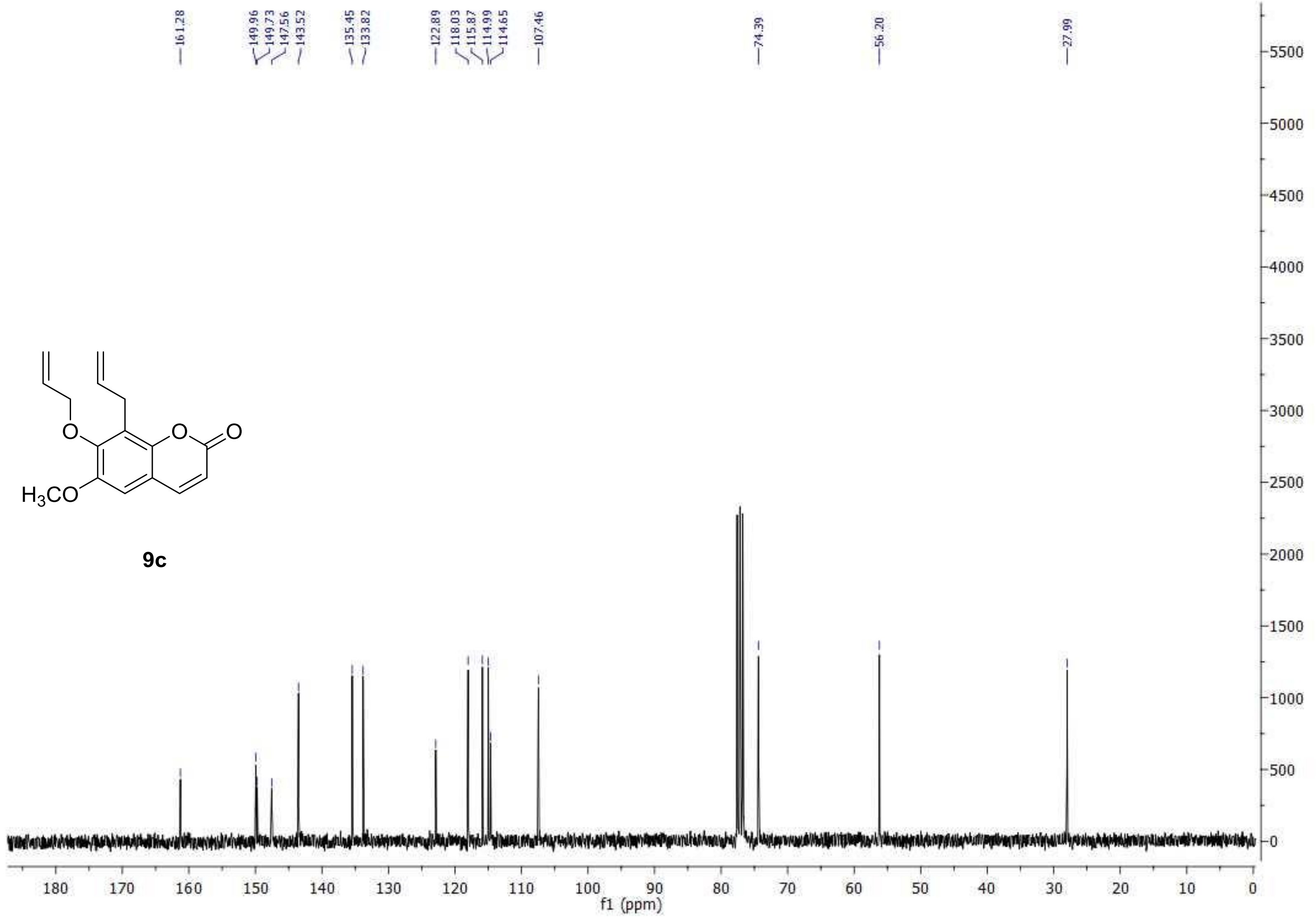

^{1}H NMR (300 MHz, $CDCl_3$) of **9d**

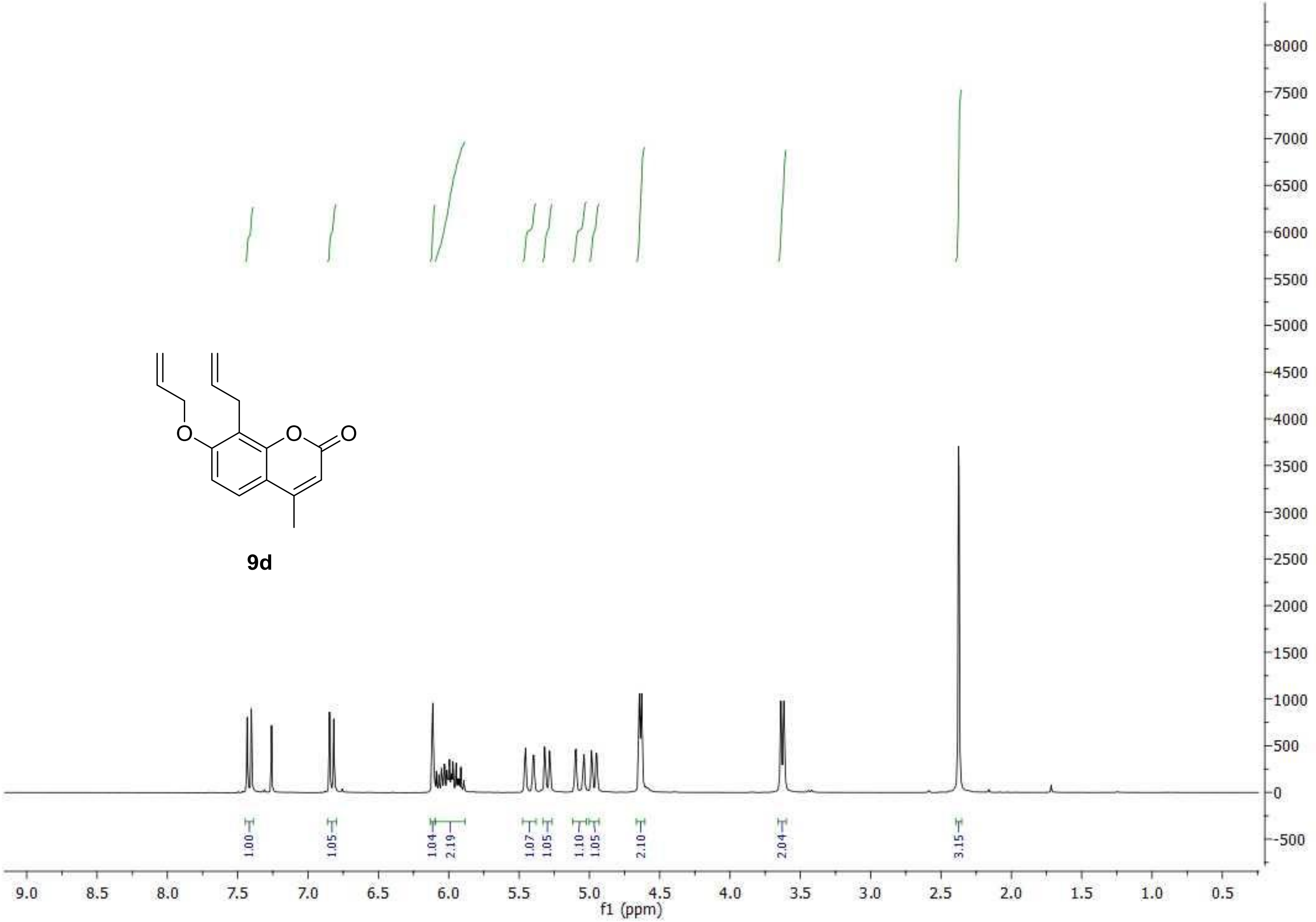

^{13}C NMR (75 MHz, $CDCl_3$) of **9d**

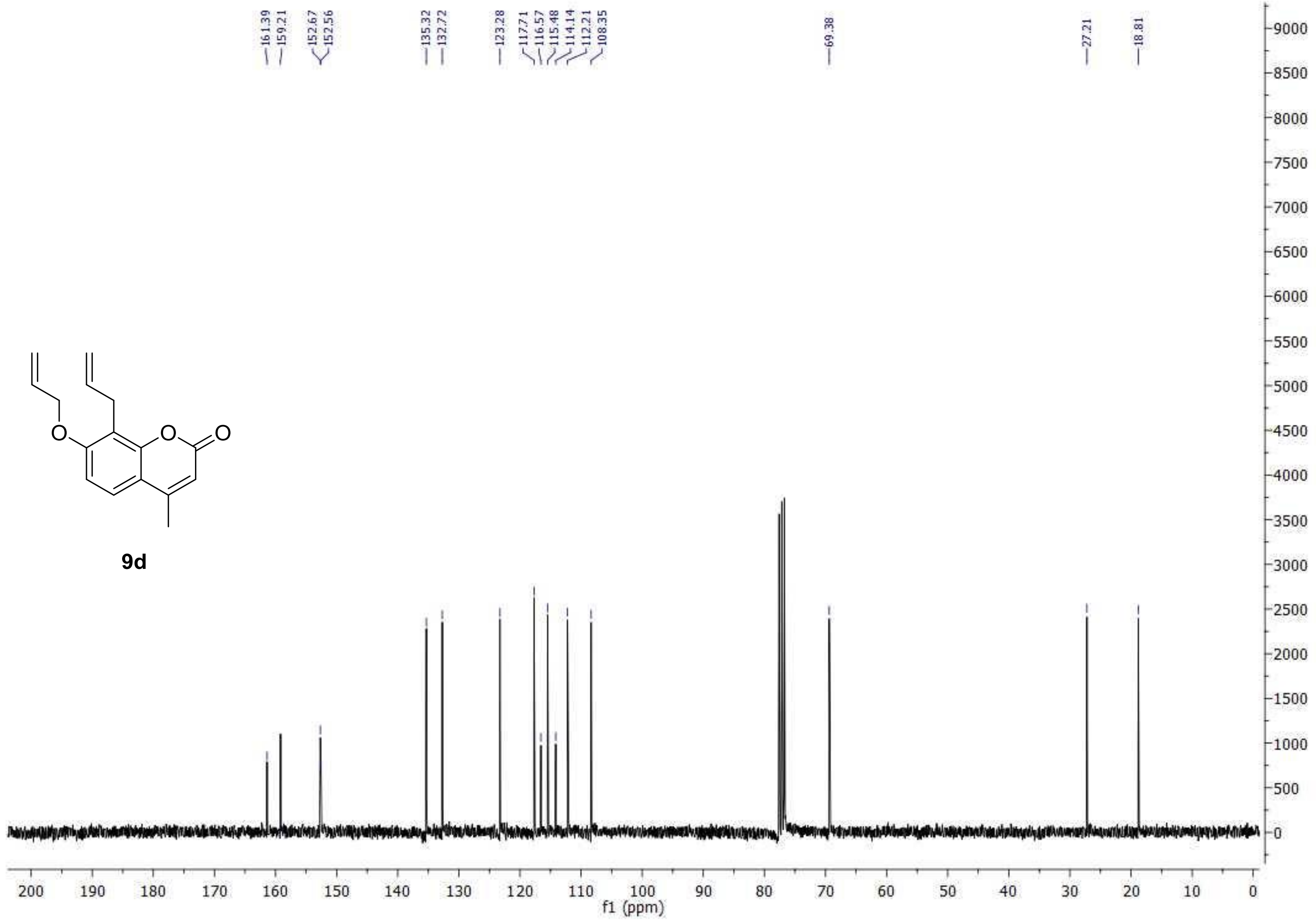

9d

^{1}H NMR (300 MHz, $CDCl_3$) of **10a**

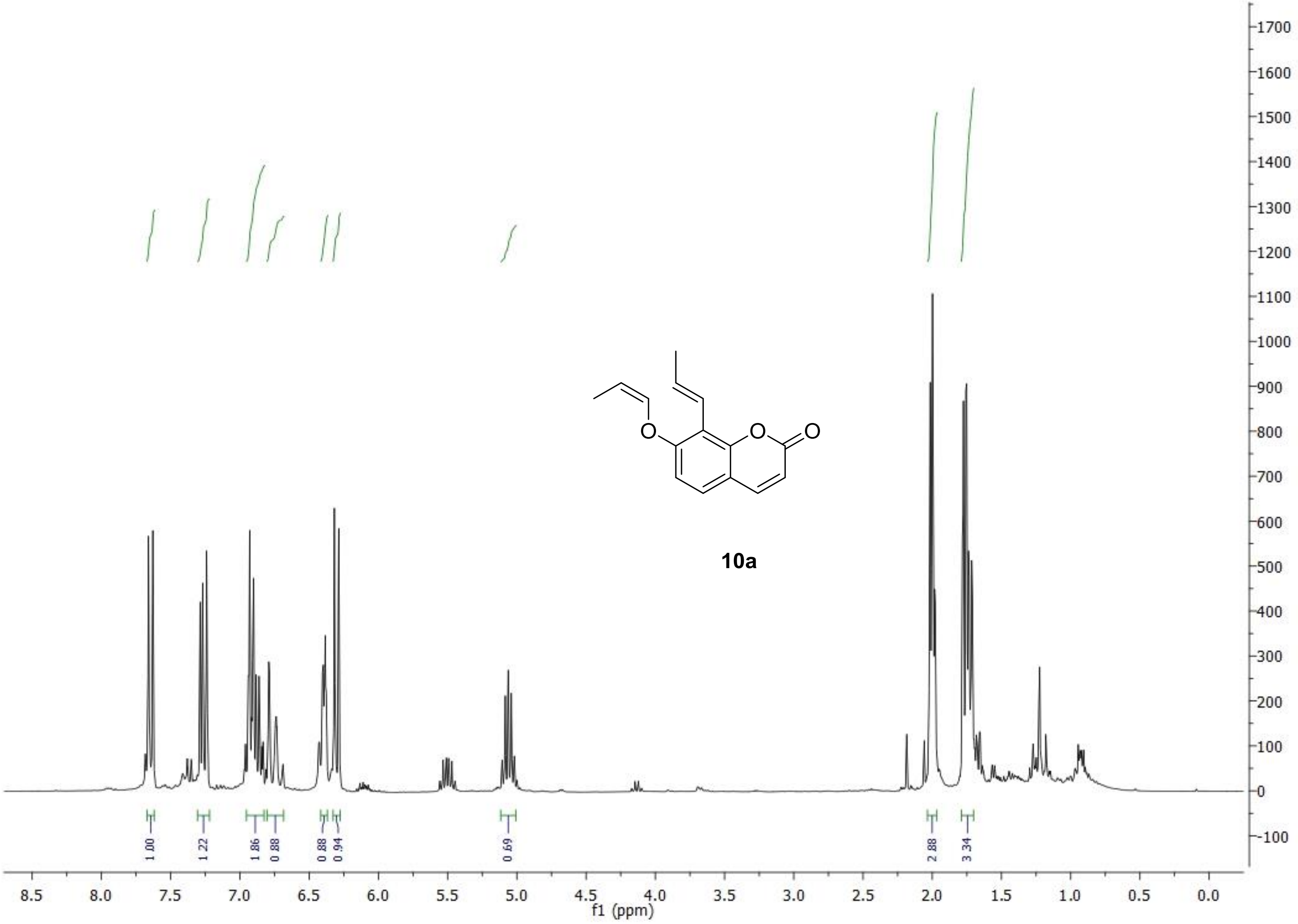

^{13}C NMR (75 MHz, $CDCl_3$) of **10a**

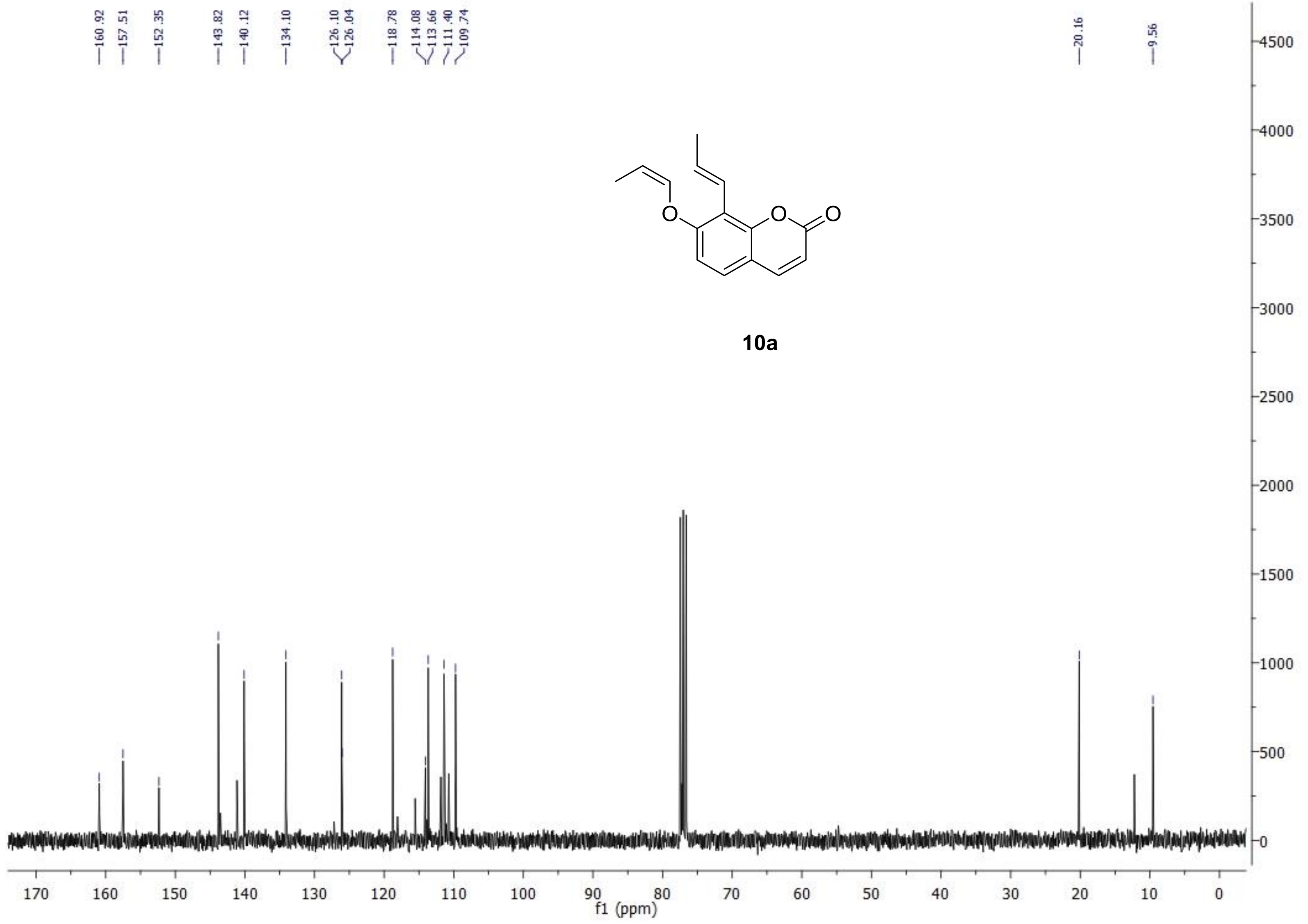

^{1}H NMR (300 MHz, $CDCl_3$) of **10b**

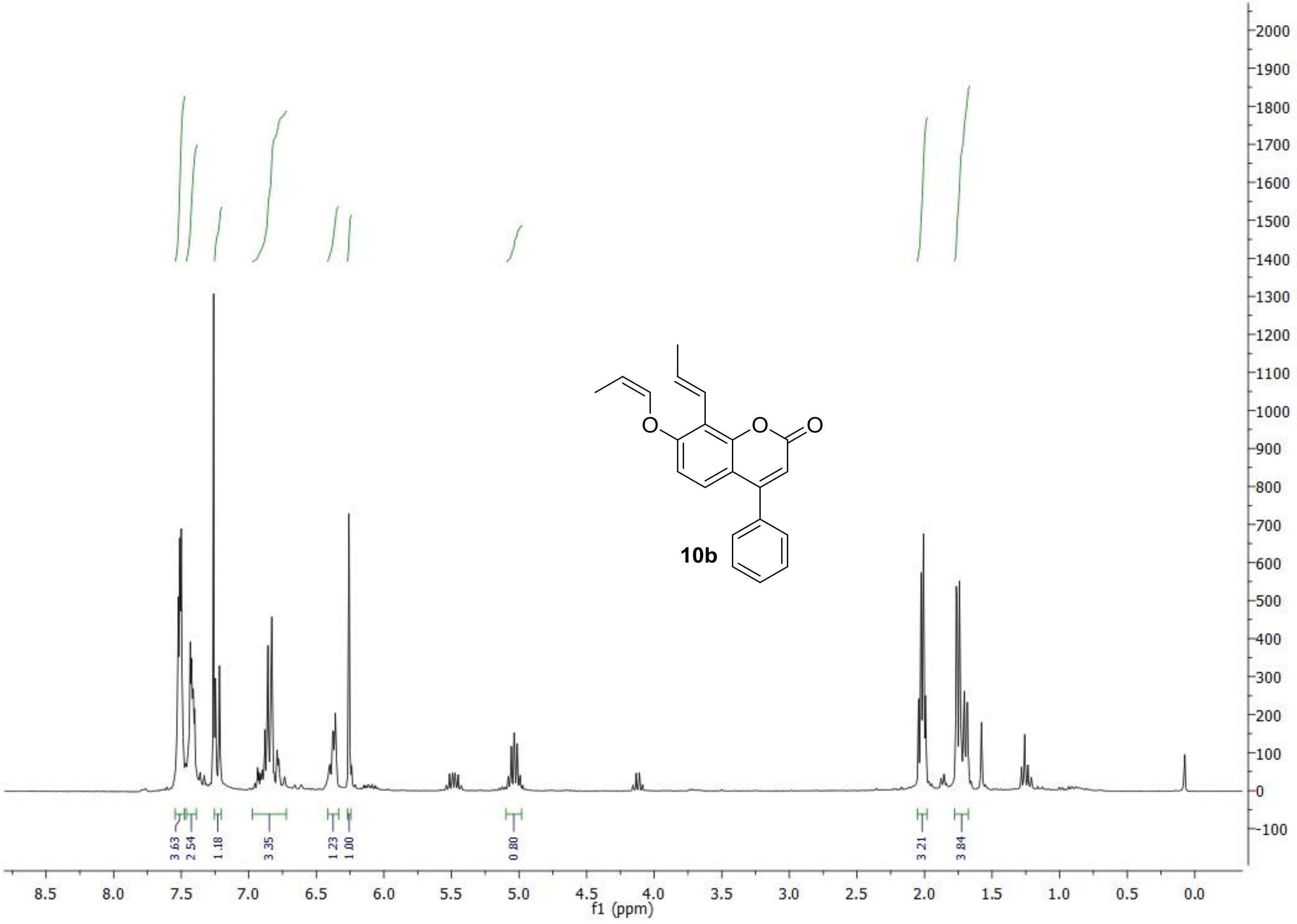

^{13}C NMR (150 MHz, $CDCl_3$) of **10b**

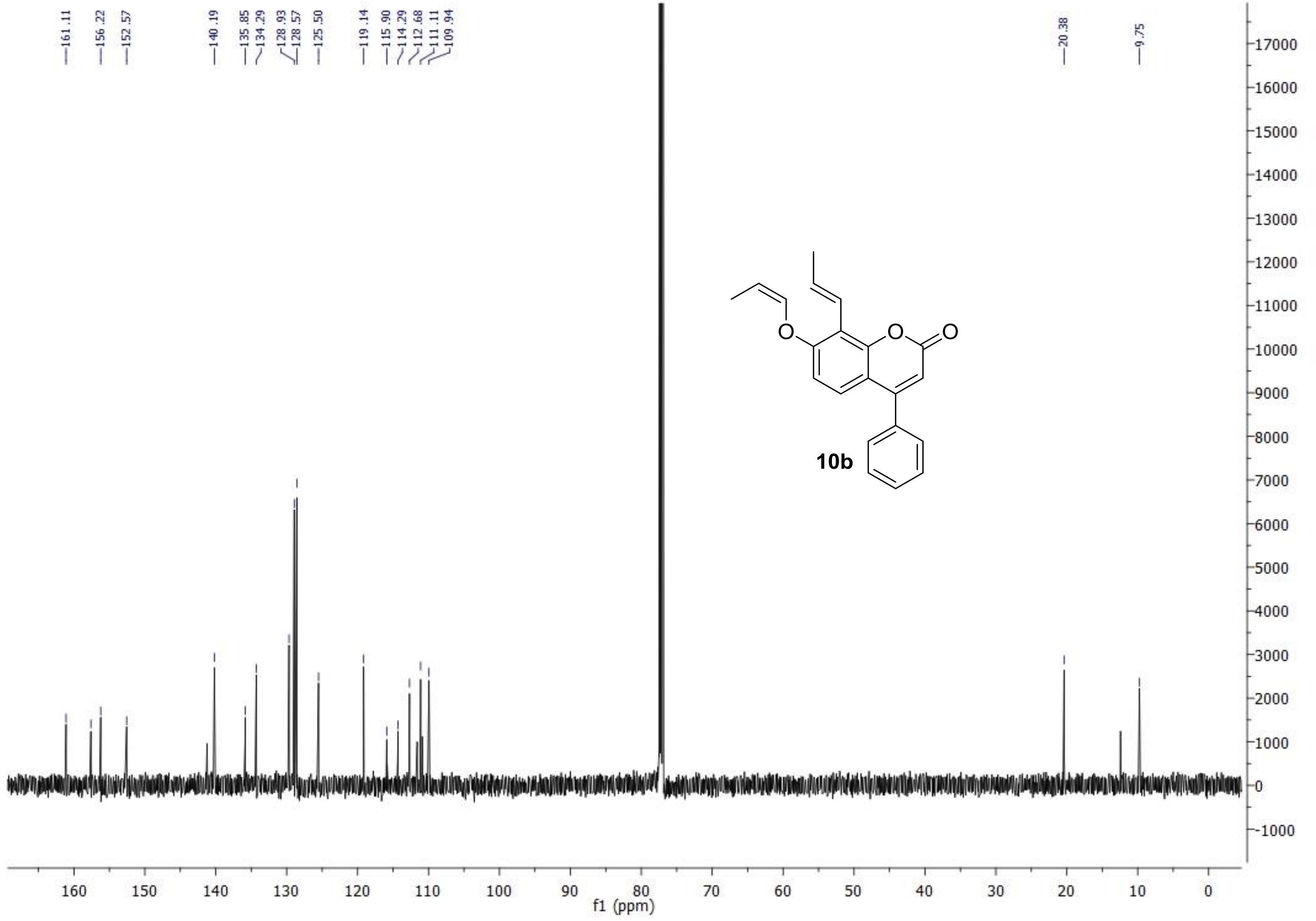

^{1}H NMR (300 MHz, $CDCl_3$) of **10c**

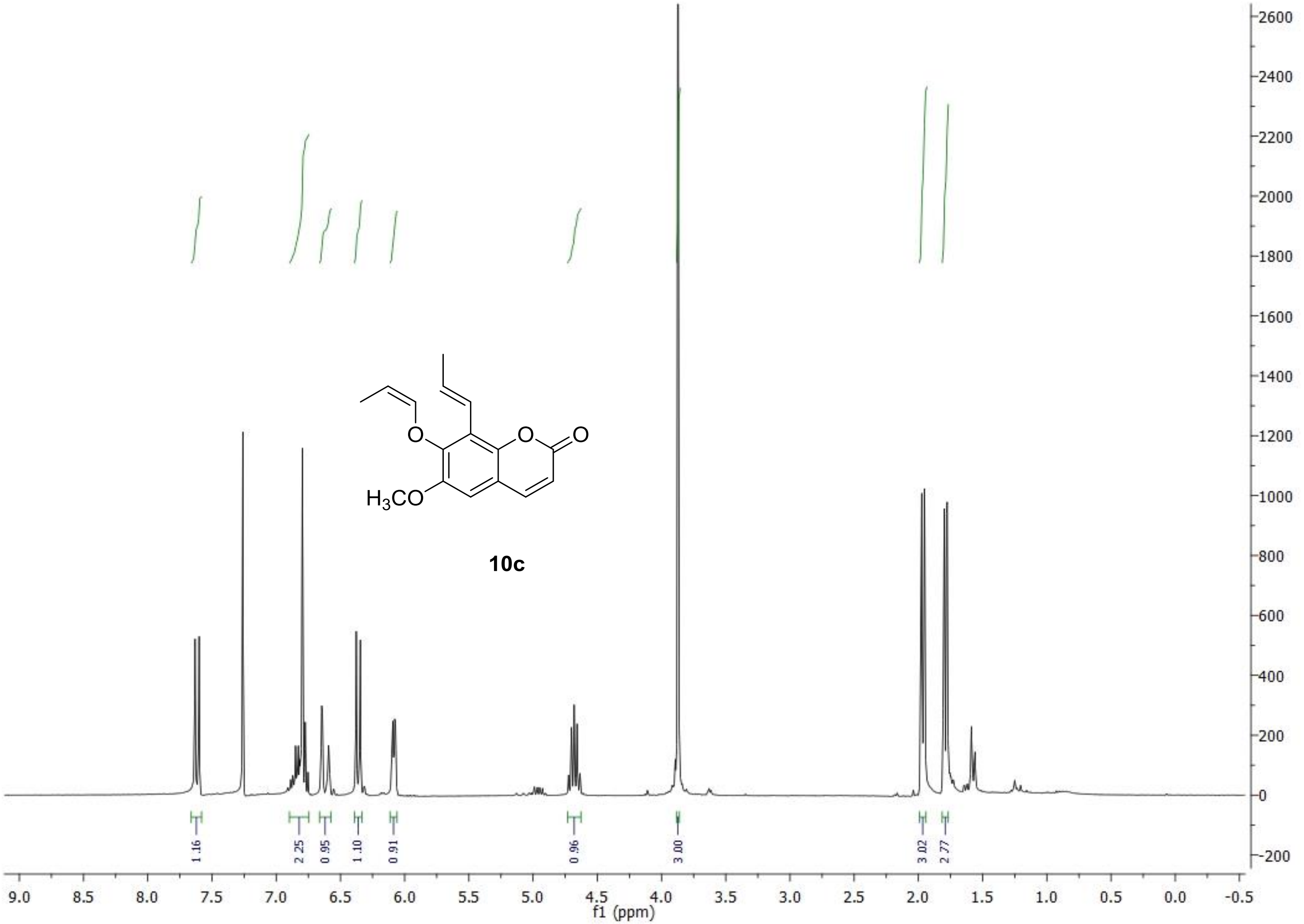

^{13}C NMR (150 MHz, $CDCl_3$) of **10c**

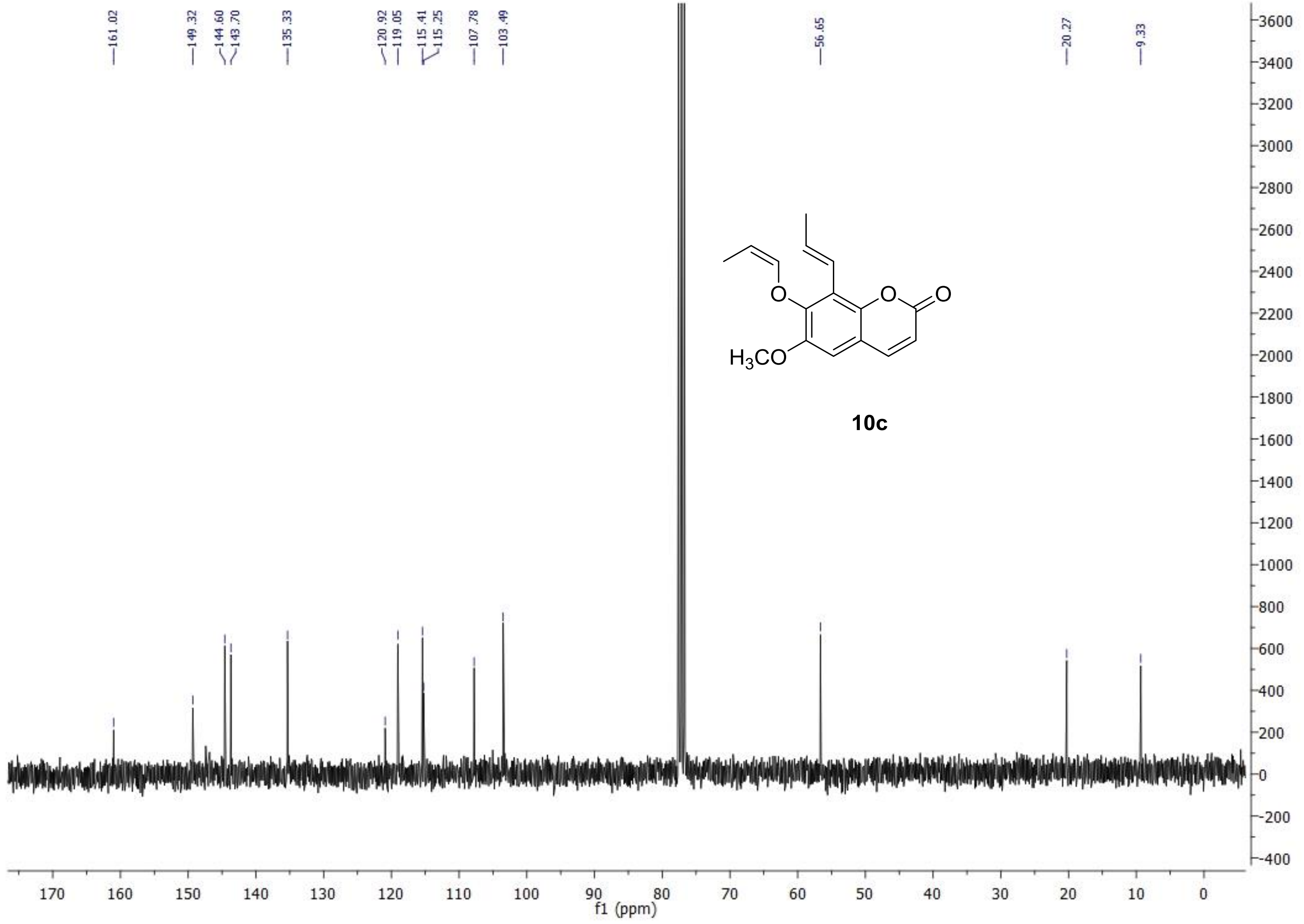

^{1}H NMR (300 MHz, $CDCl_3$) of **10d**

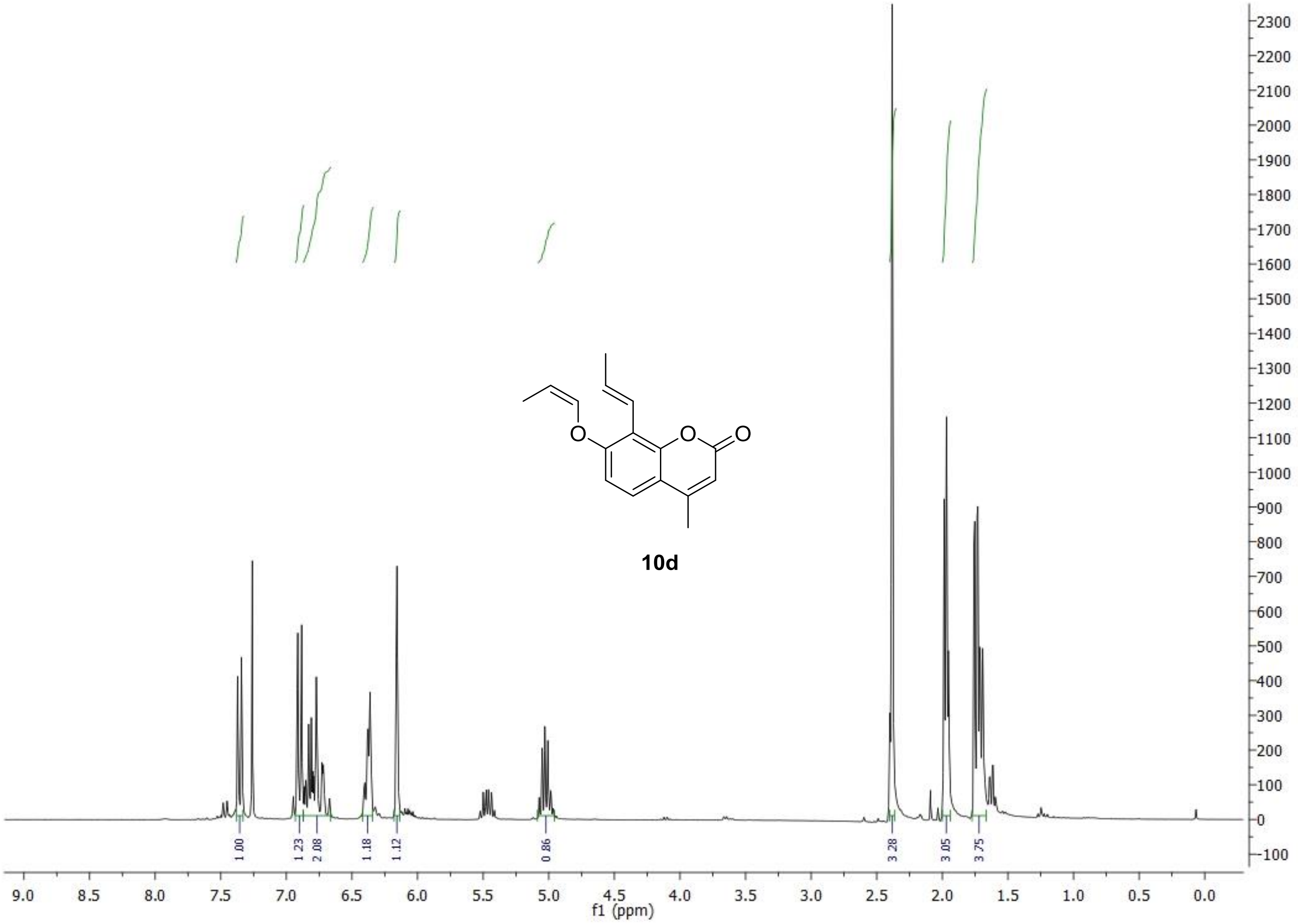

^{13}C NMR (150 MHz, $CDCl_3$) of **10d**

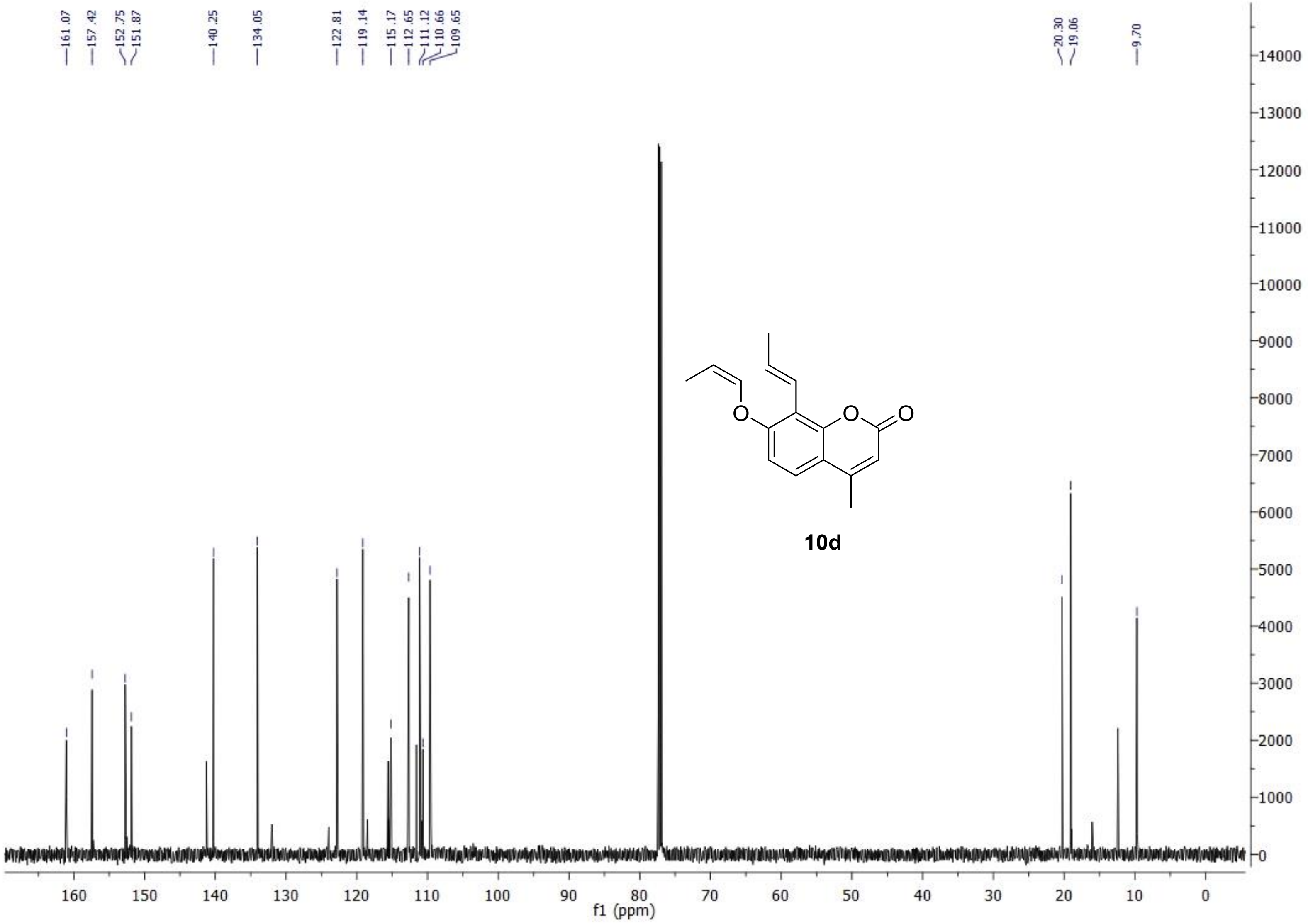

^{1}H NMR (300 MHz, $CDCl_3$) of **11a**

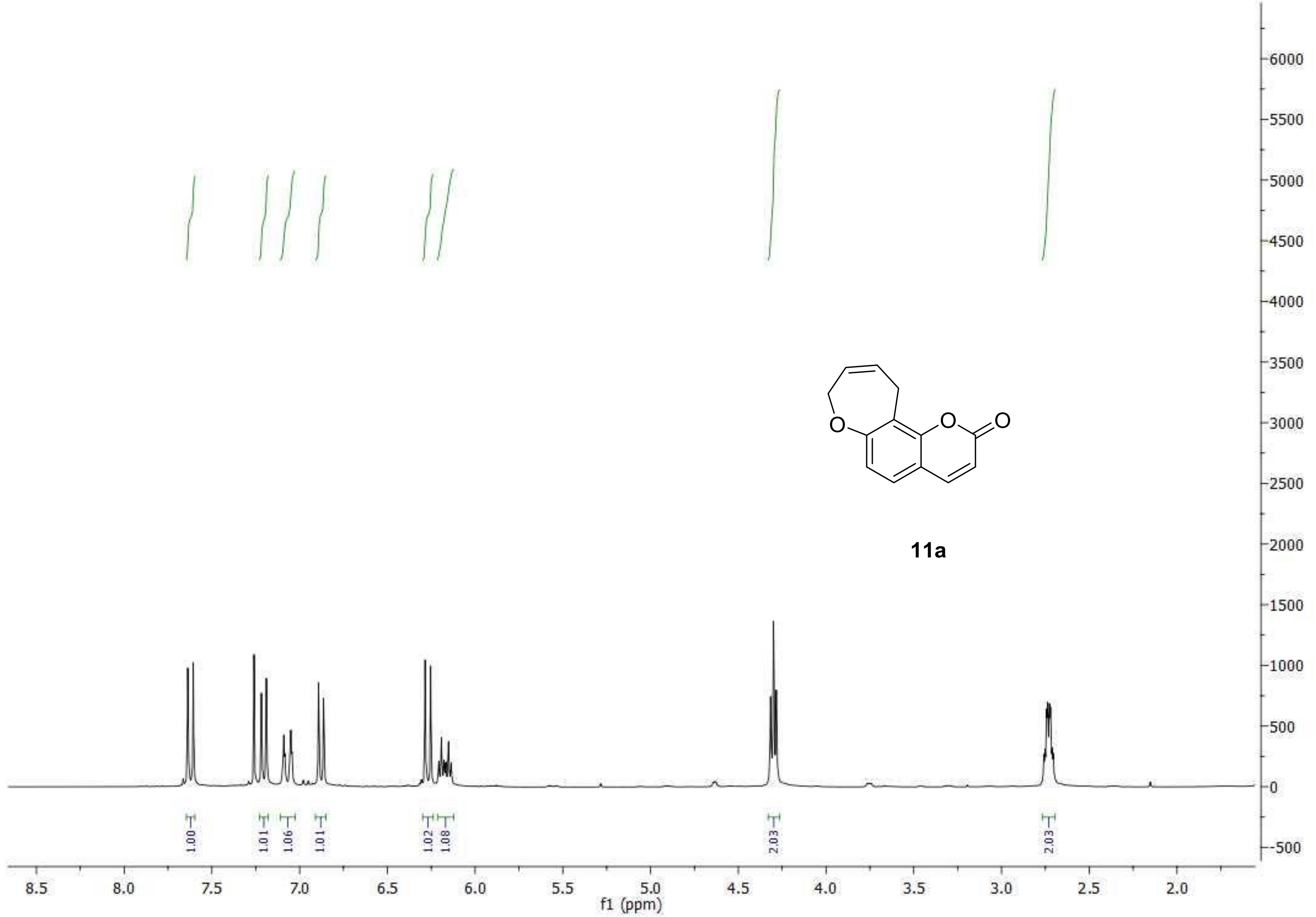

^{13}C NMR (75 MHz, $CDCl_3$) of **11a**

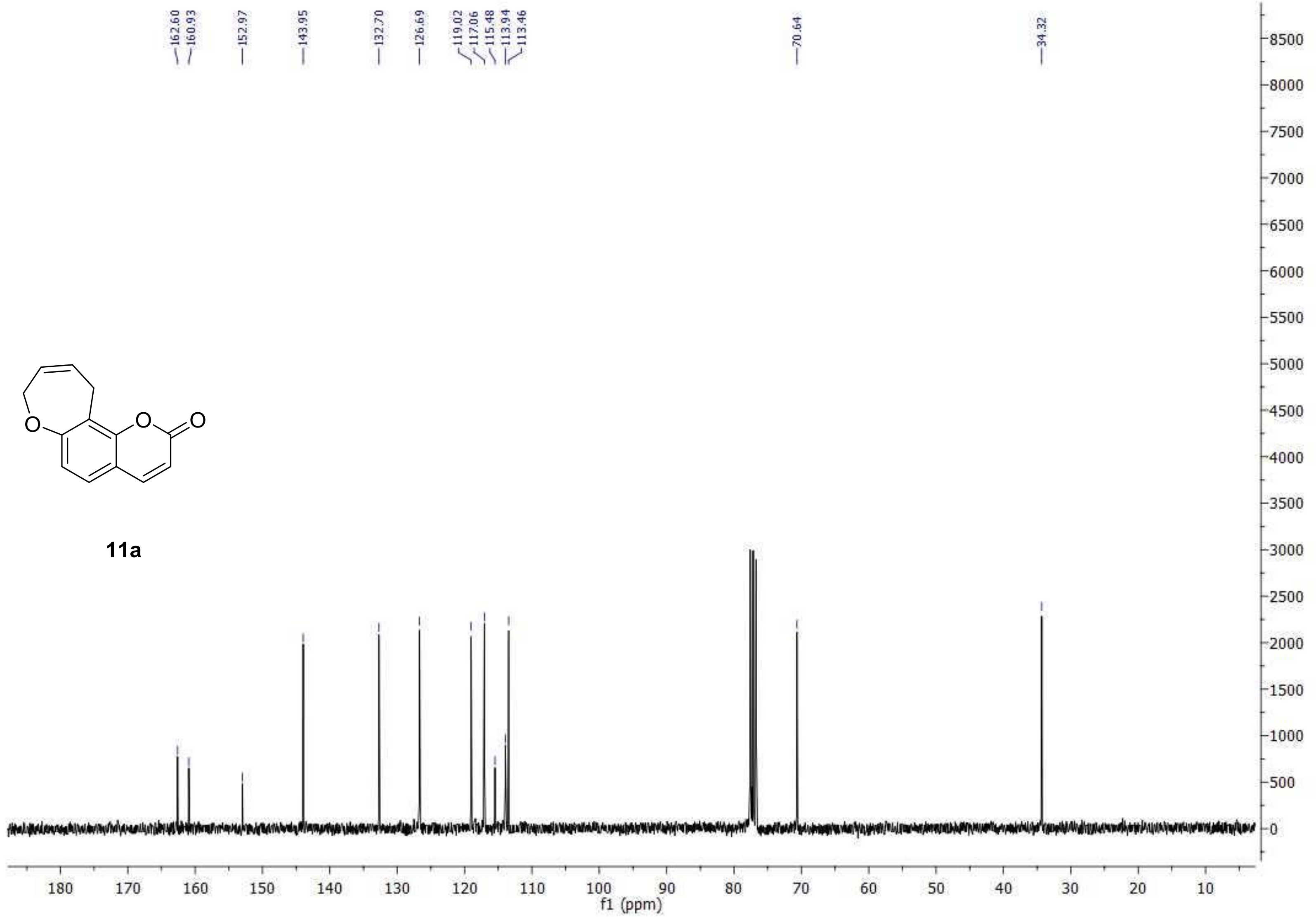

^{1}H NMR (300 MHz, $CDCl_3$) of **11b**

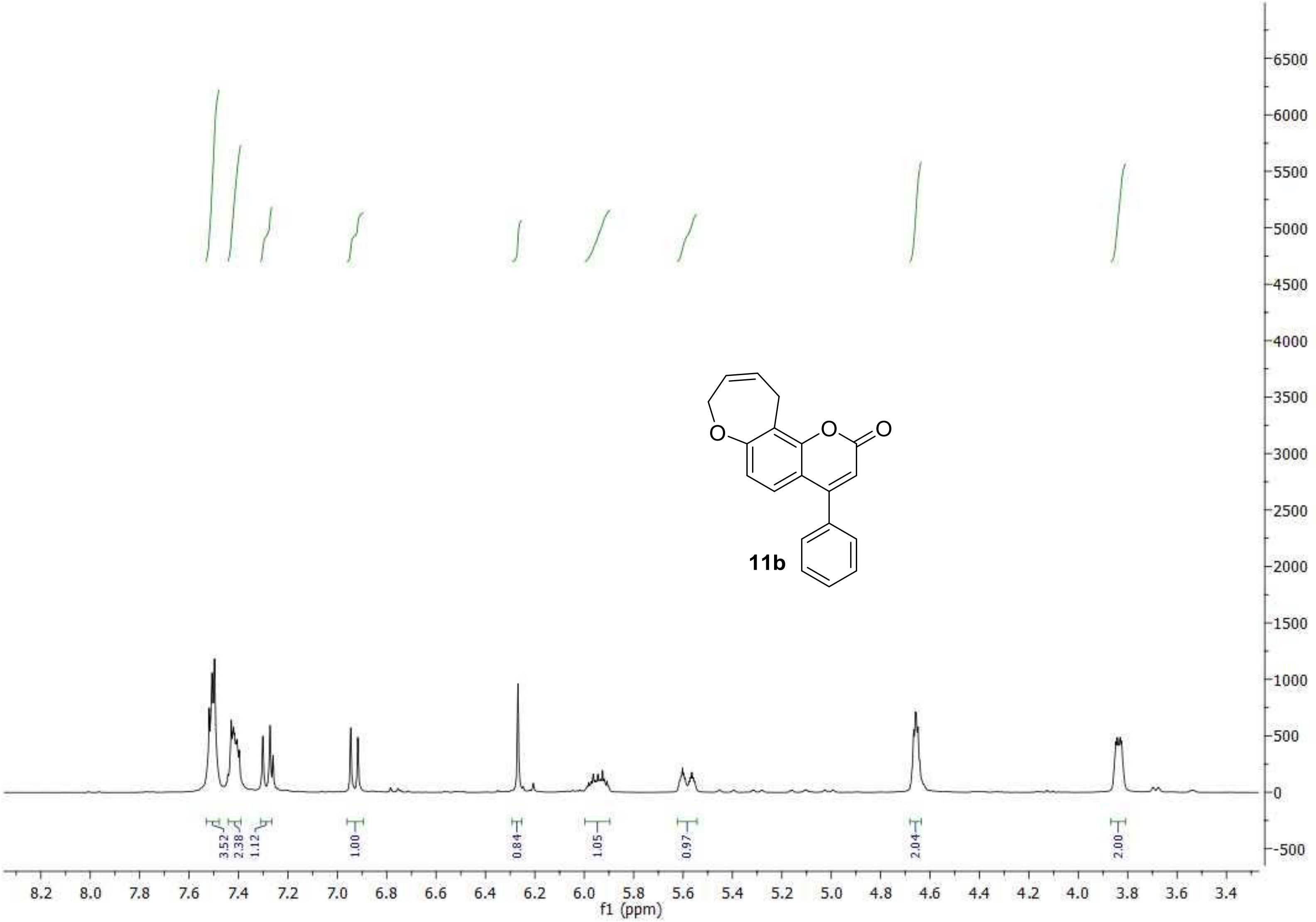

^{13}C NMR (75 MHz, $CDCl_3$) of **11b**

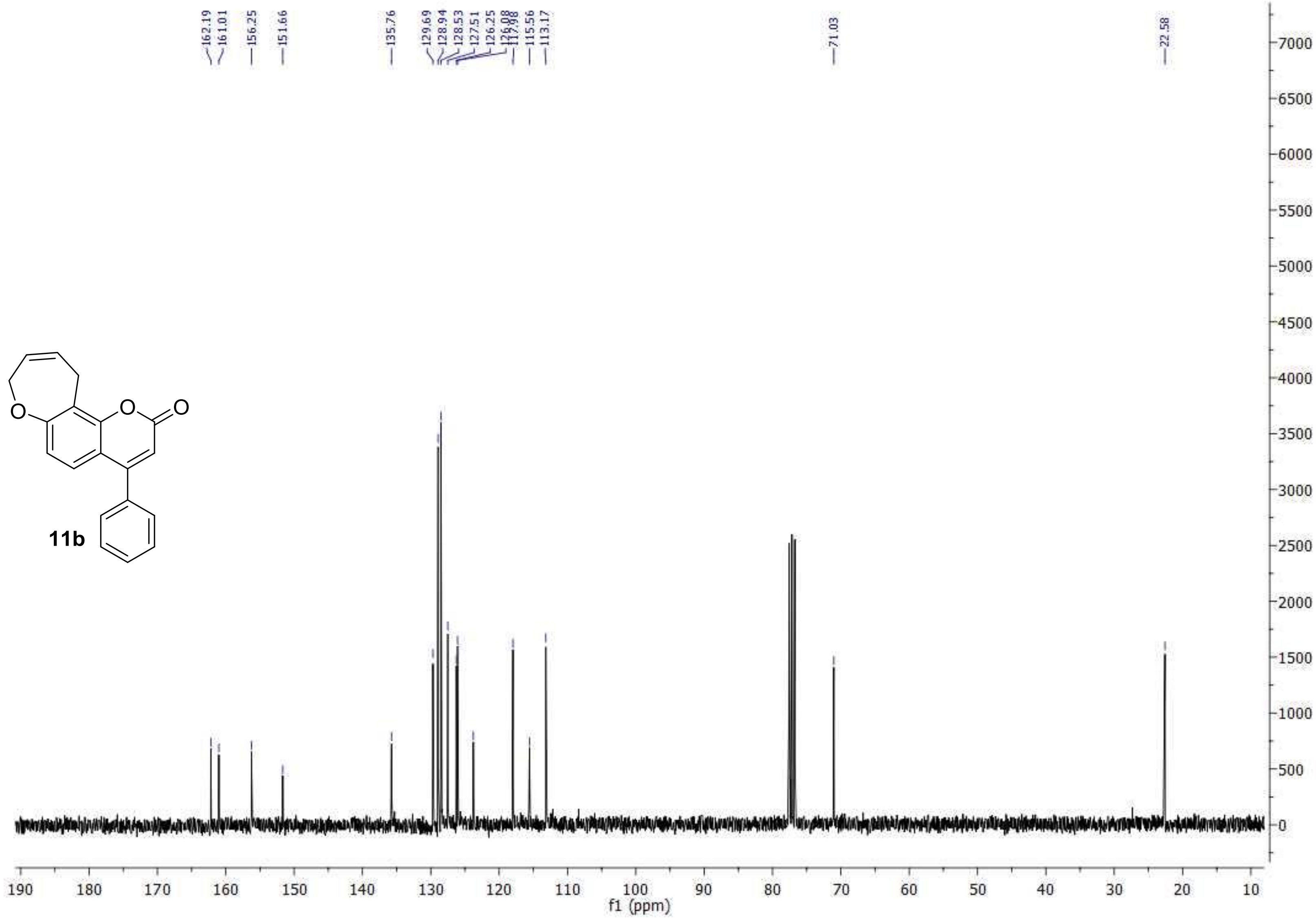

^{1}H NMR (300 MHz, $CDCl_3$) of **11c**

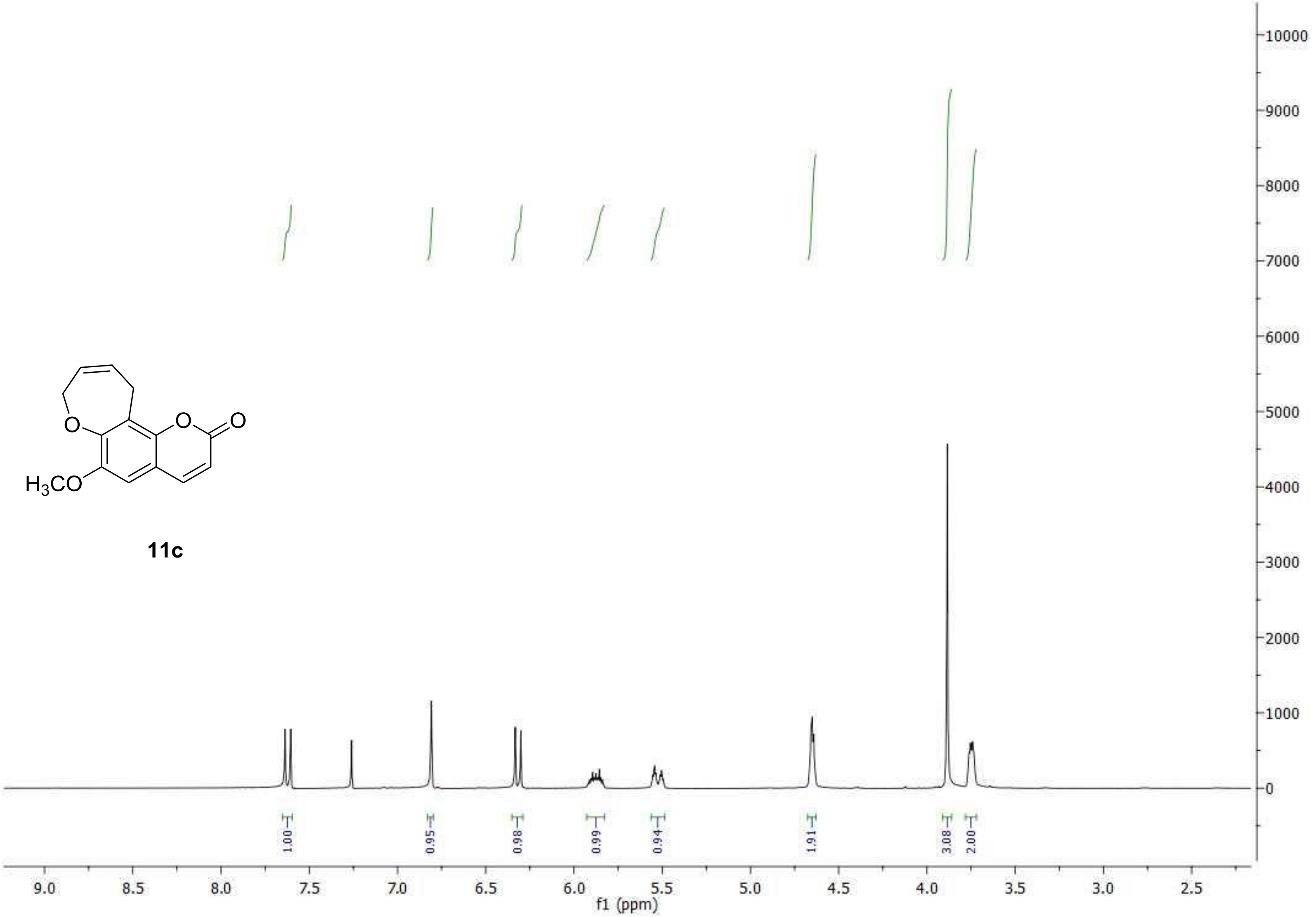

^{13}C NMR (75 MHz, $CDCl_3$) of **11c**

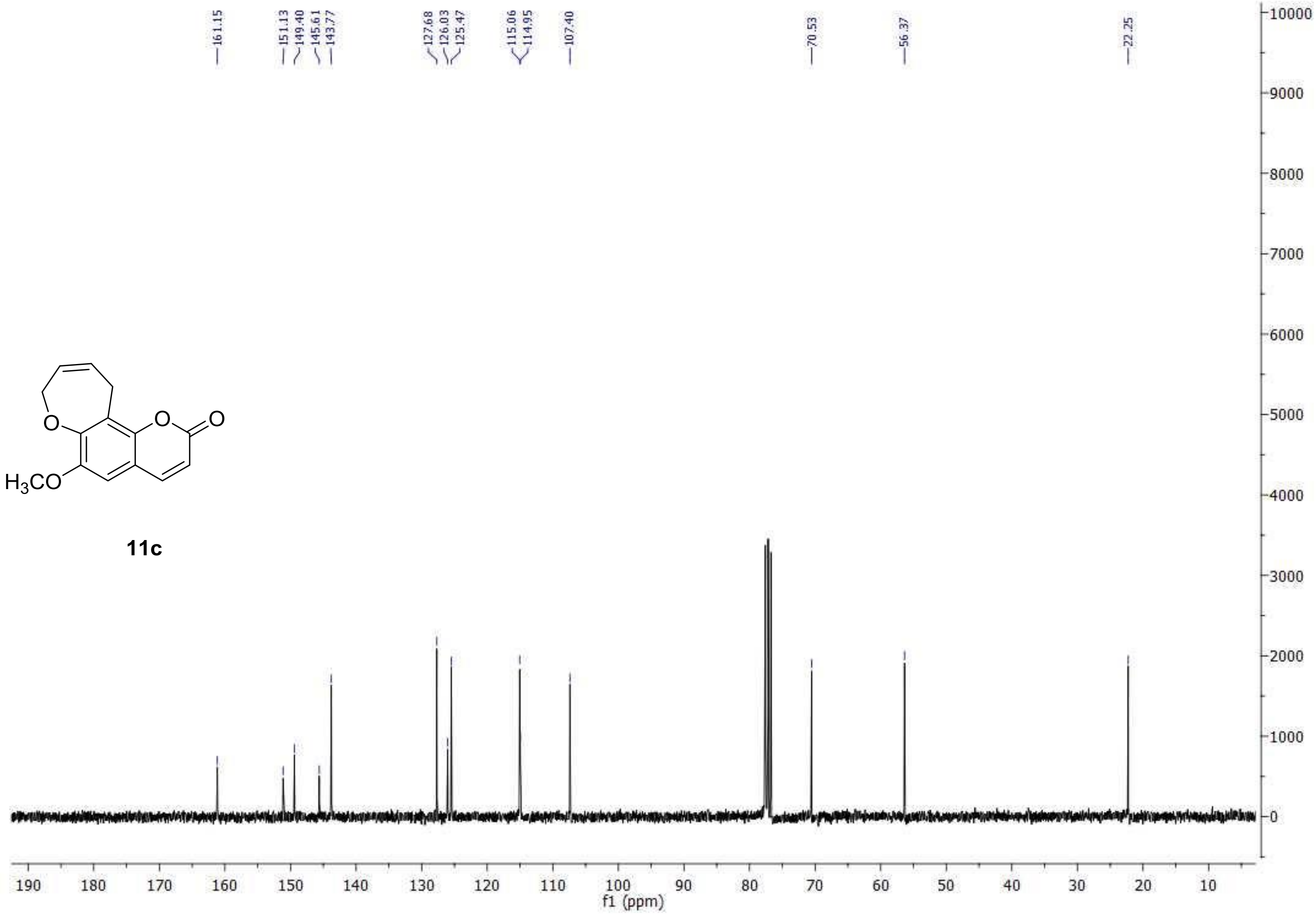

^{1}H NMR (300 MHz, $CDCl_3$) of **11d**

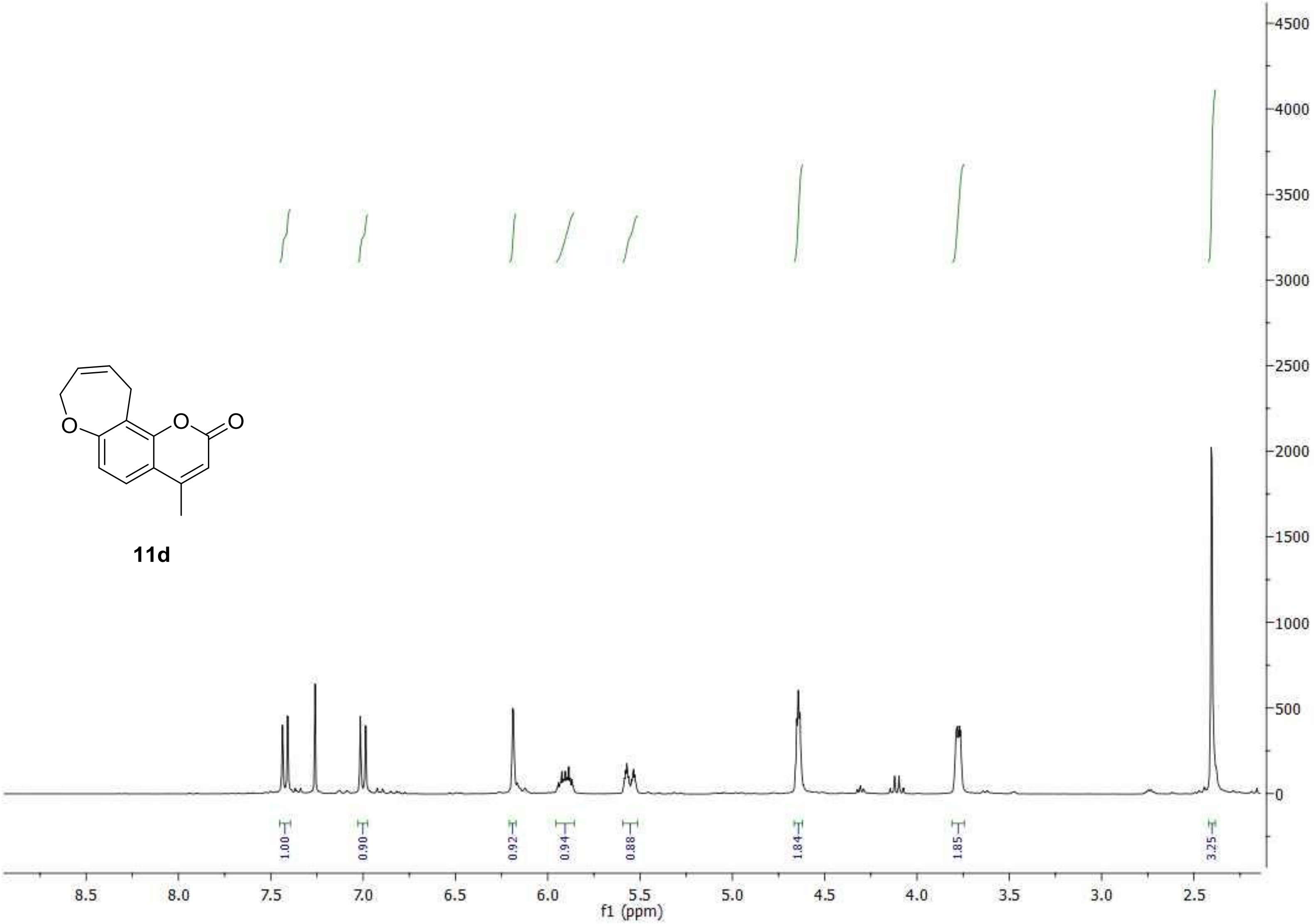

11d

^{13}C NMR (75 MHz, $CDCl_3$) of **11d**

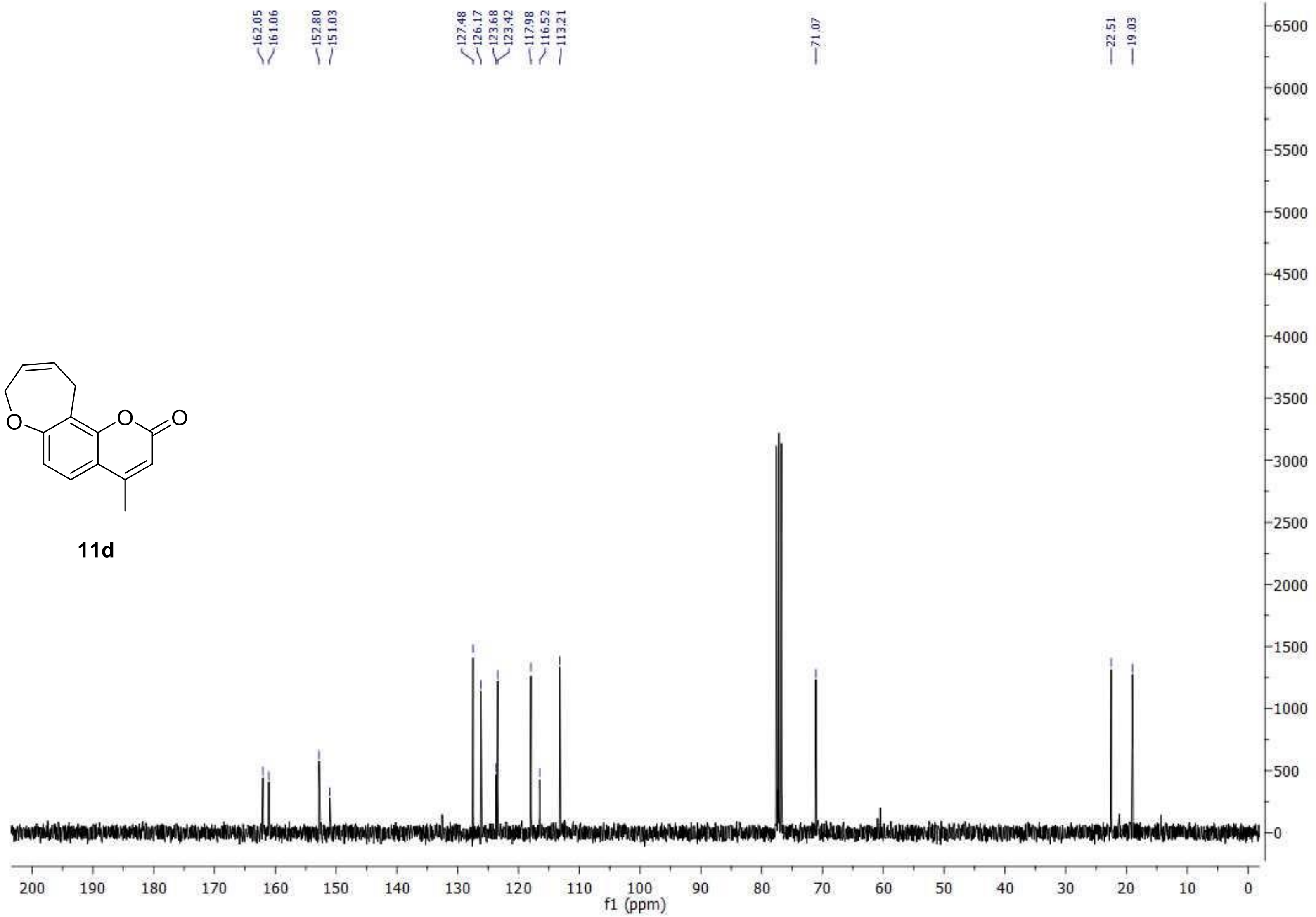

11d

^{1}H NMR (300 MHz, $CDCl_3$) of **3a**

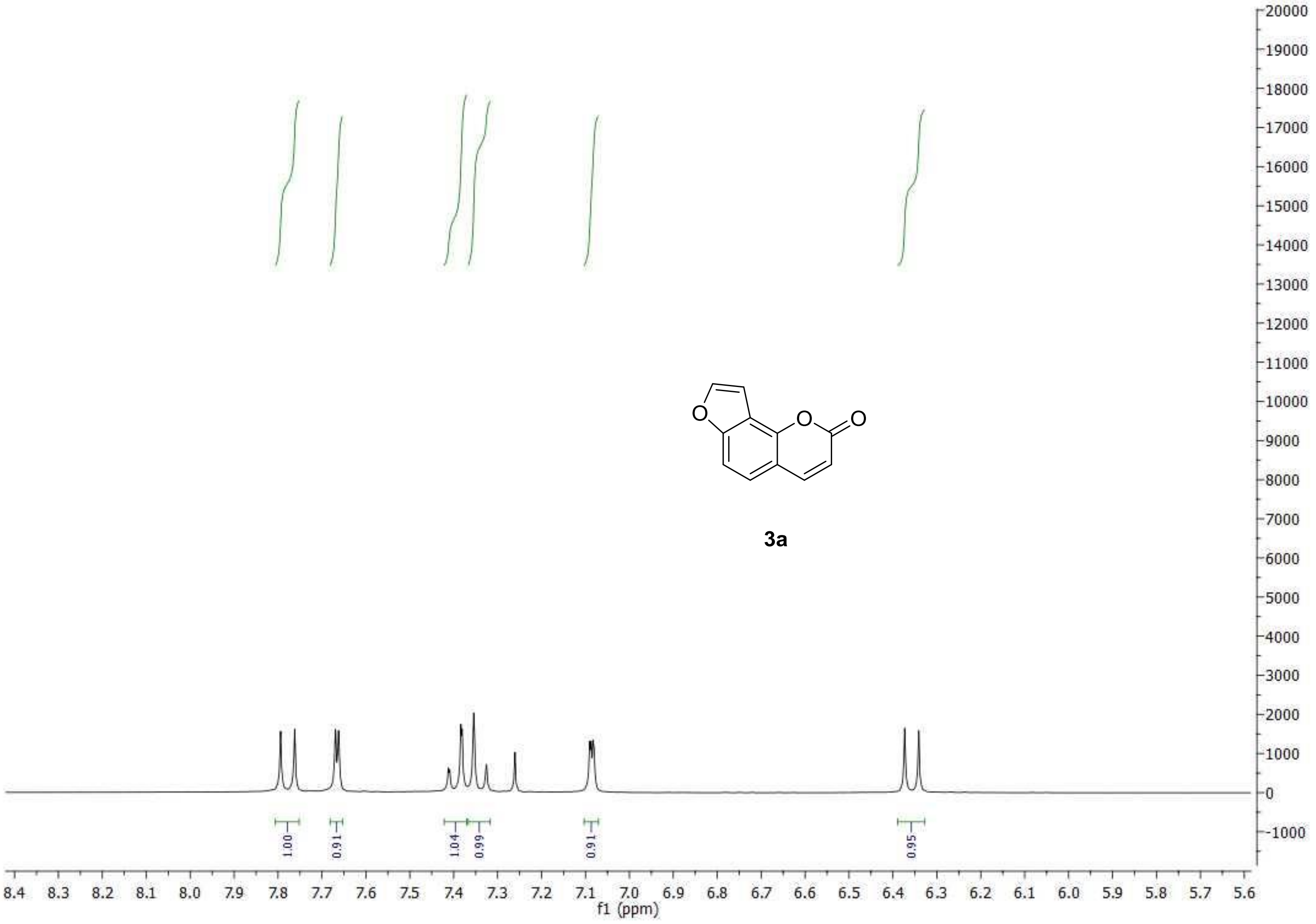

^{13}C NMR (75 MHz, CDCl$_3$) of **3a**

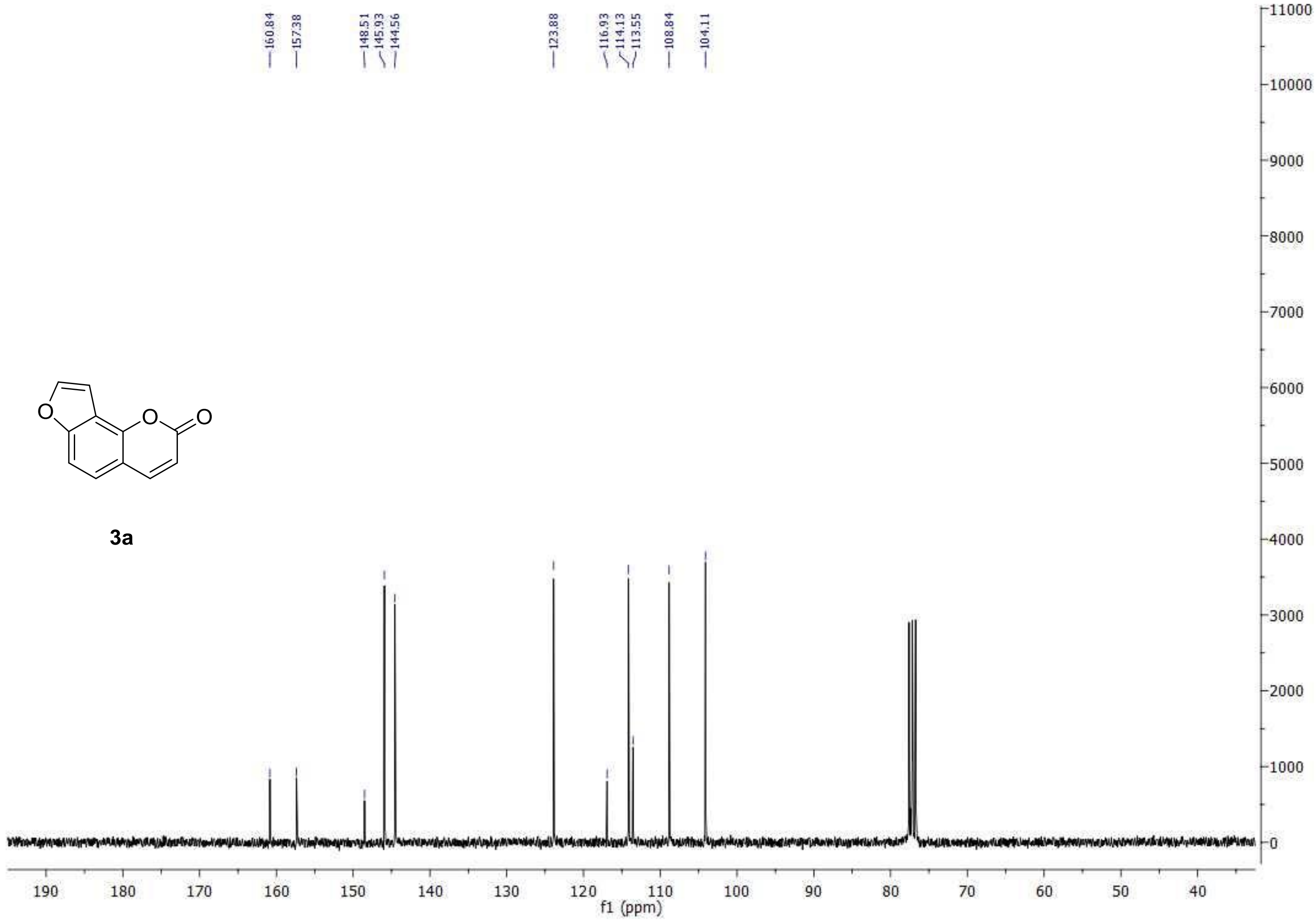

3a

^{1}H NMR (300 MHz, $CDCl_3$) of **3b**

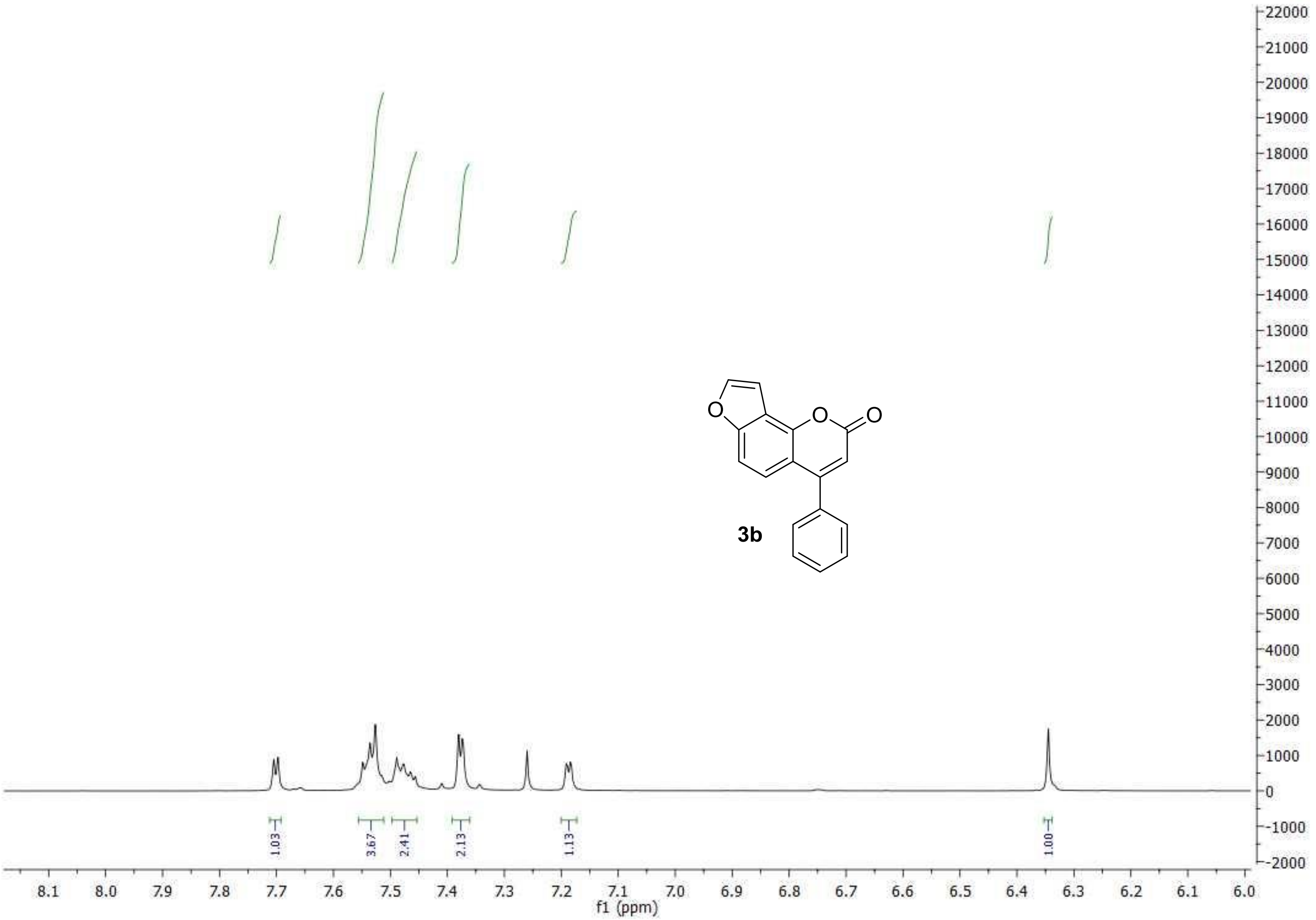

^{13}C NMR (75 MHz, $CDCl_3$) of **3b**

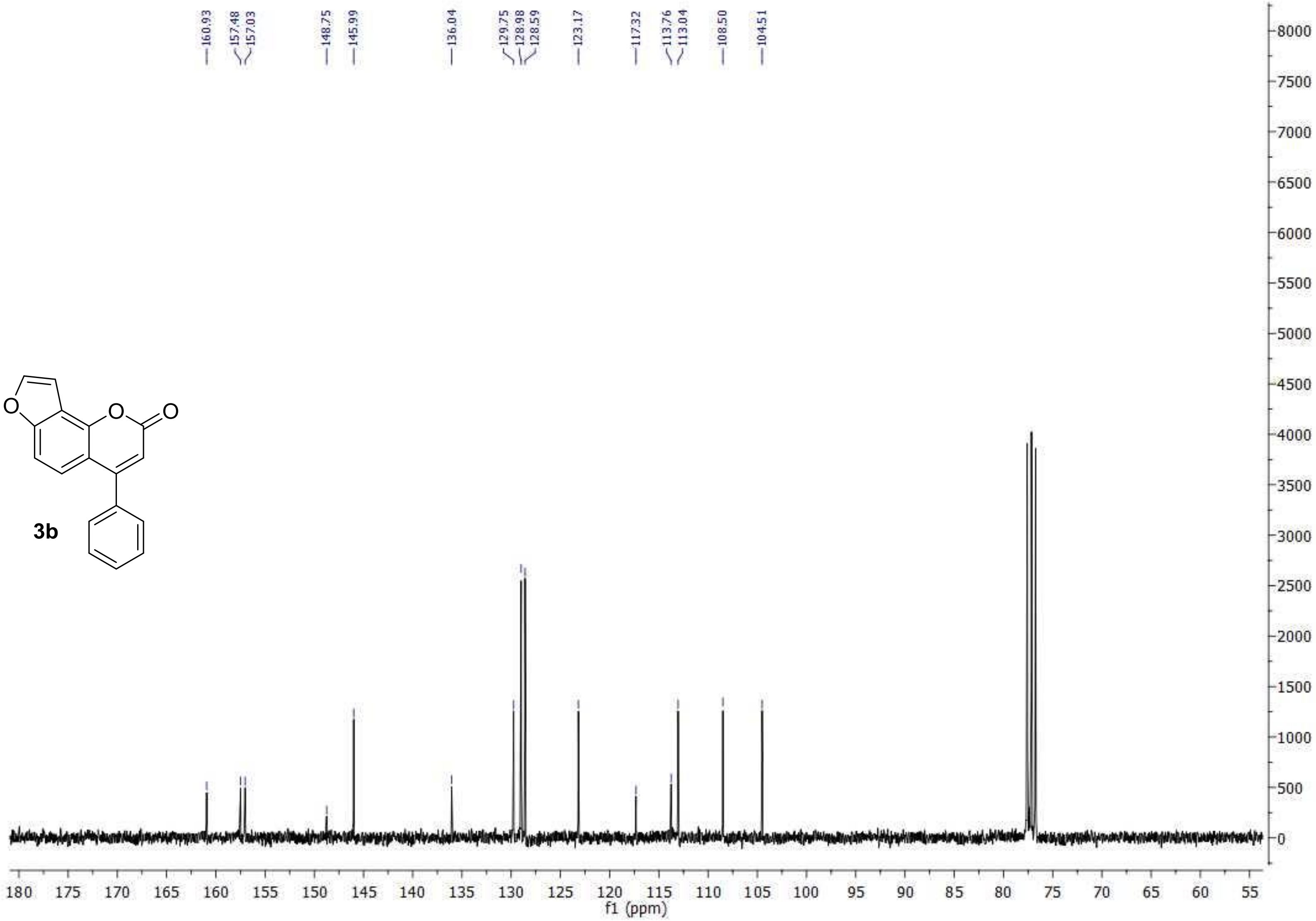

3b

^{1}H NMR (300 MHz, $CDCl_3$) of **3c**

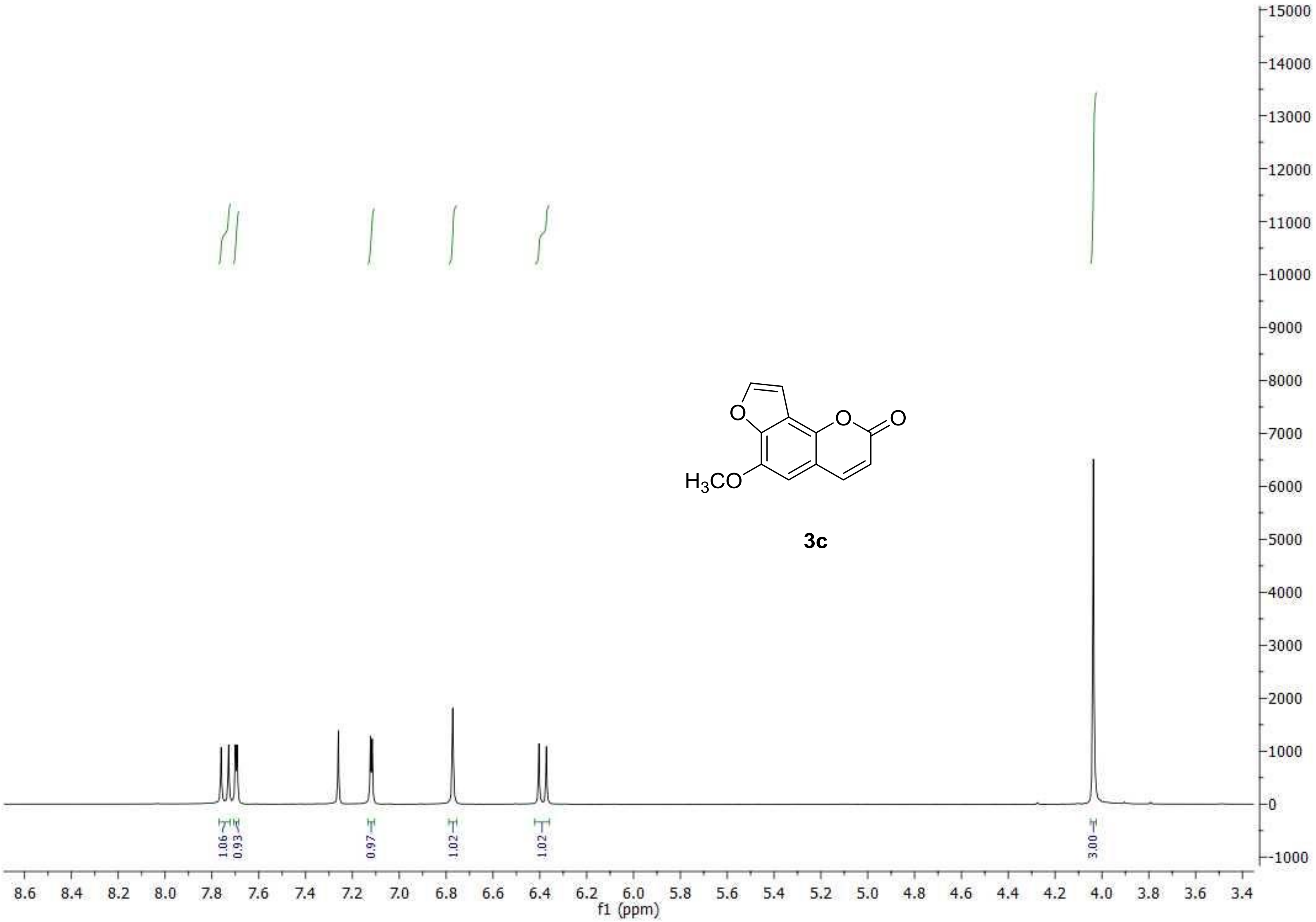

^{13}C NMR (75 MHz, $CDCl_3$) of **3c**

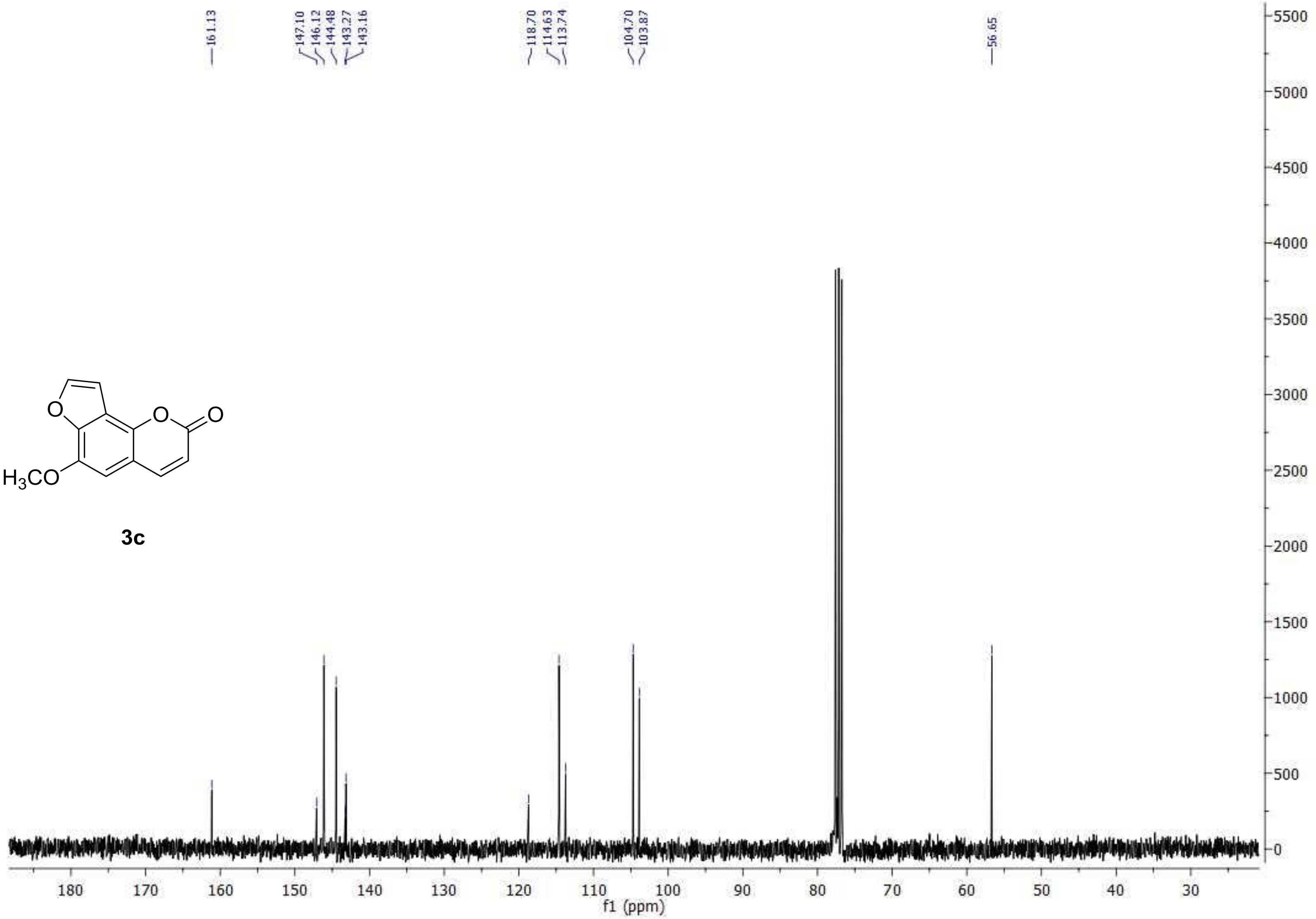

3c

^{1}H NMR (300 MHz, $CDCl_3$) of **3d**

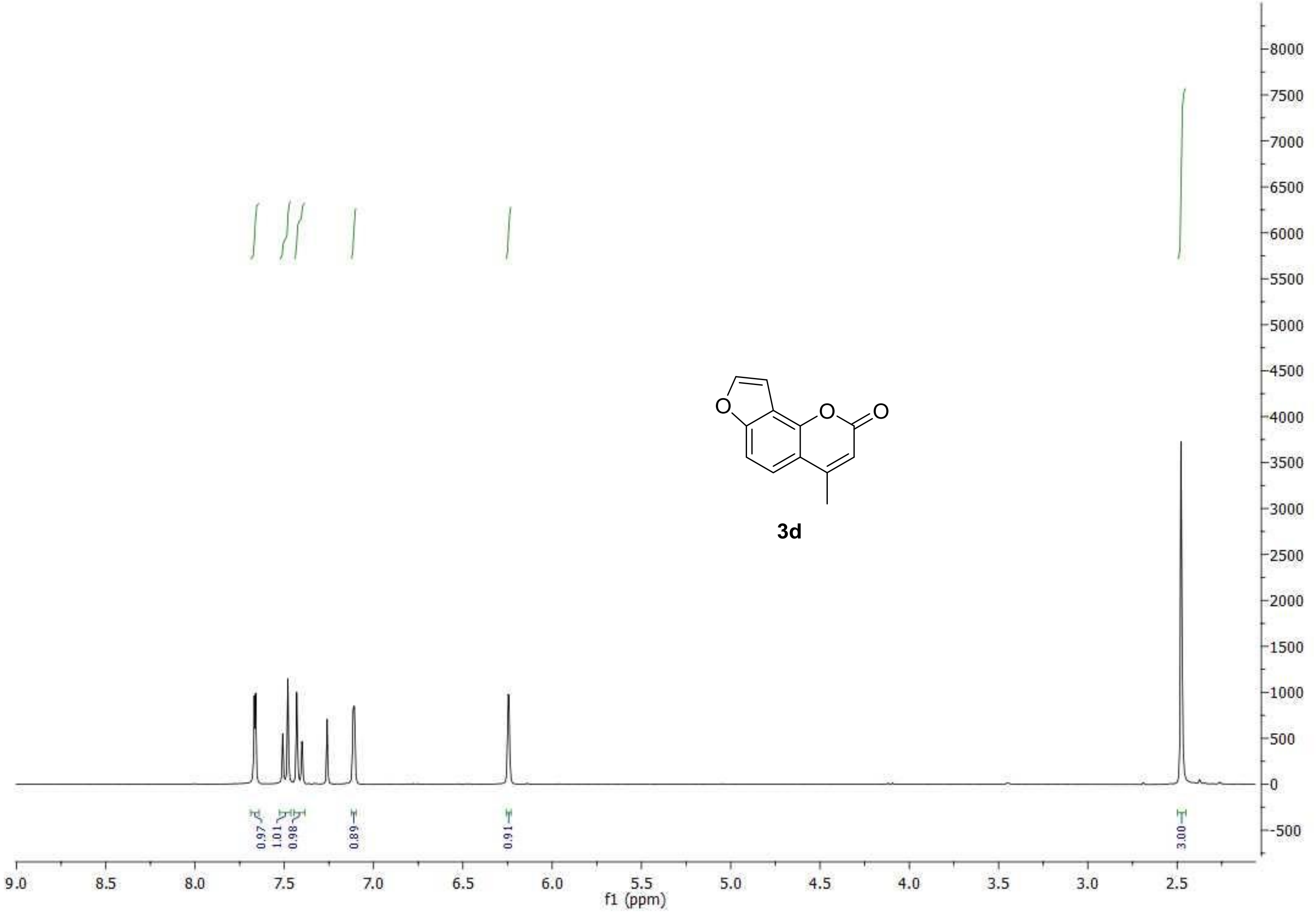

^{13}C NMR (75 MHz, $CDCl_3$) of **3d**

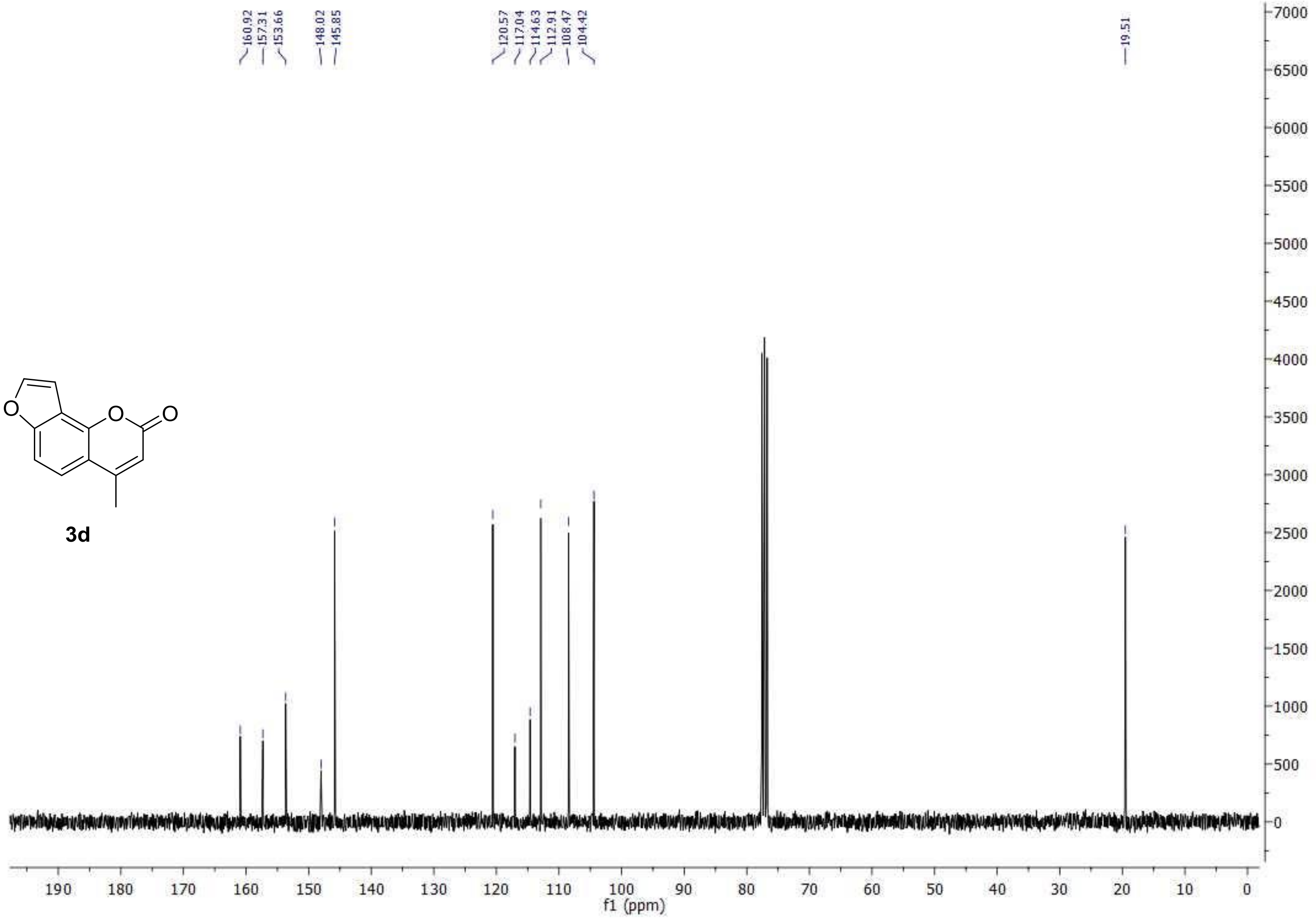

3d

^{1}H NMR (300 MHz, $CDCl_3$) of **12a**

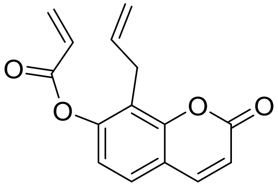

12a

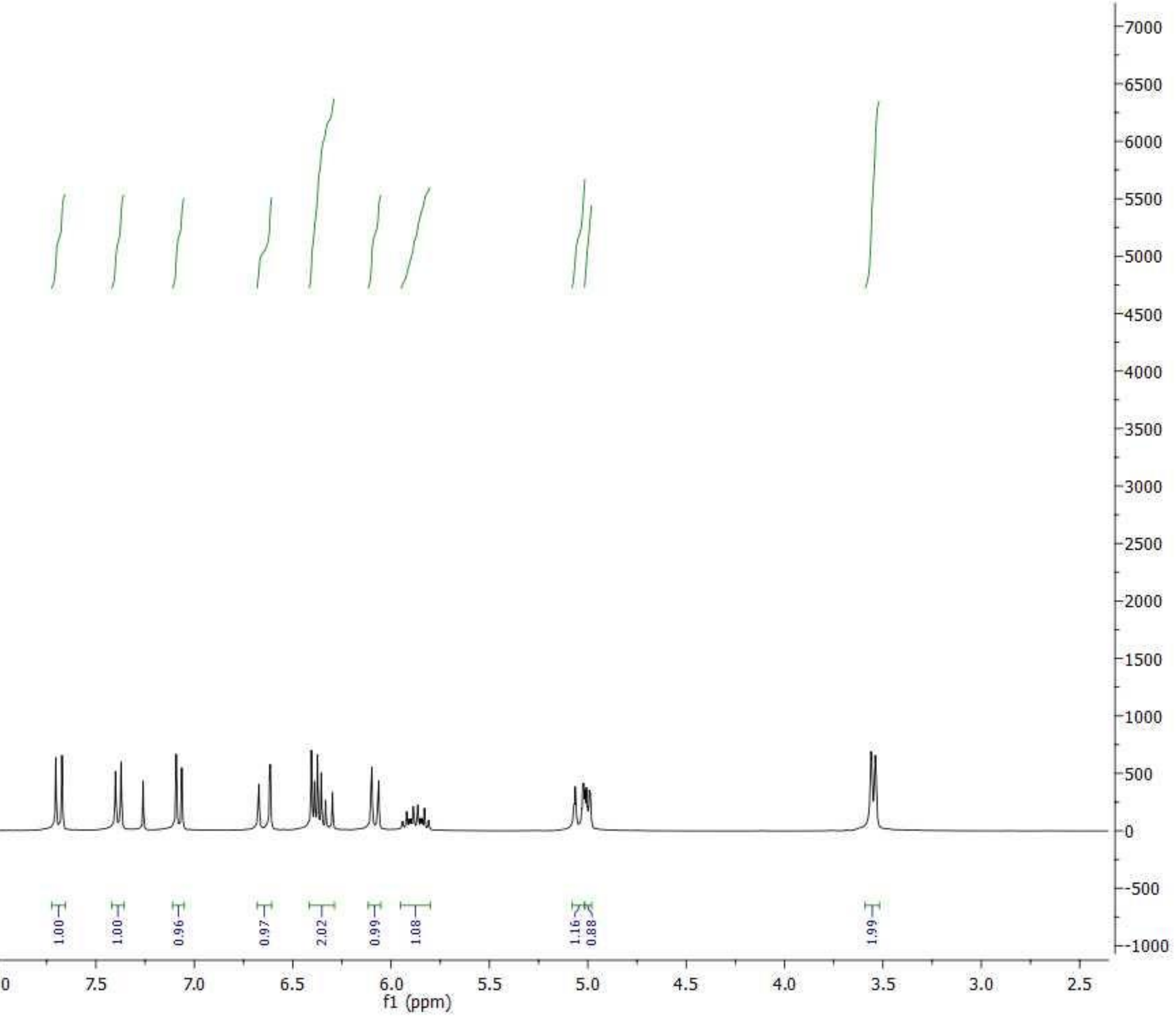

^{13}C NMR (75 MHz, $CDCl_3$) of **12a**

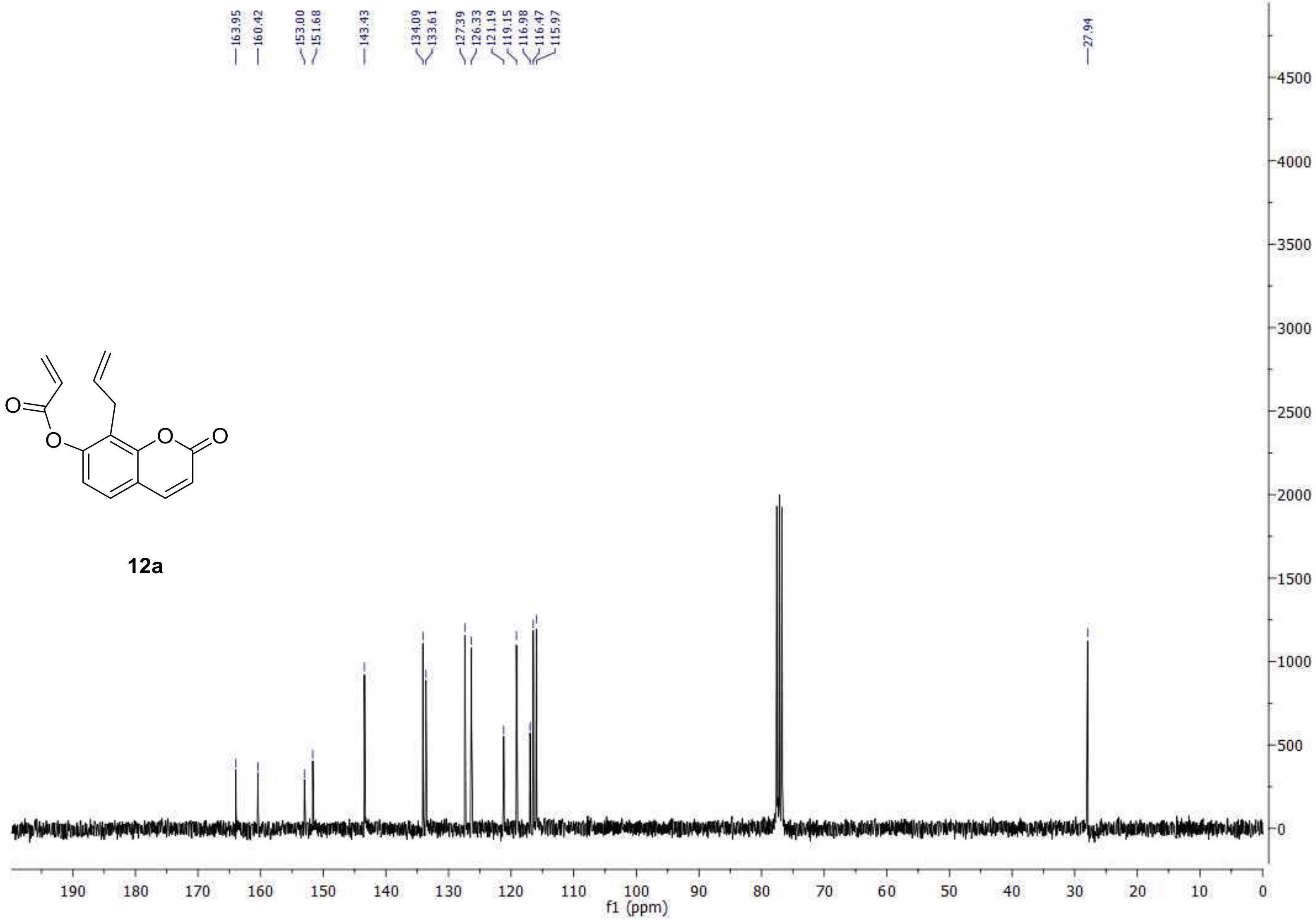

12a

^{1}H NMR (300 MHz, $CDCl_3$) of **12b**

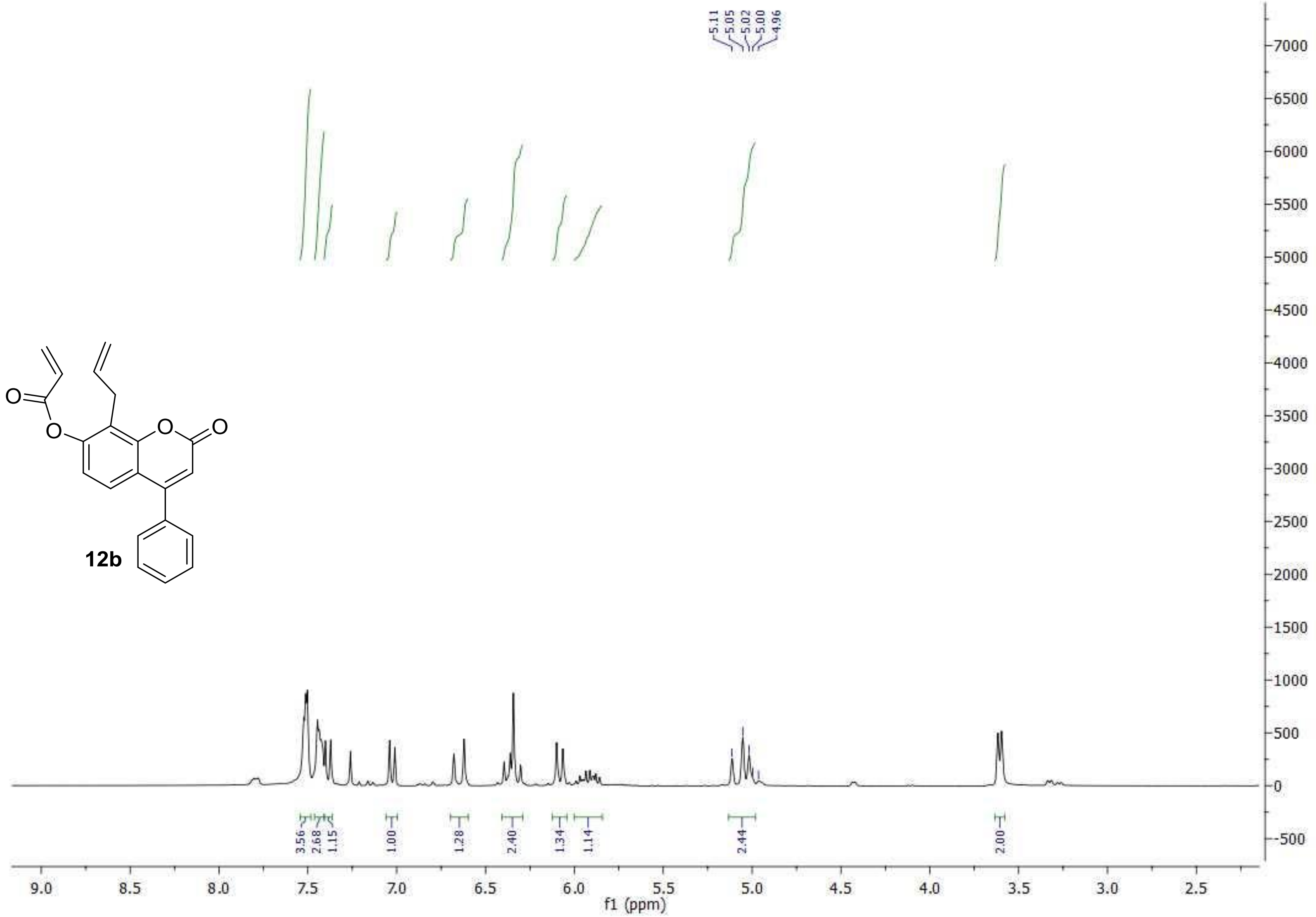

^{13}C NMR (75 MHz, $CDCl_3$) of **12b**

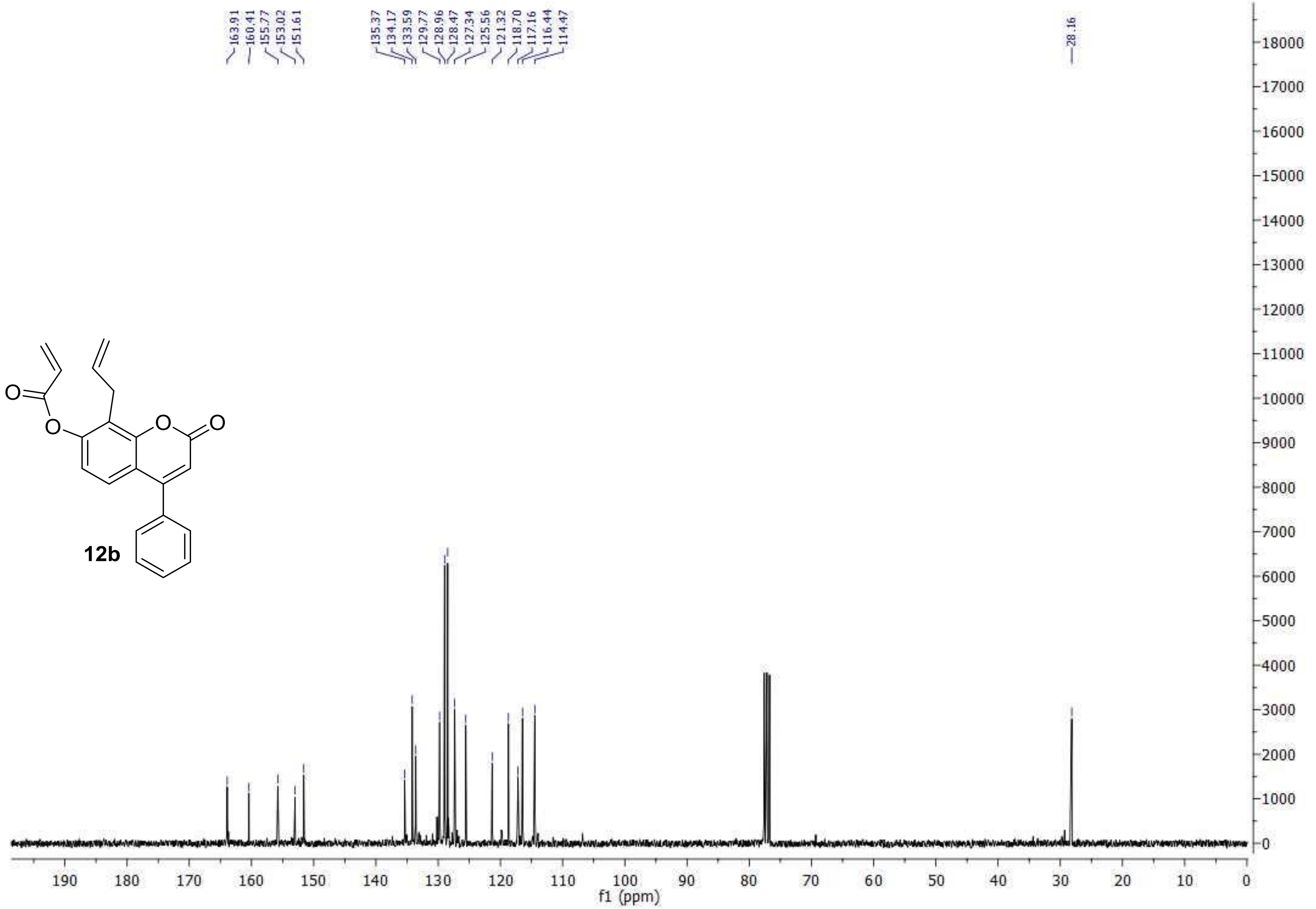

^{1}H NMR (300 MHz, $CDCl_3$) of **12c**

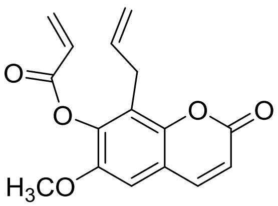

12c

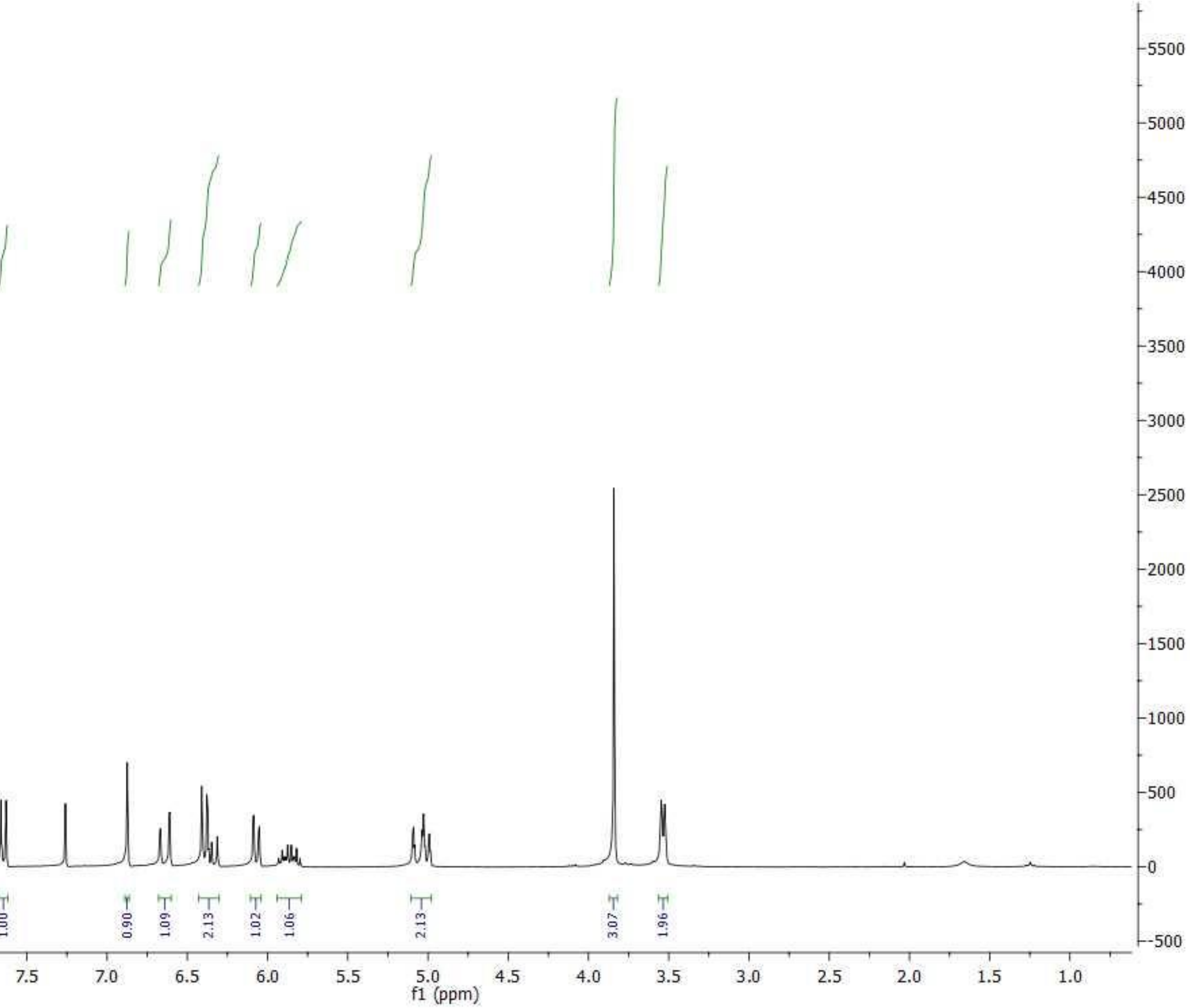

^{13}C NMR (75 MHz, CDCl$_3$) of **12c**

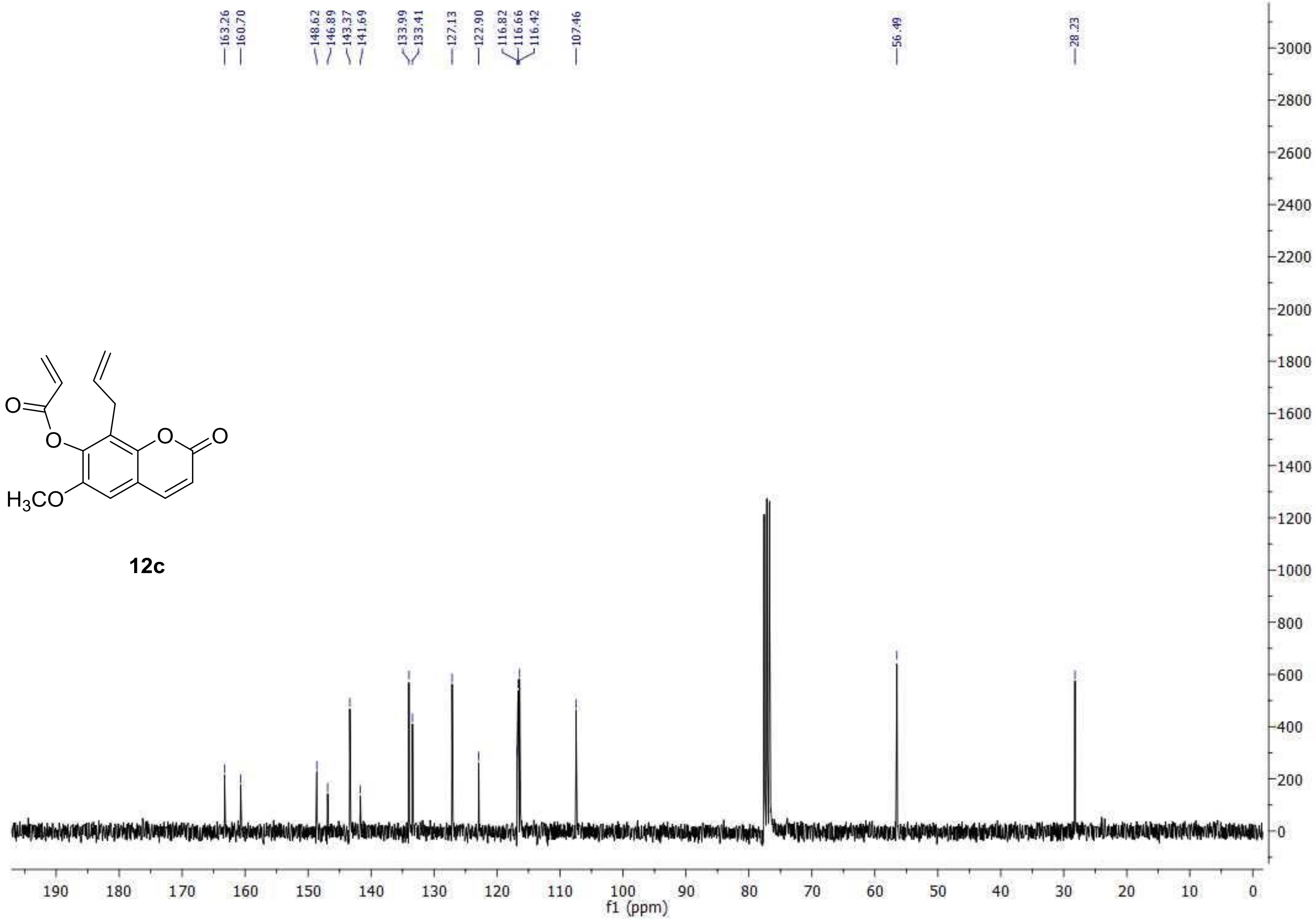

^{1}H NMR (300 MHz, $CDCl_3$) of **12d**

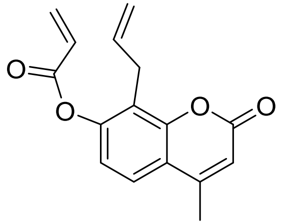

12d

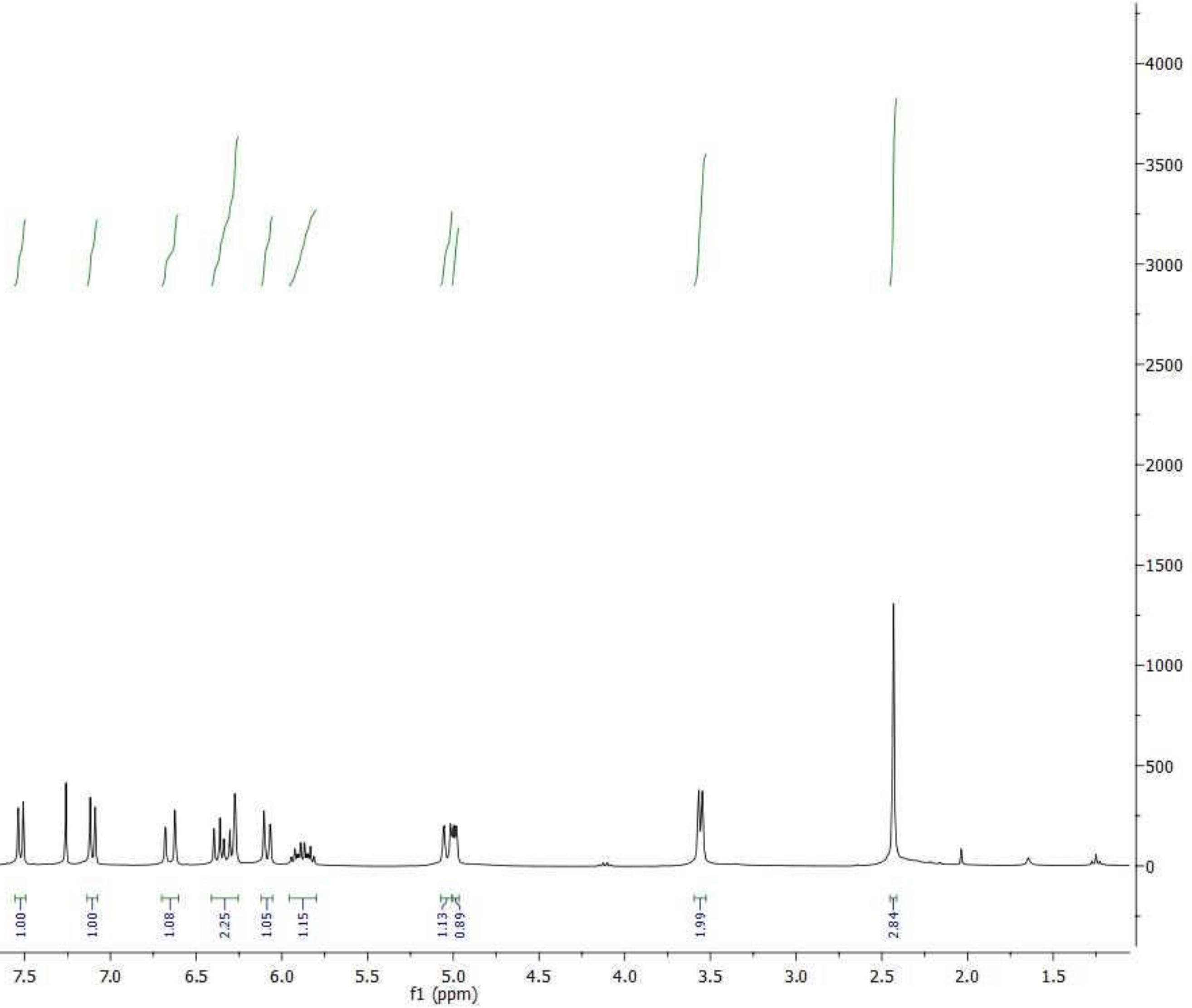

^{13}C NMR (75 MHz, CDCl$_3$) of **12d**

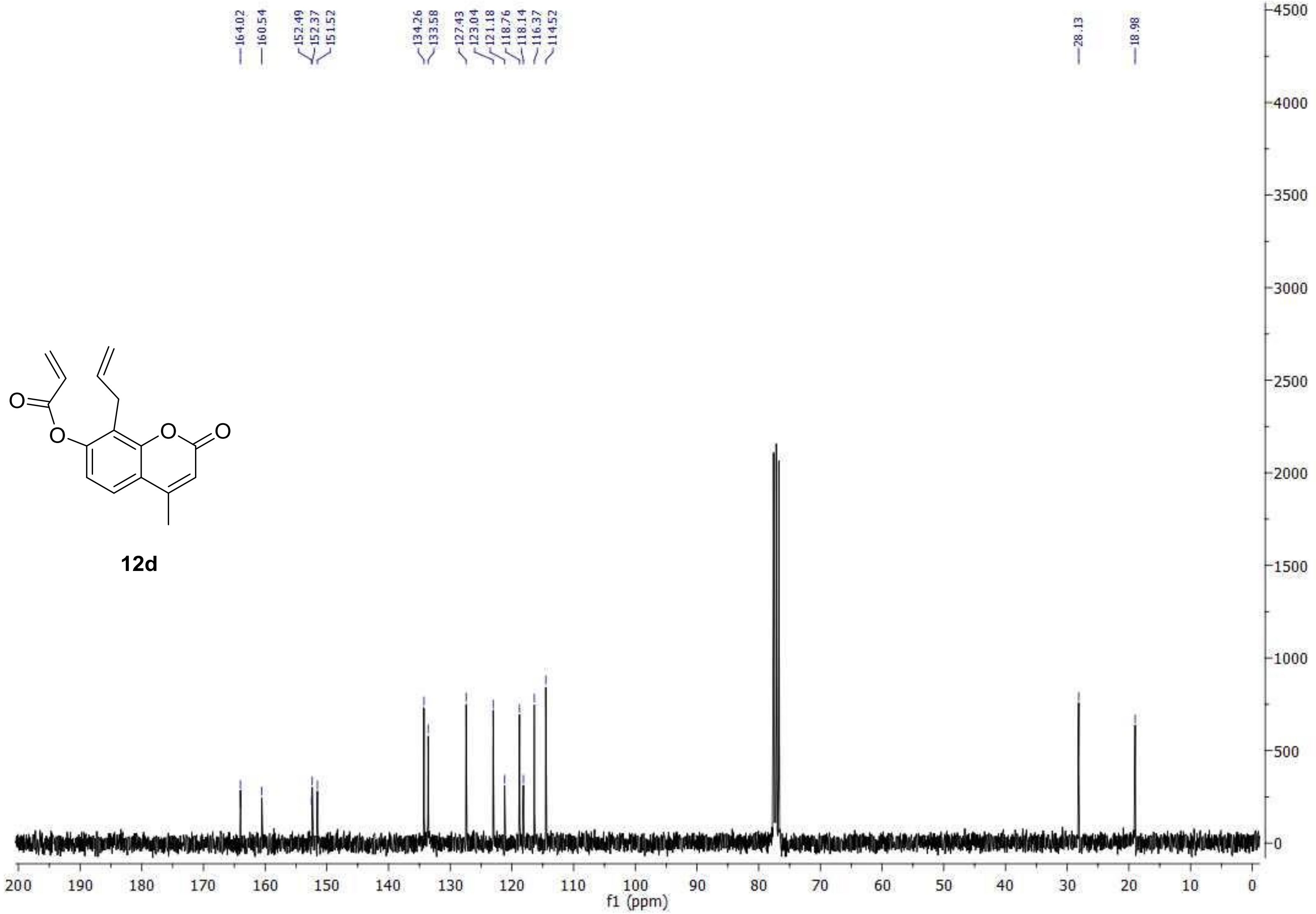

12d

^{1}H NMR (300 MHz, $CDCl_3$) of **13a**

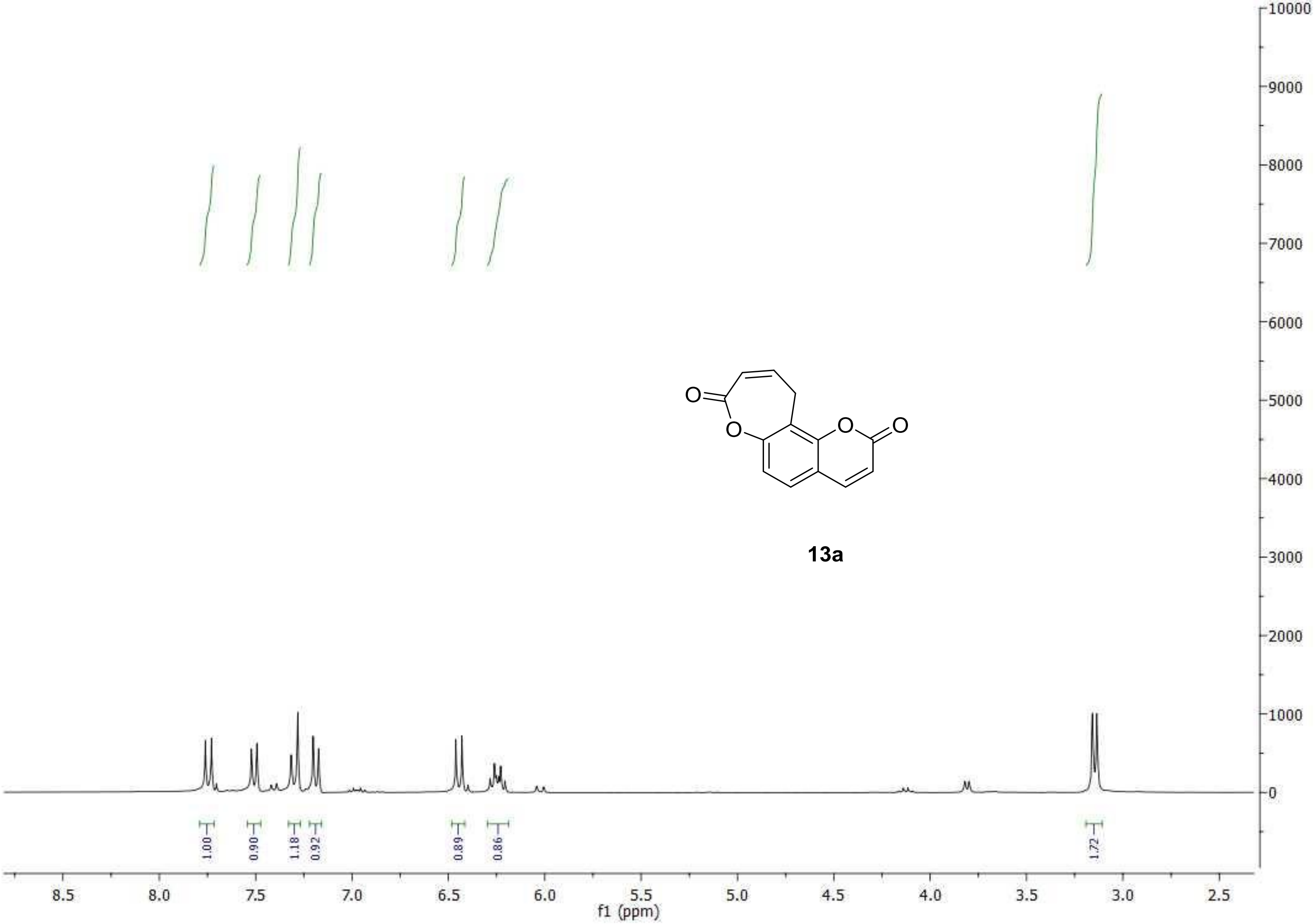

^{13}C NMR (75 MHz, $CDCl_3$) of **13a**

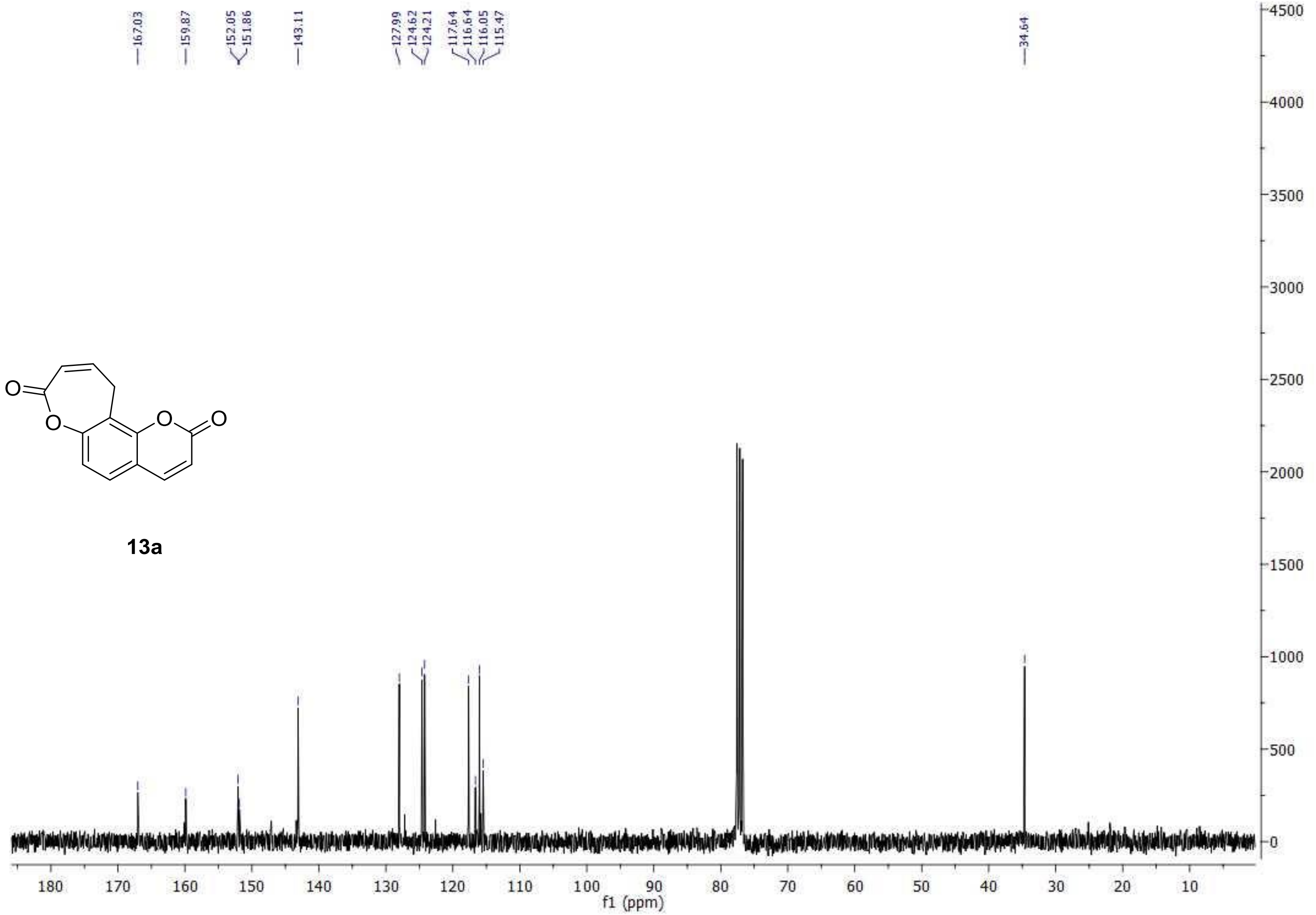

13a

^{1}H NMR (300 MHz, $CDCl_3$) of **13b**

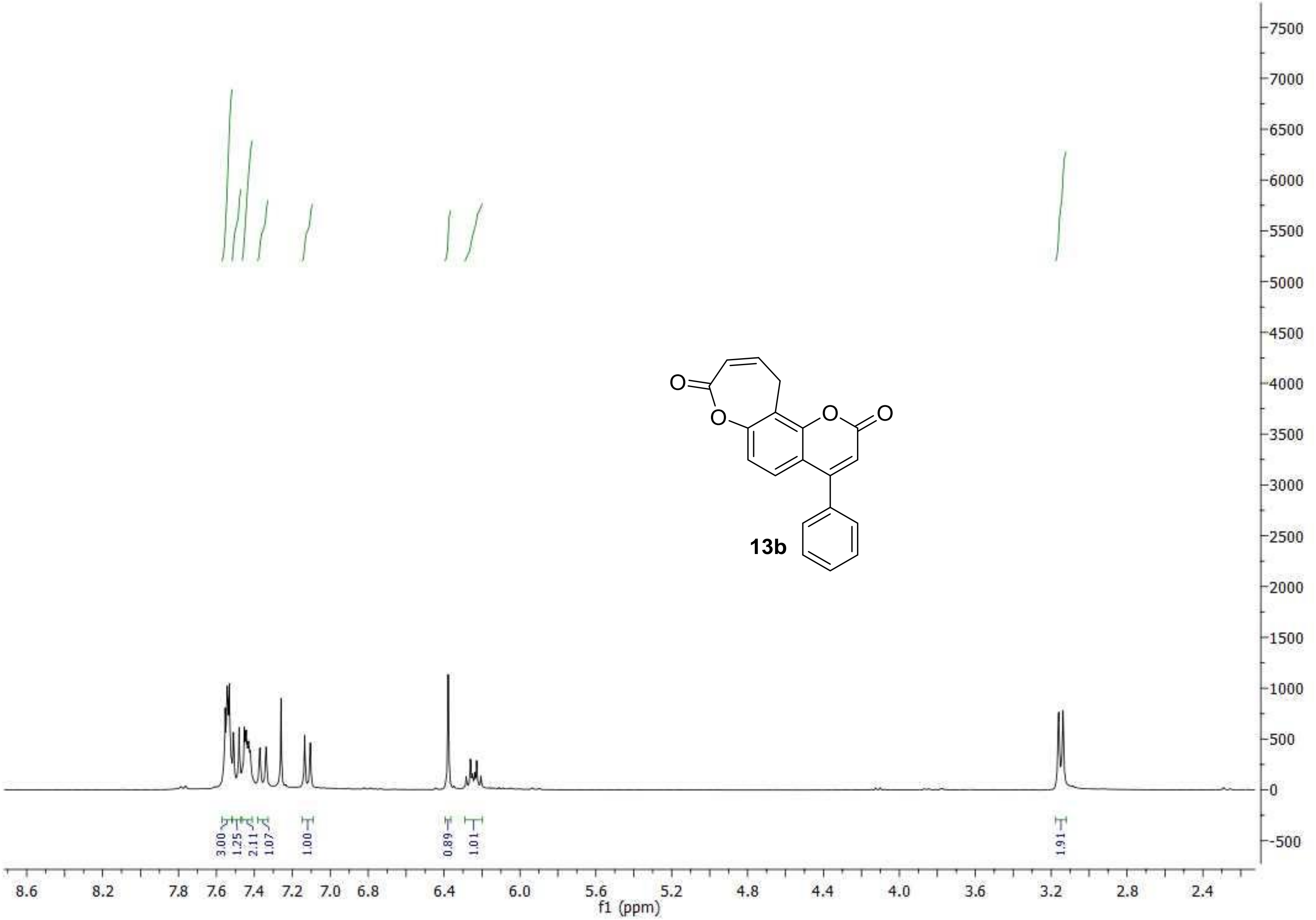

^{13}C NMR (75 MHz, $CDCl_3$) of **13b**

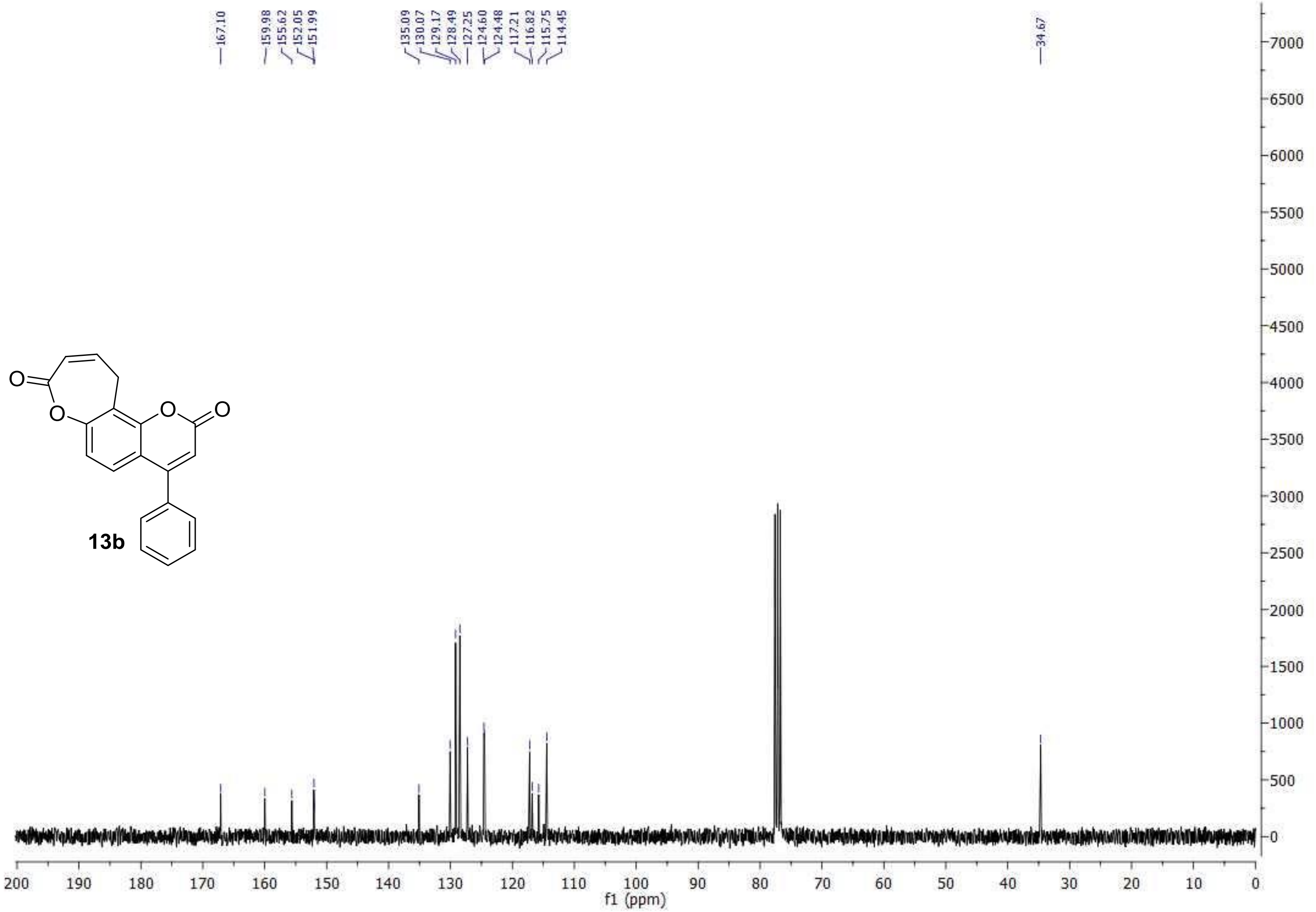

^{1}H NMR (300 MHz, $CDCl_3$) of **13c**

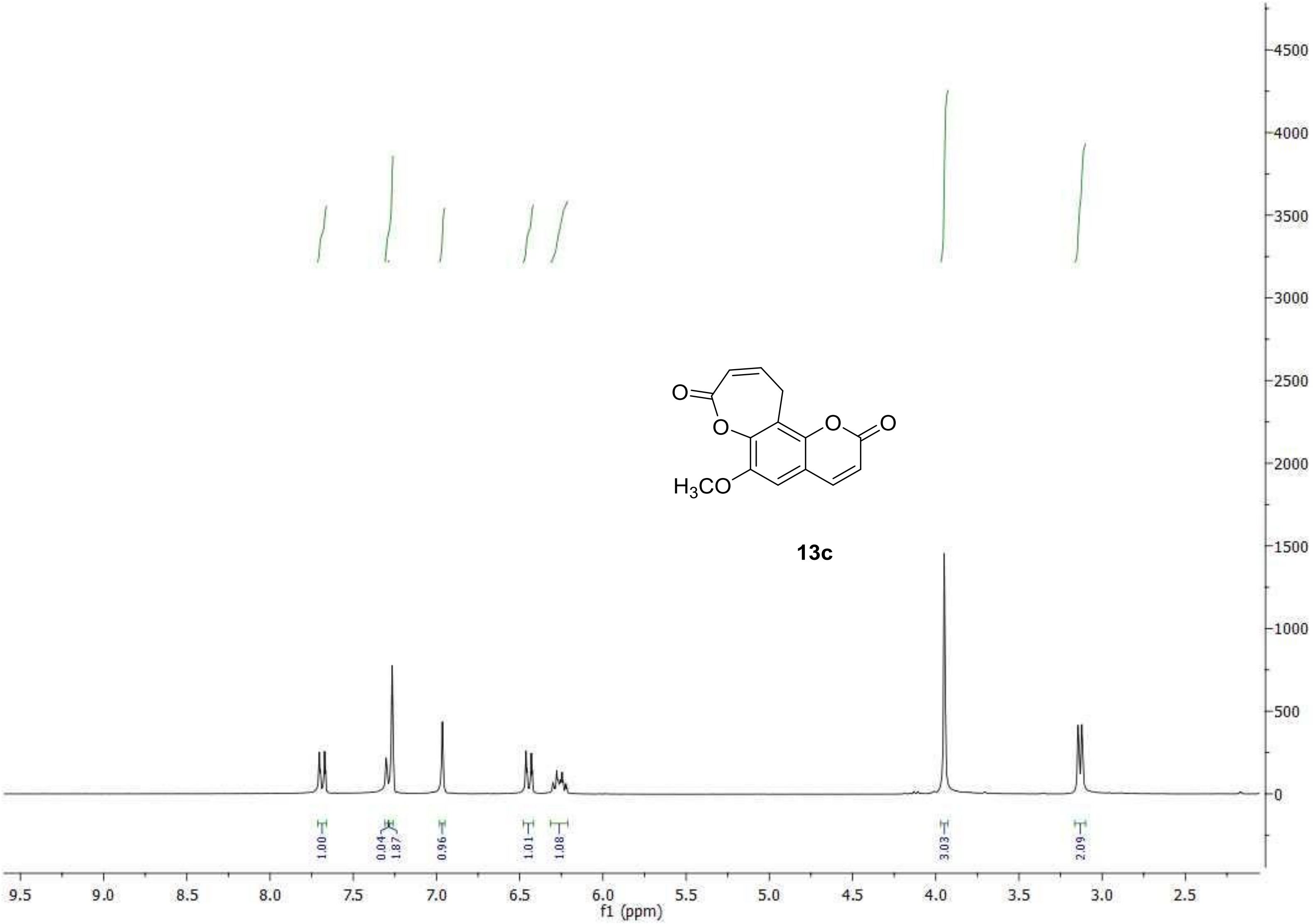

^{13}C NMR (75 MHz, $CDCl_3$) of **13c**

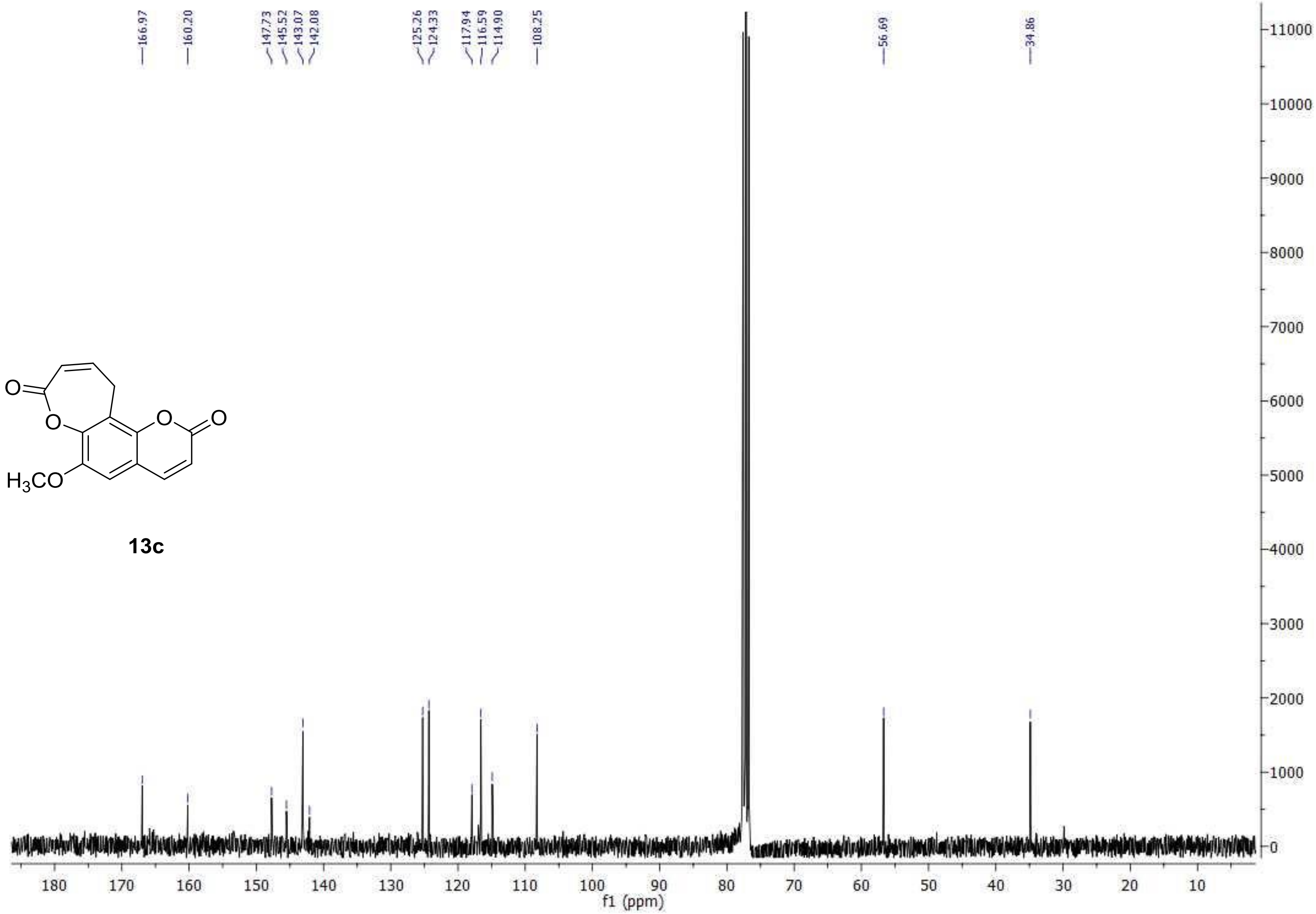

13c

^{1}H NMR (300 MHz, $CDCl_3$) of **13d**

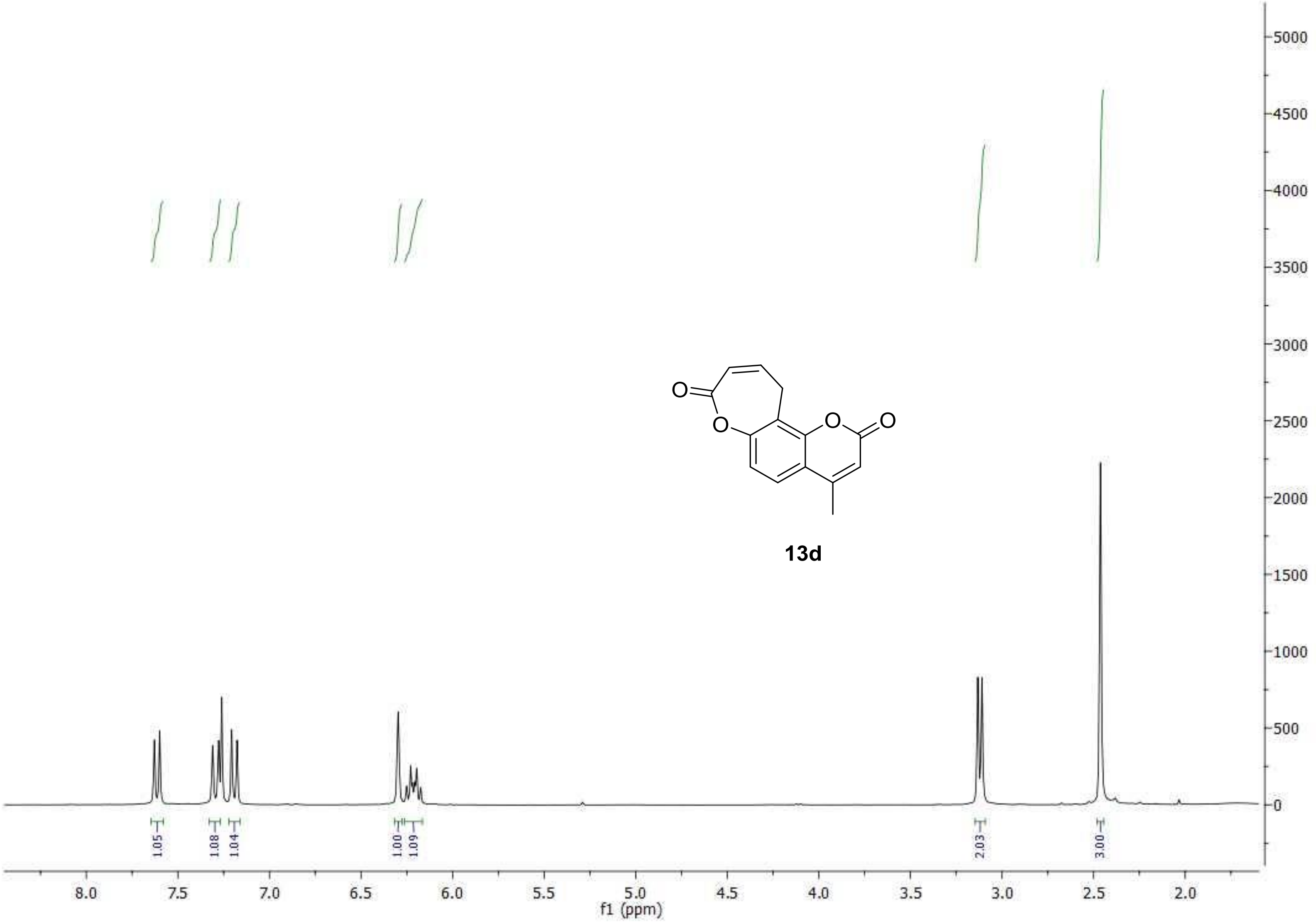

^{13}C NMR (75 MHz, $CDCl_3$) of **13d**

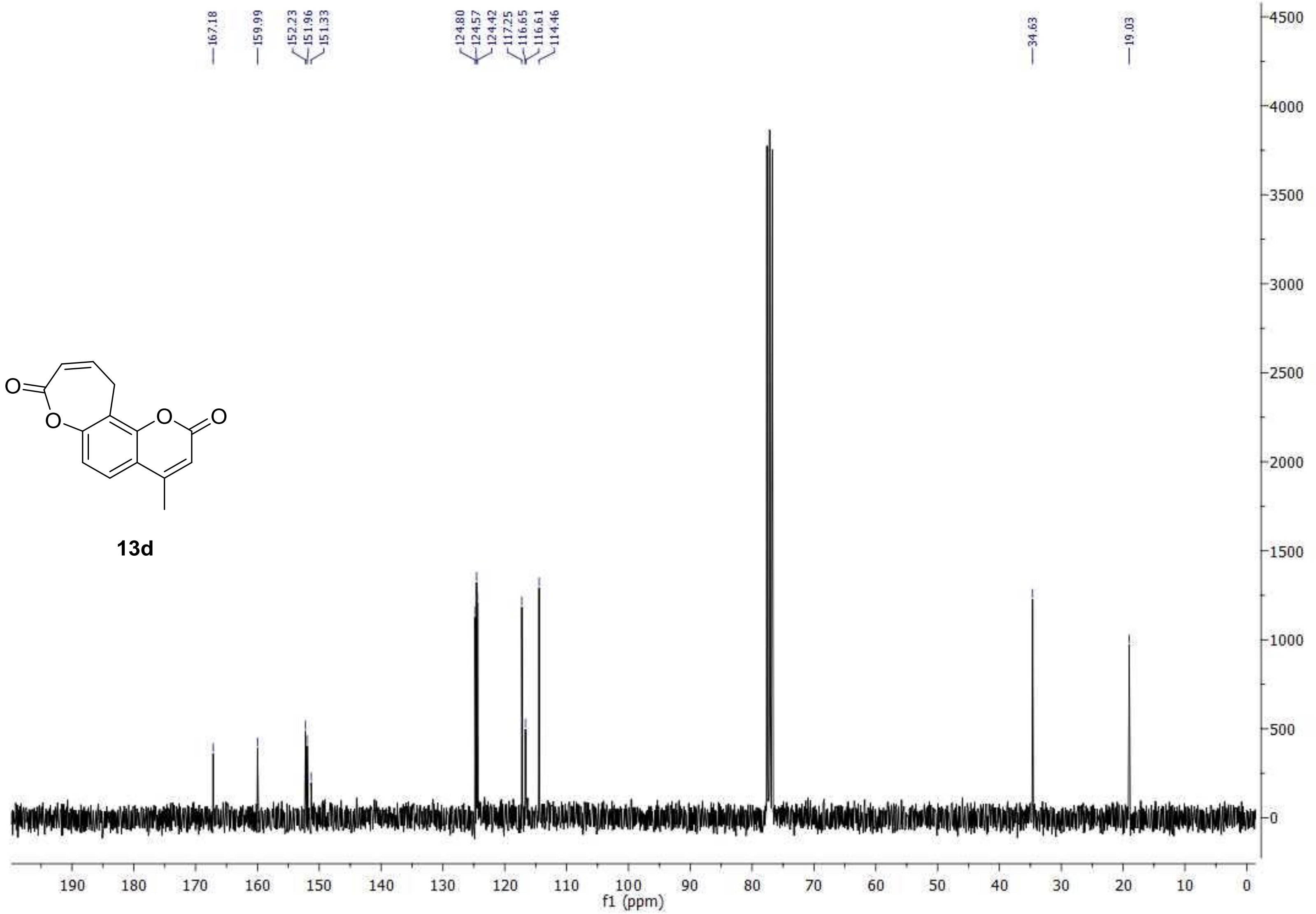

13d

^{1}H NMR (300 MHz, $CDCl_3$) of **14d**

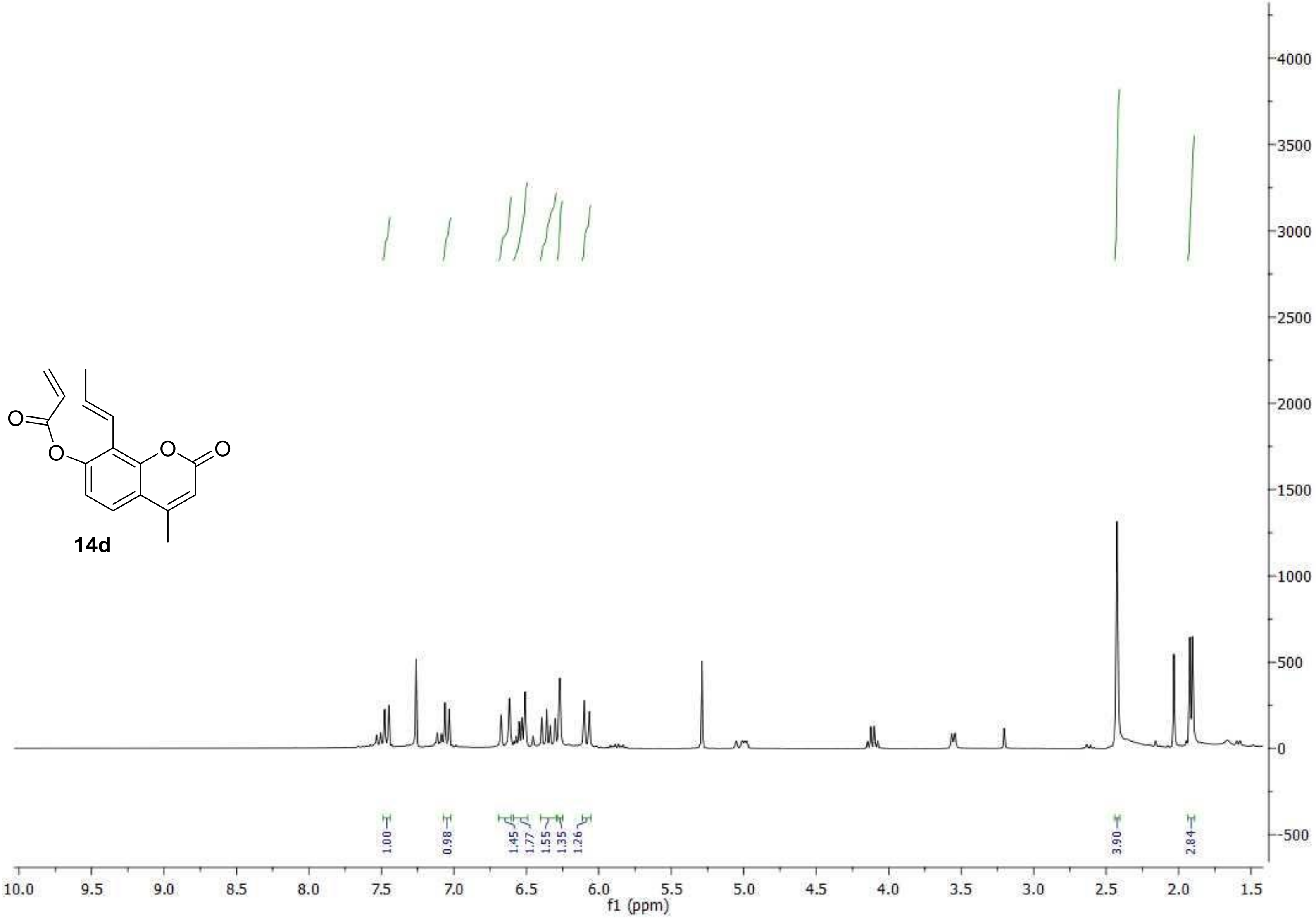

^{13}C NMR (75 MHz, $CDCl_3$) of **14d**

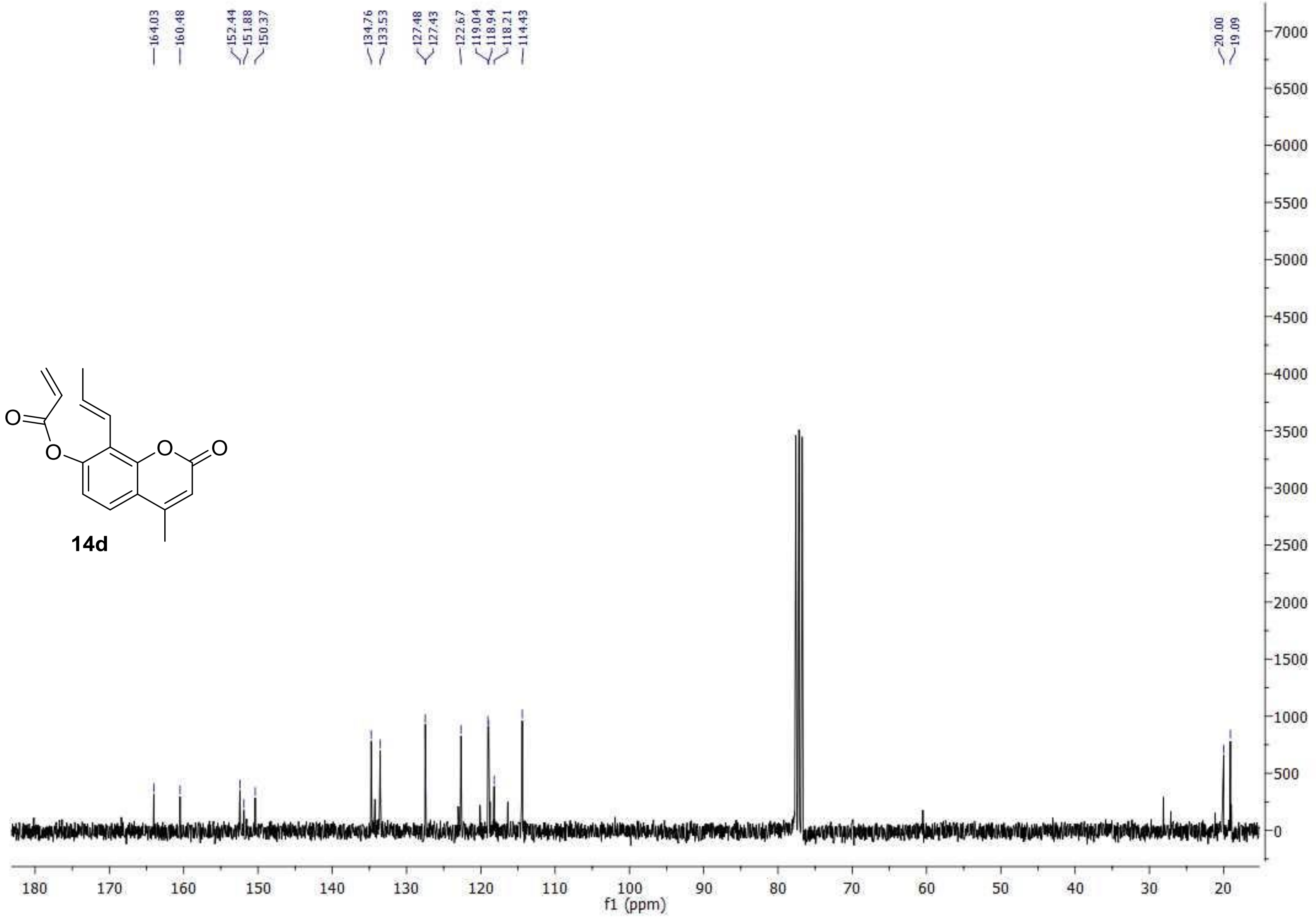

^{1}H NMR (300 MHz, acetone-d_6) of **16a**

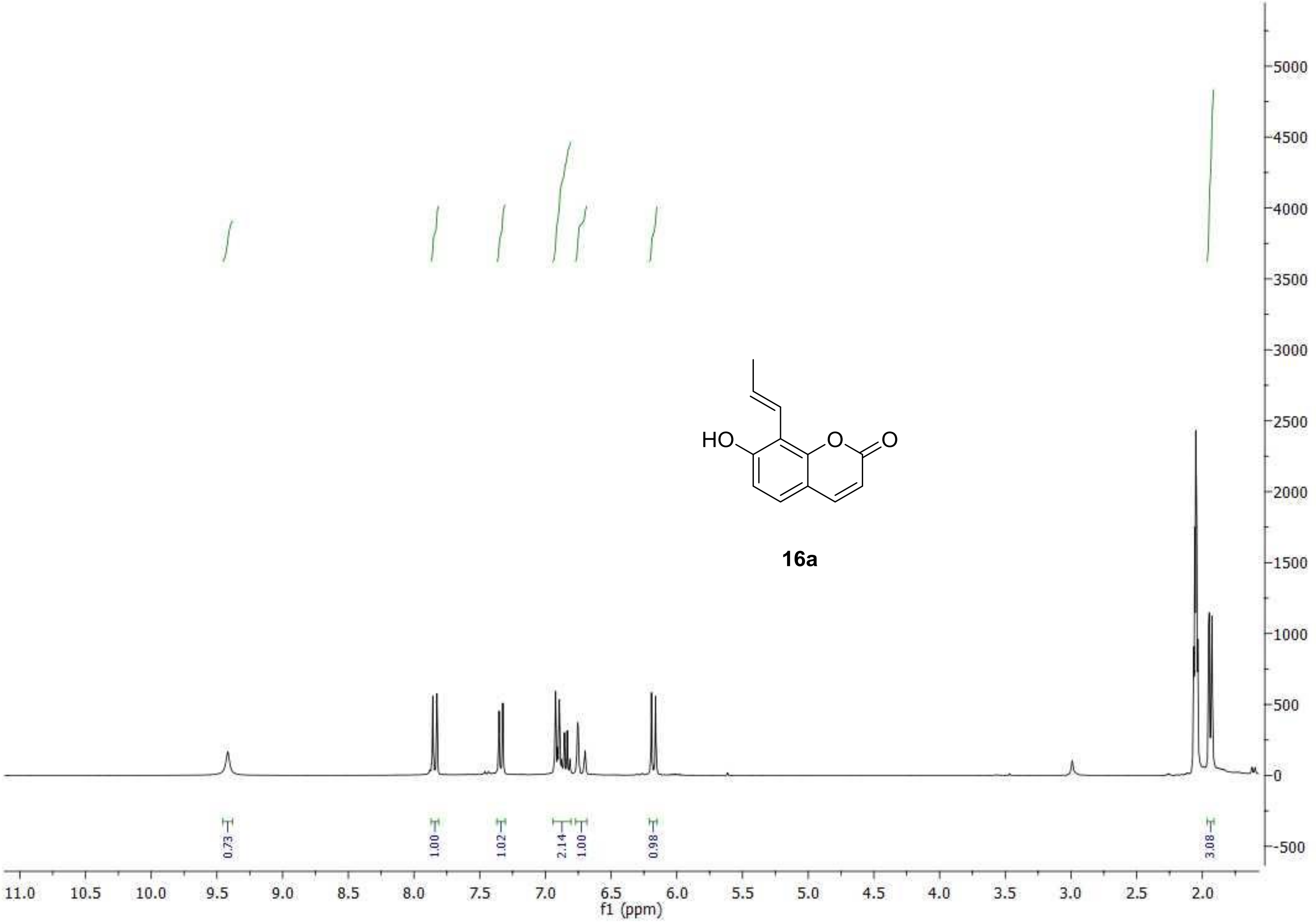

^{13}C NMR (75 MHz, acetone-d_6) of **16a**

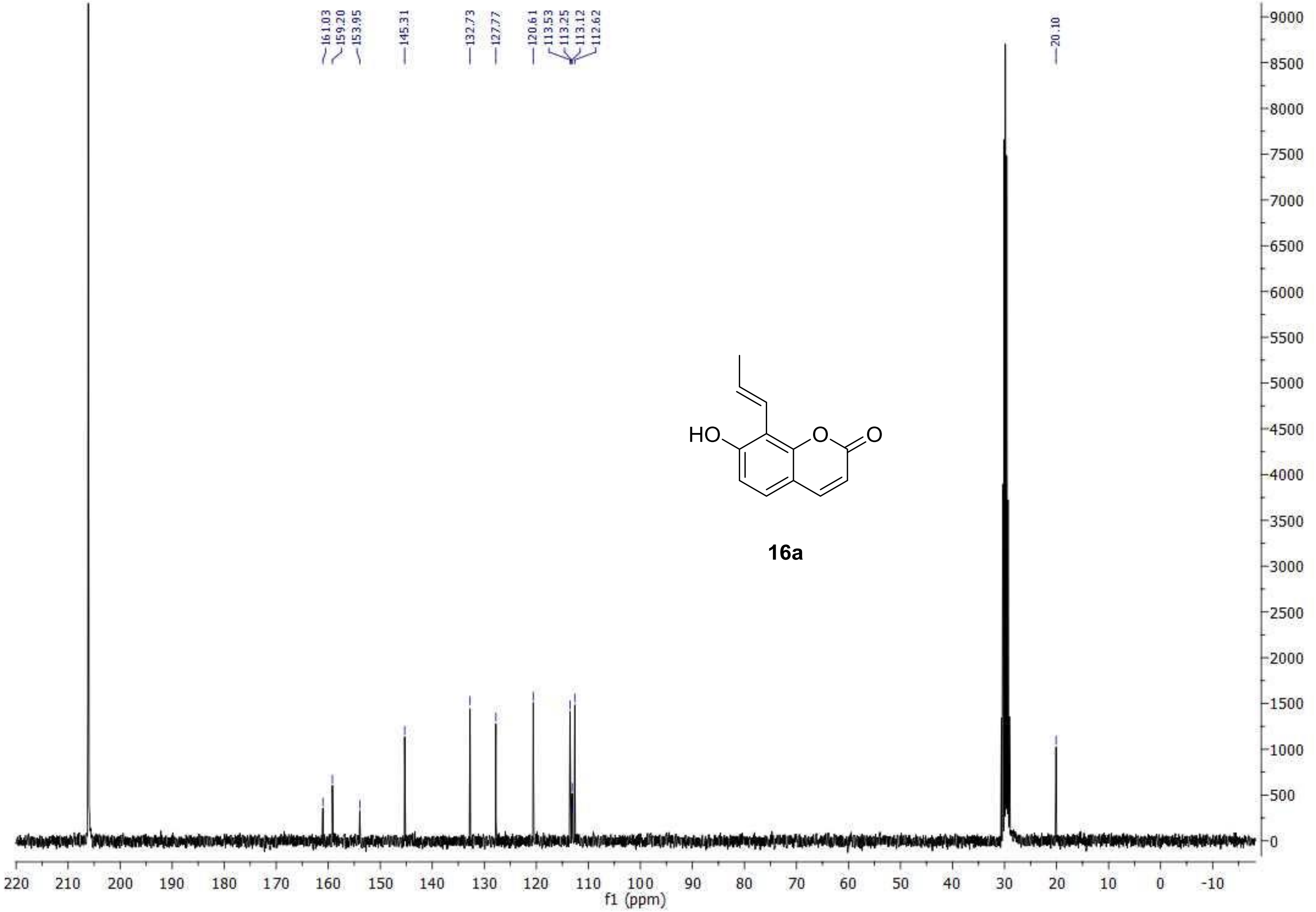

^{1}H NMR (300 MHz, DMSO-d_6) of **16b**

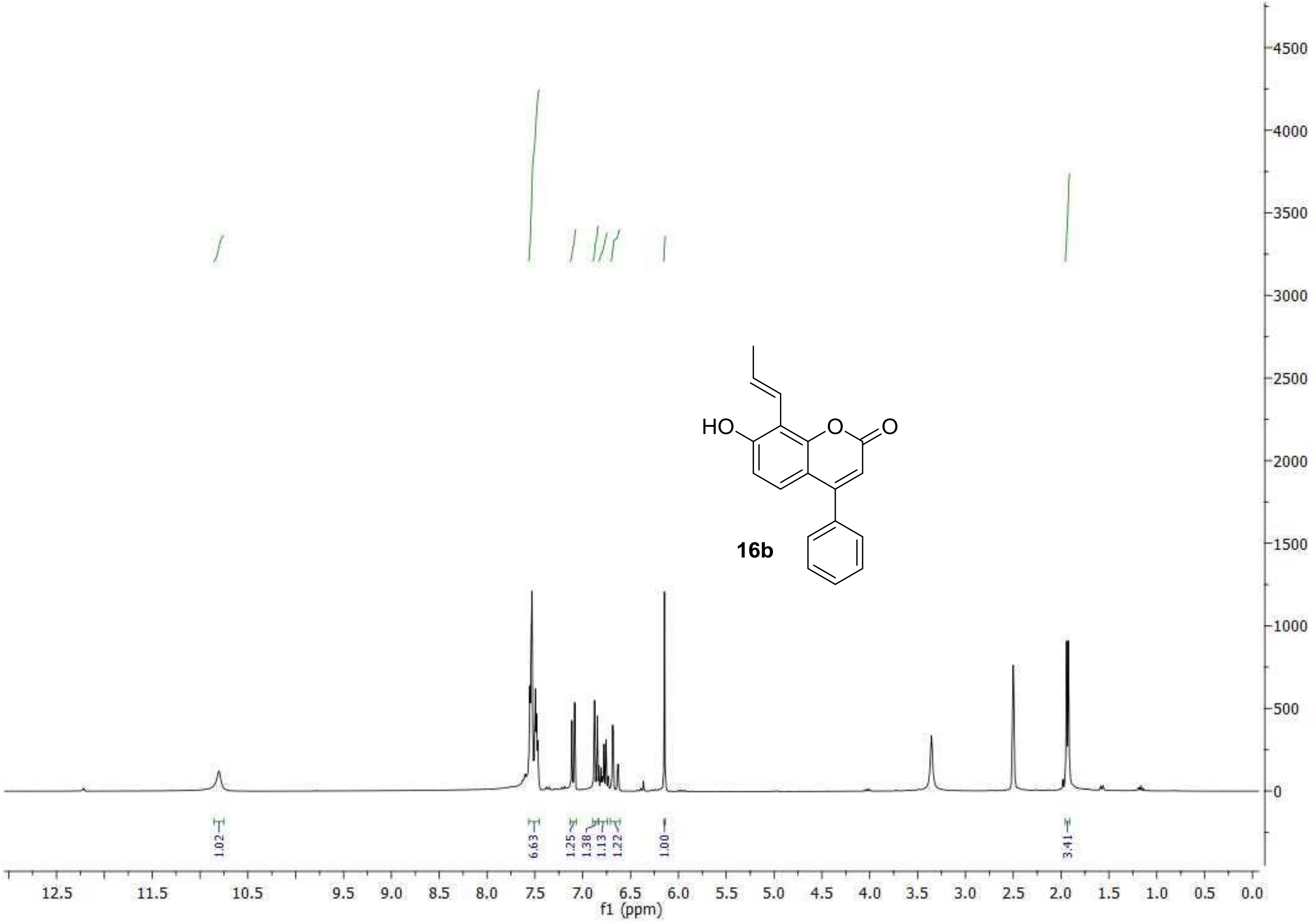

^{13}C NMR-APT (75 MHz, DMSO-d_6) of **16b**

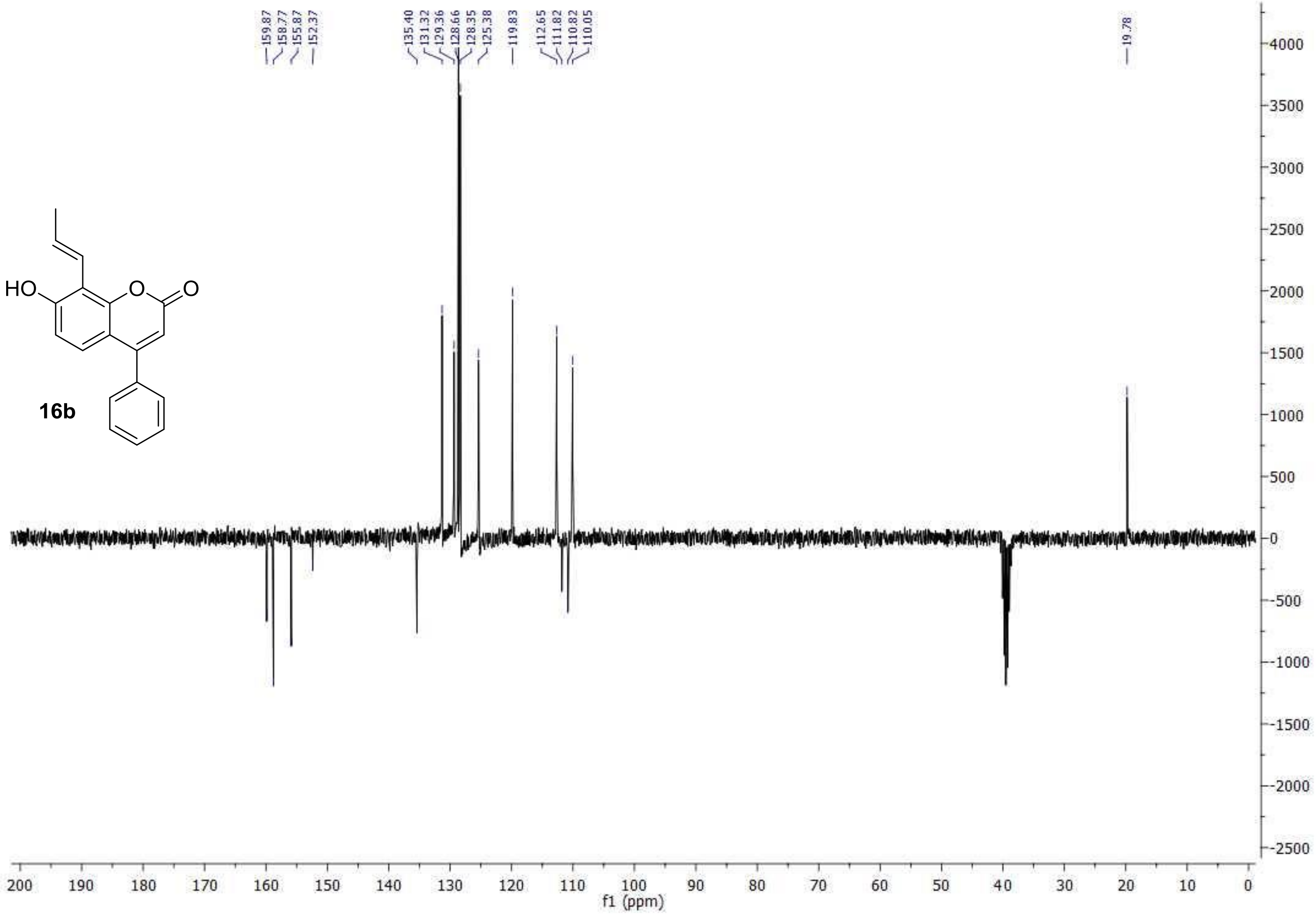

^{1}H NMR (300 MHz, DMSO-d_6) of **16c**

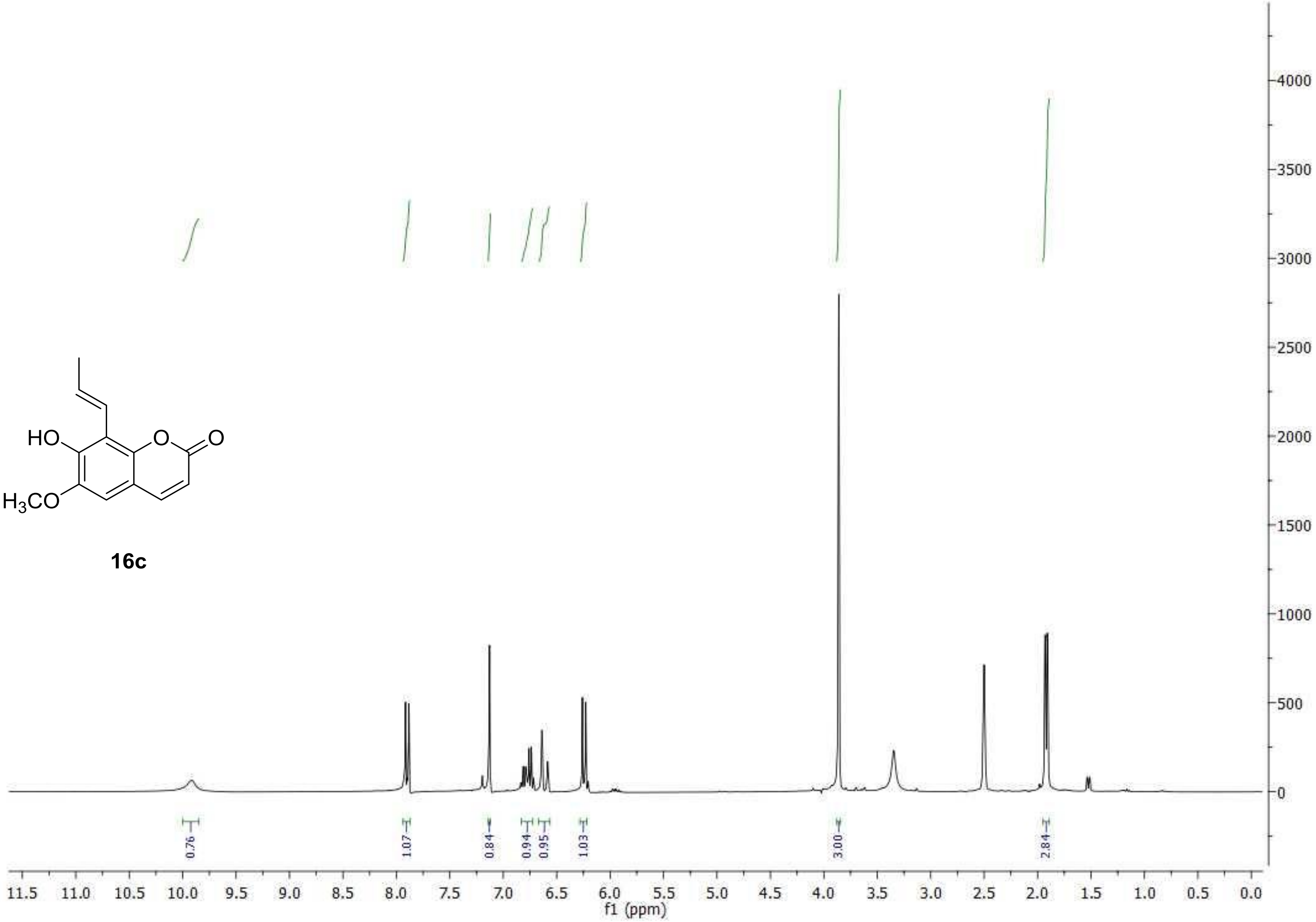

16c

^{13}C NMR-APT (75 MHz, DMSO-d_6) of **16c**

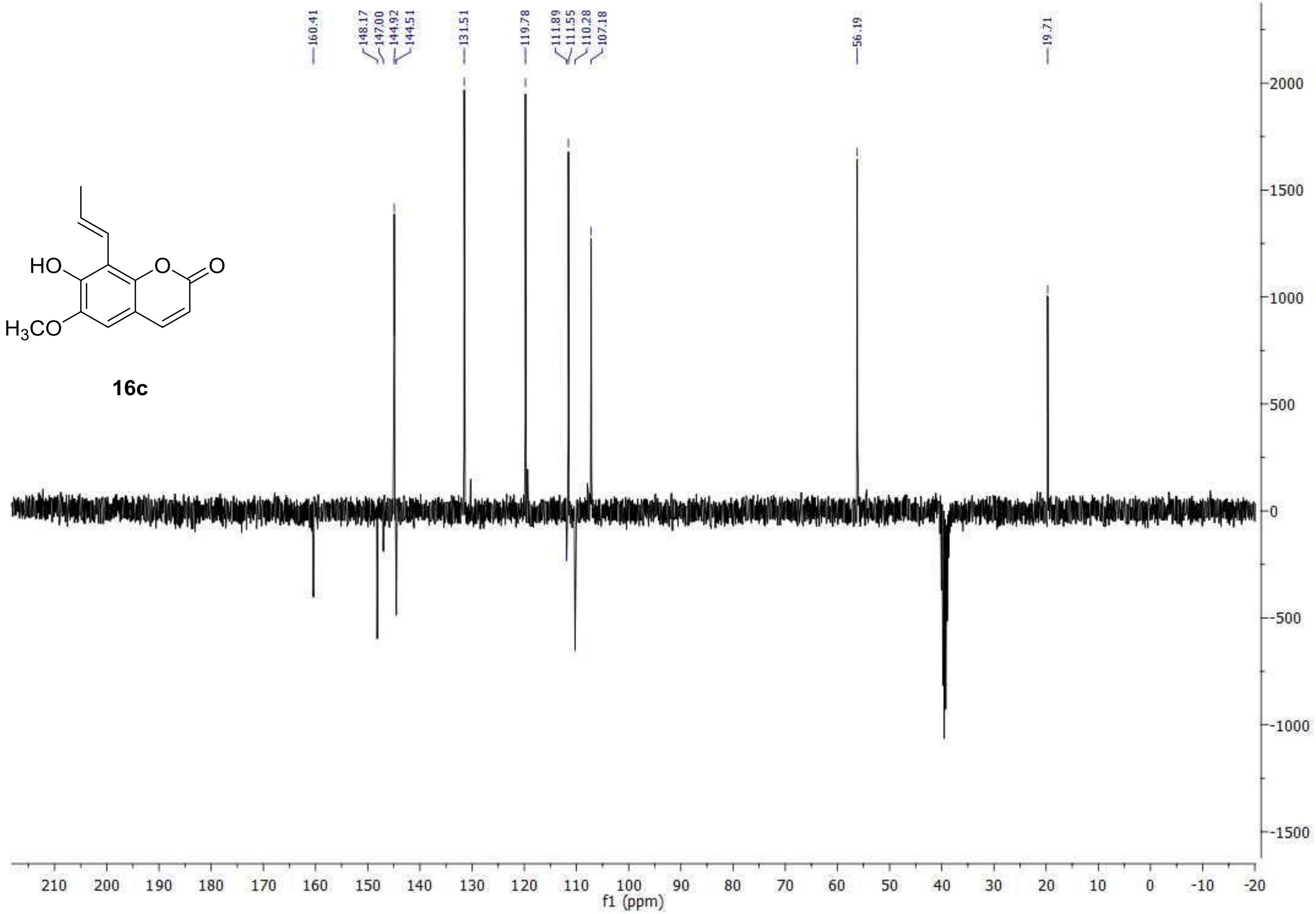

^{1}H NMR (300 MHz, DMSO-d_6) of **16d**

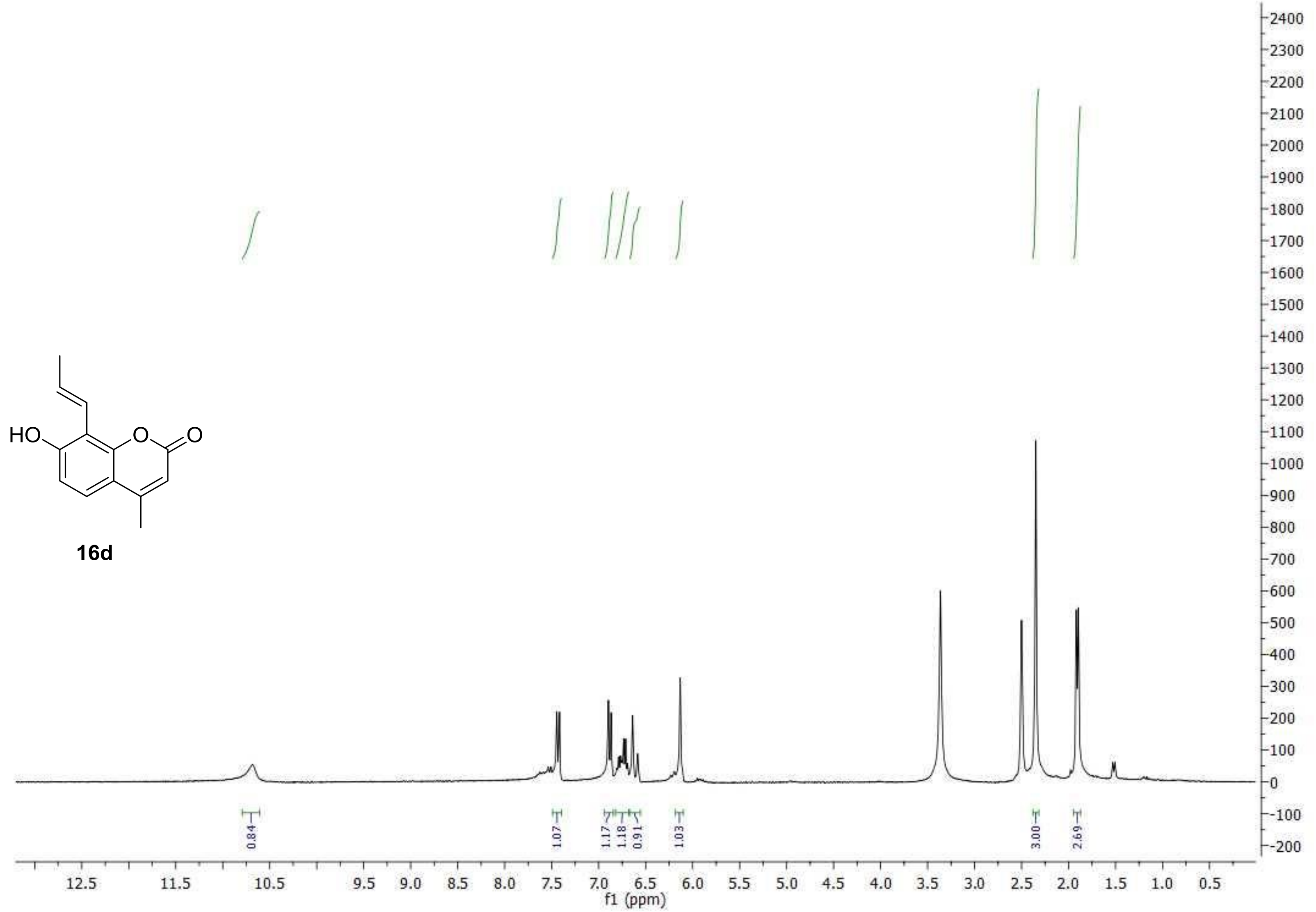

^{13}C NMR (75 MHz, DMSO-d_6) of **16d**

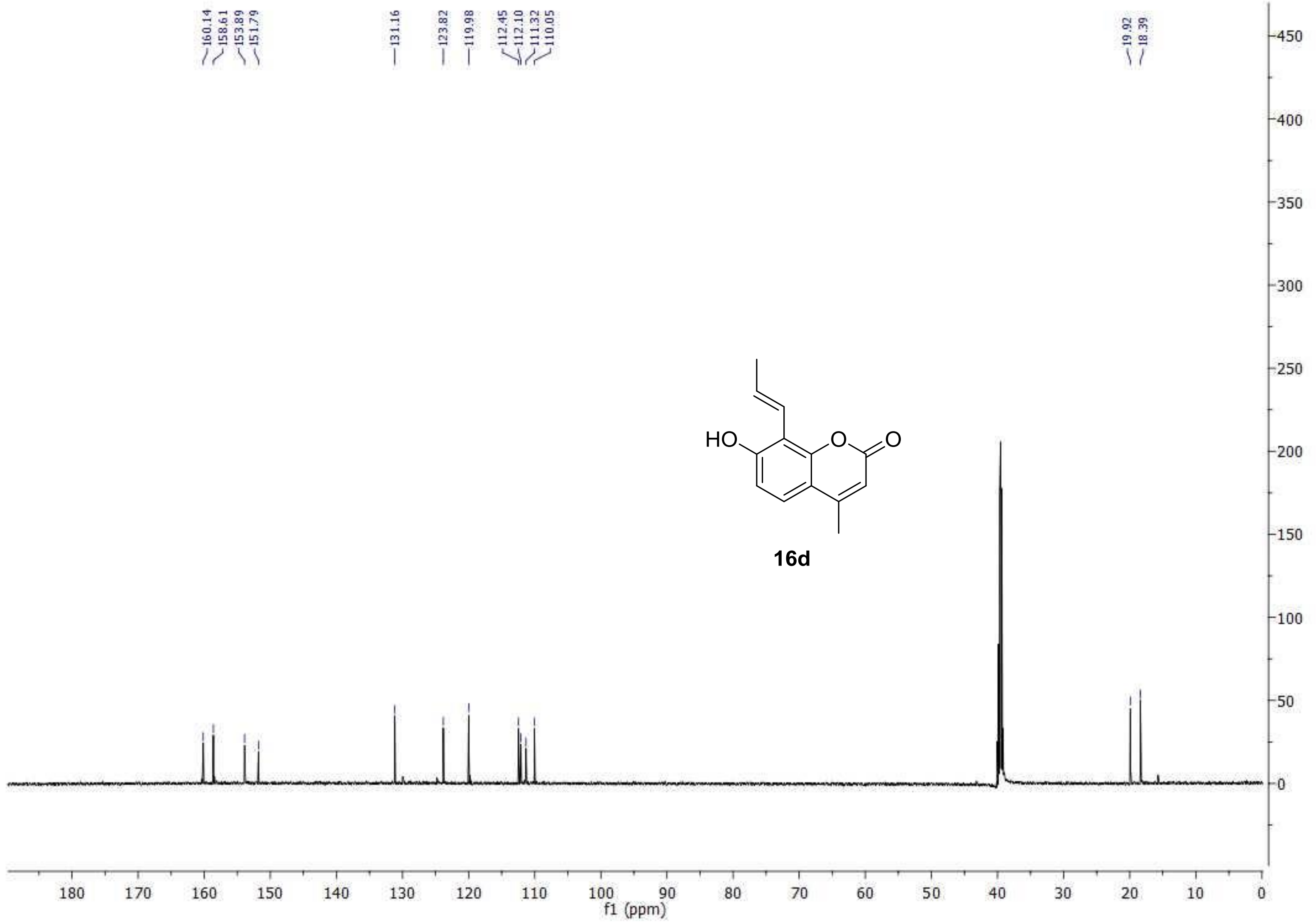

^{1}H NMR (300 MHz, $CDCl_3$) of **17a**

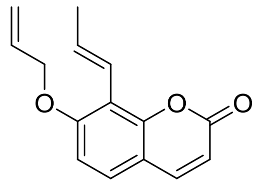

17a

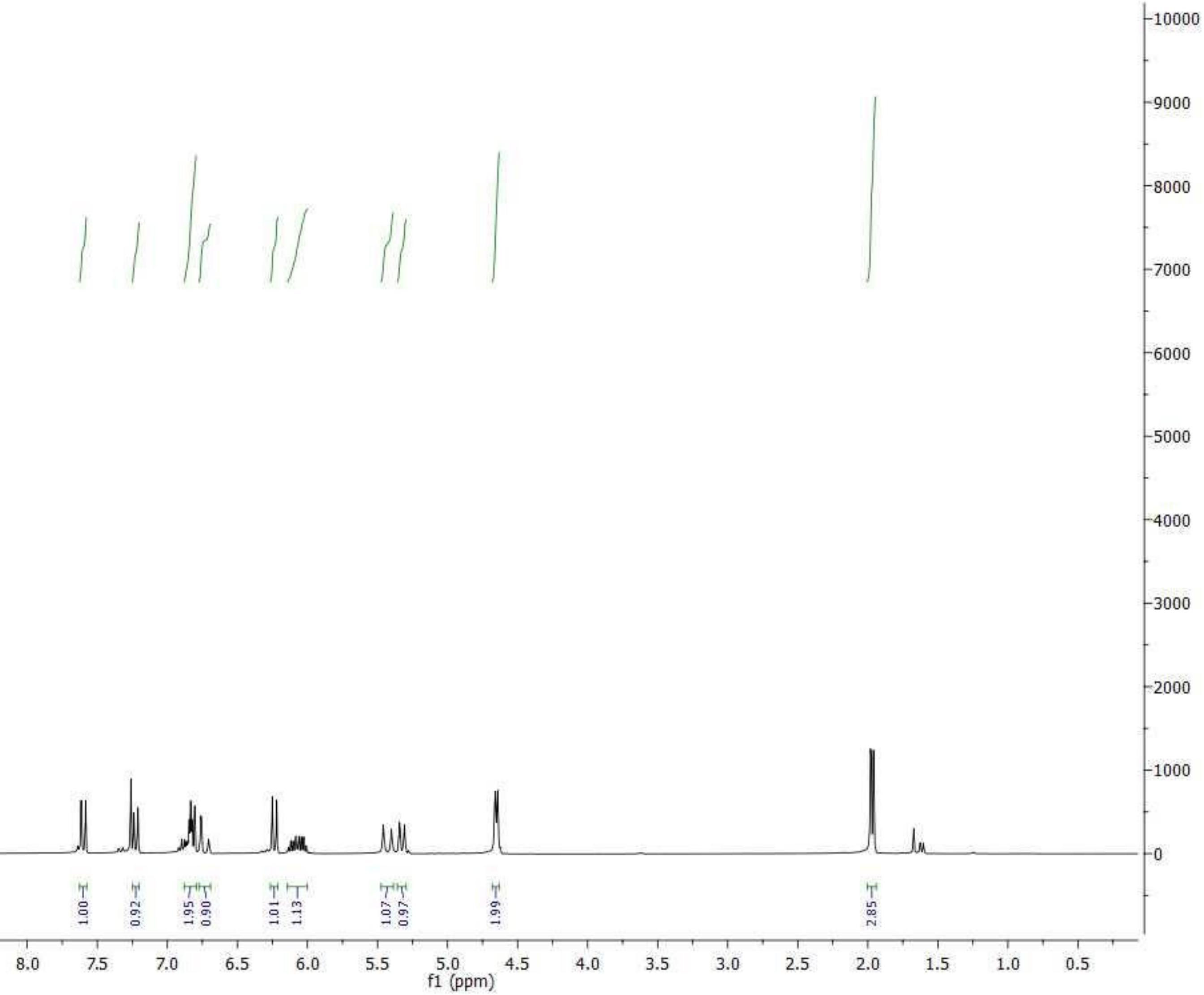

^{13}C NMR (75 MHz, CDCl$_3$) of **17a**

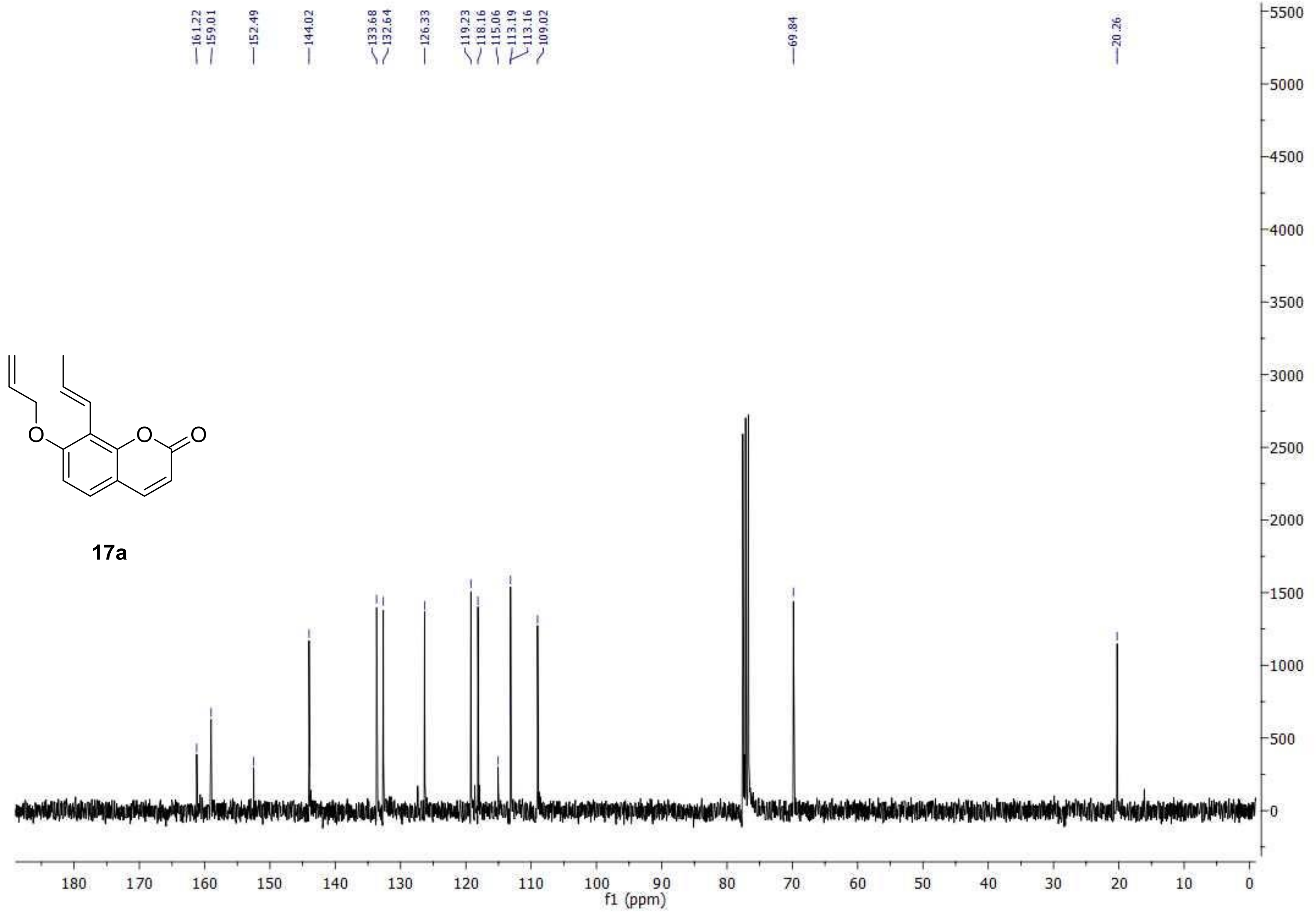

17a

^{1}H NMR (300 MHz, $CDCl_3$) of **17b**

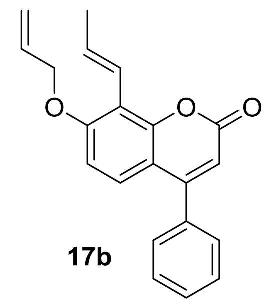

17b

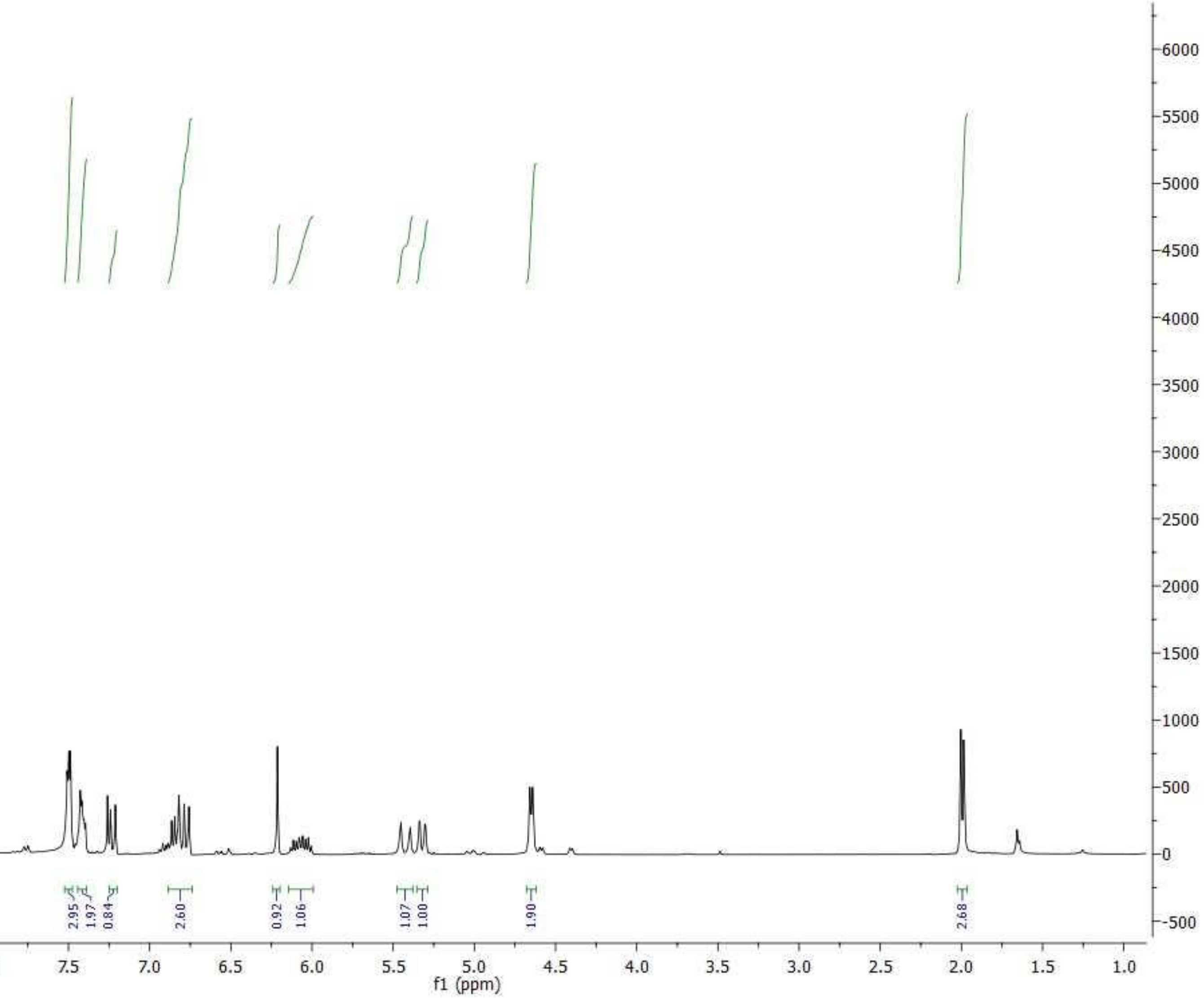

^{13}C NMR-APT (75 MHz, $CDCl_3$) of **17b**

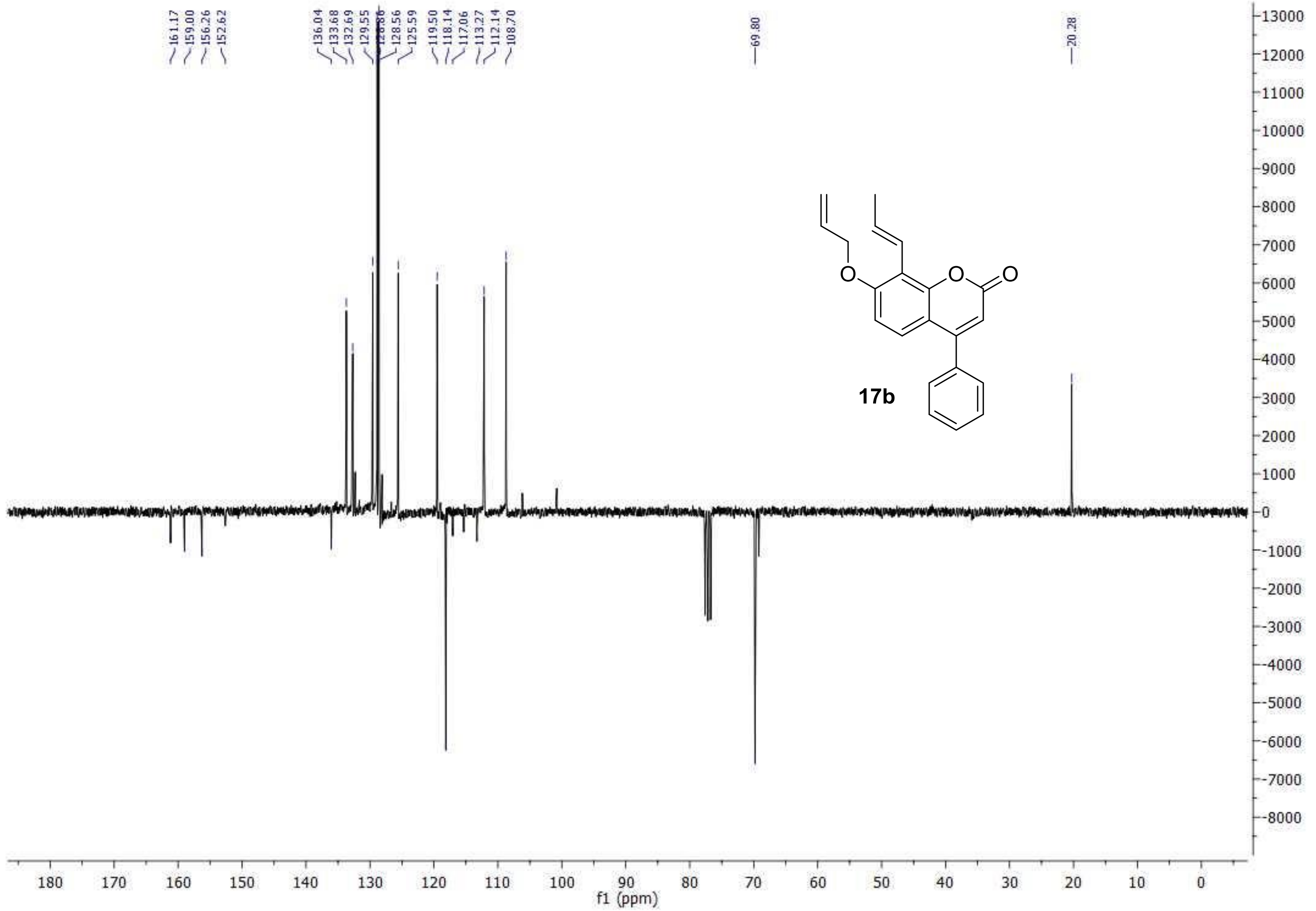

^{1}H NMR (300 MHz, $CDCl_3$) of **17c**

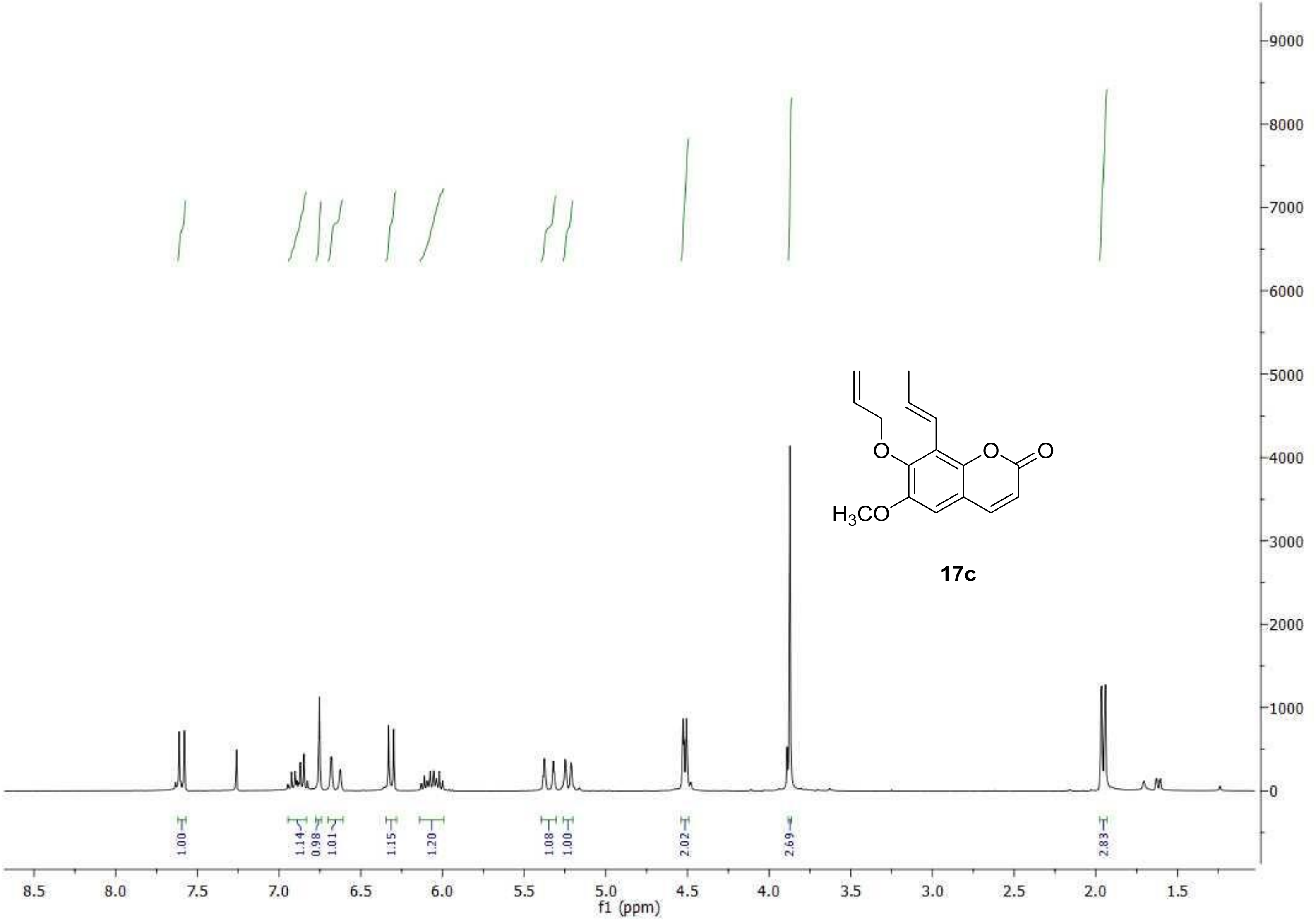

^{13}C NMR-APT (75 MHz, $CDCl_3$) of **17c**

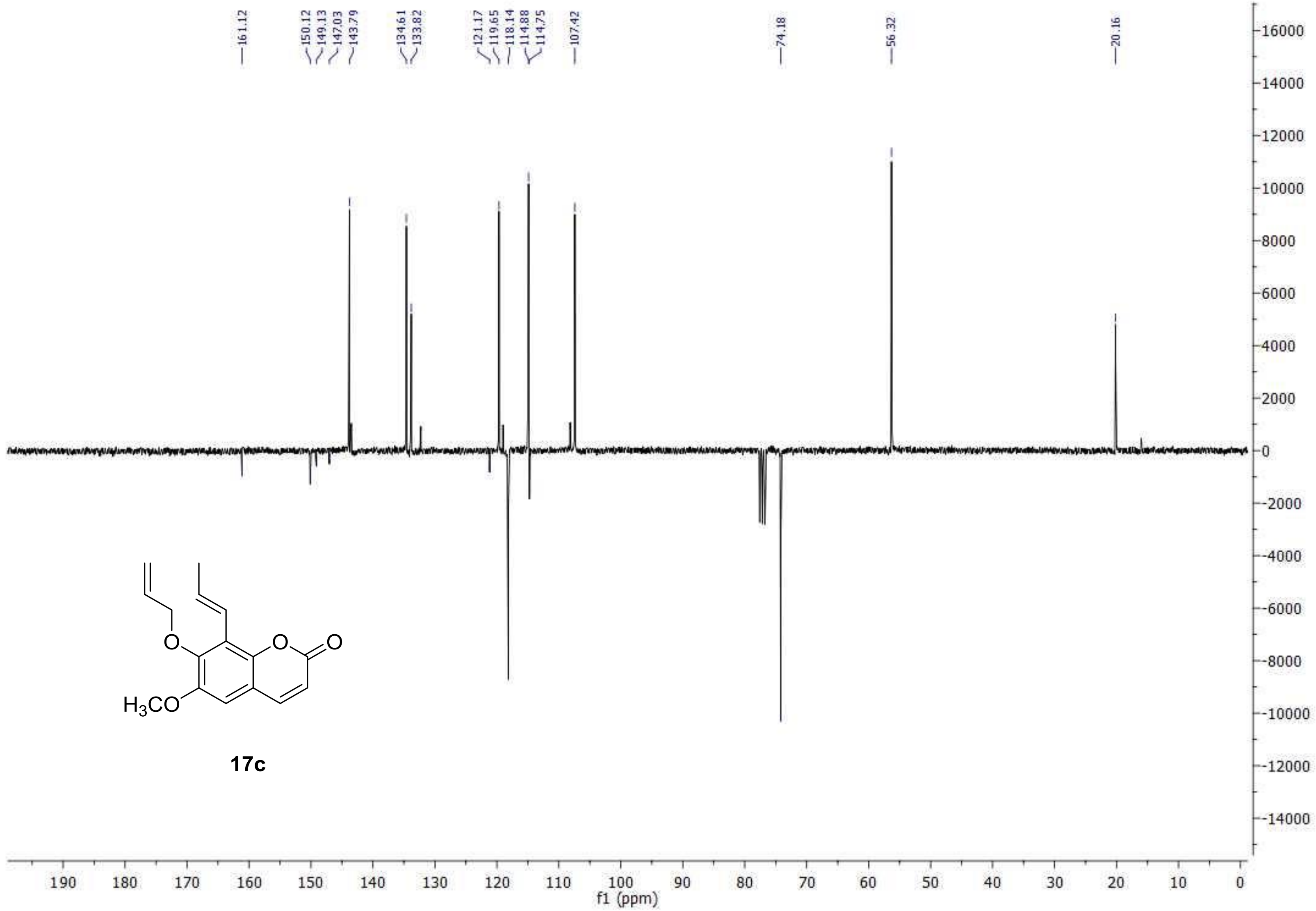

17c

^{1}H NMR (300 MHz, $CDCl_3$) of **17d**

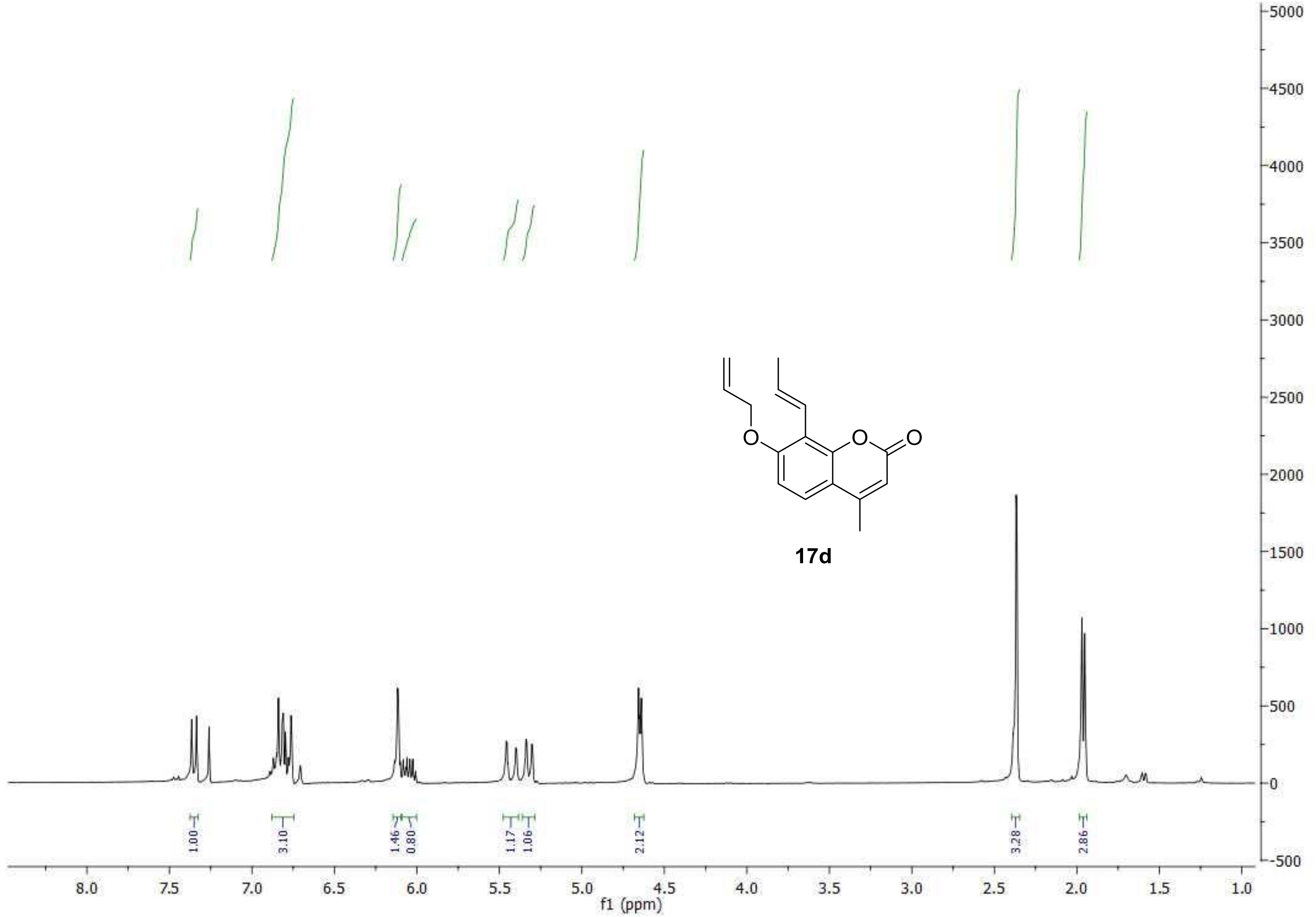

^{13}C NMR (75 MHz, $CDCl_3$) of **17d**

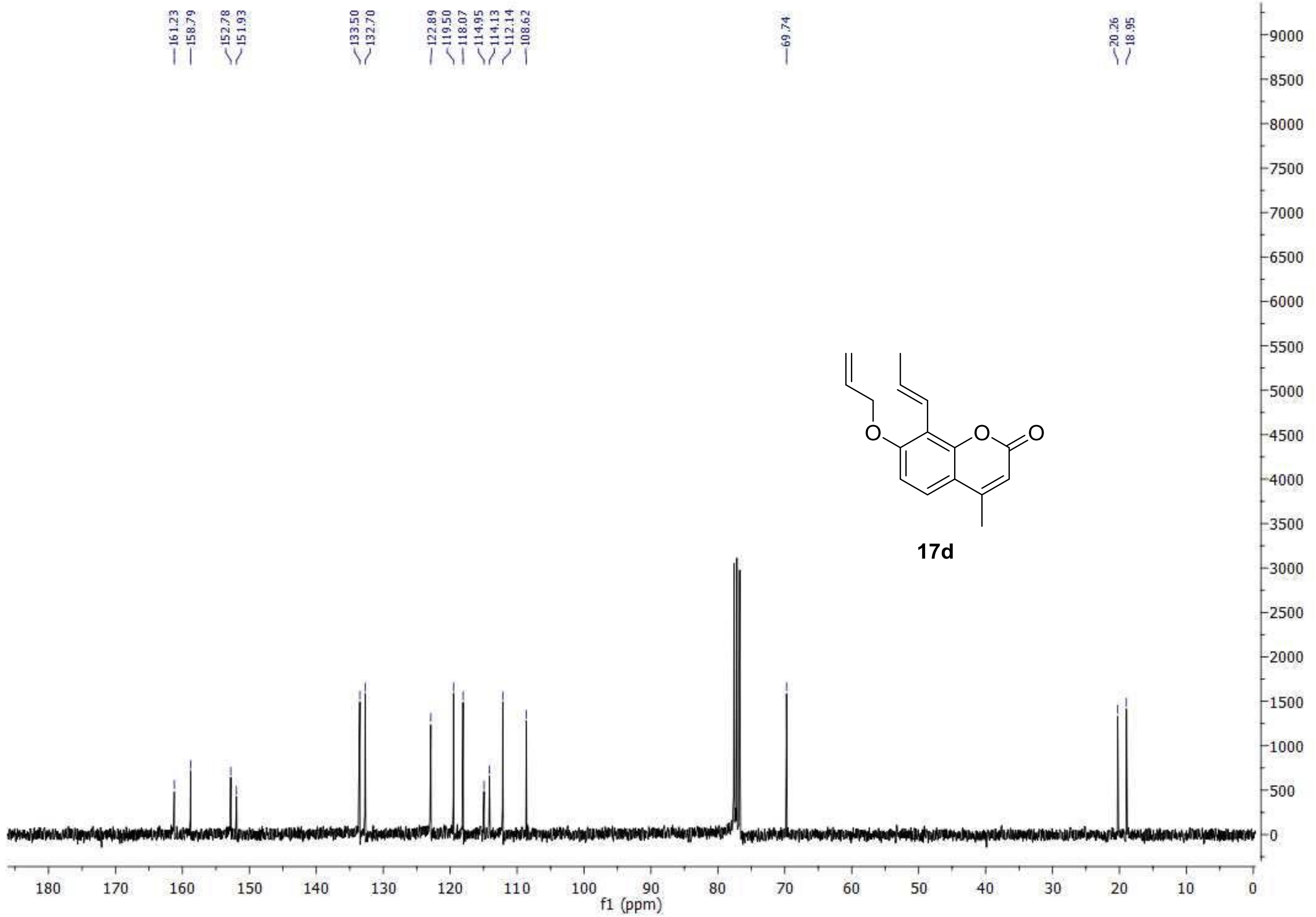

^{1}H NMR (300 MHz, DMSO-d_6) of **15a**

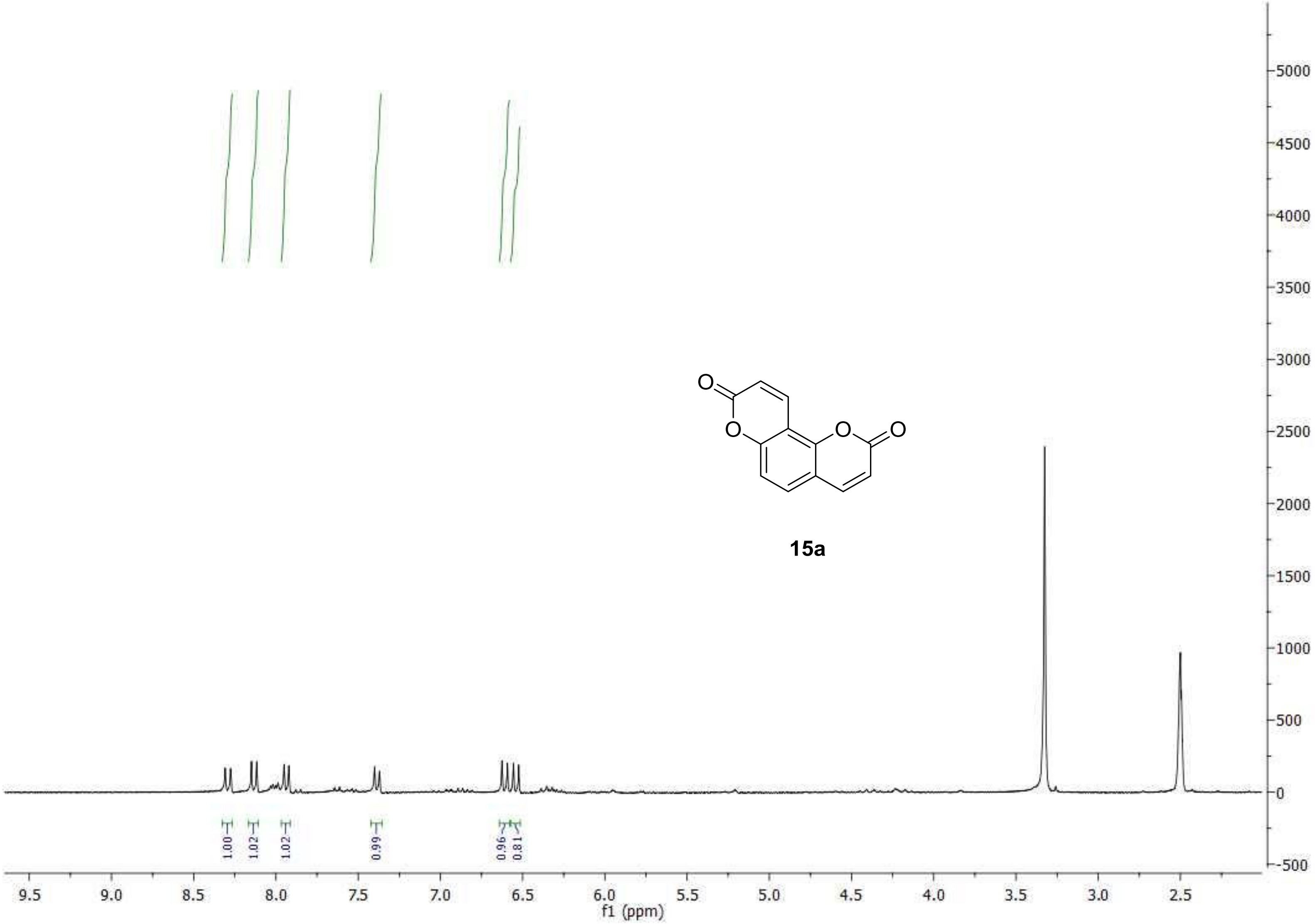

^{13}C NMR (75 MHz, DMSO-d_6) of **15a**

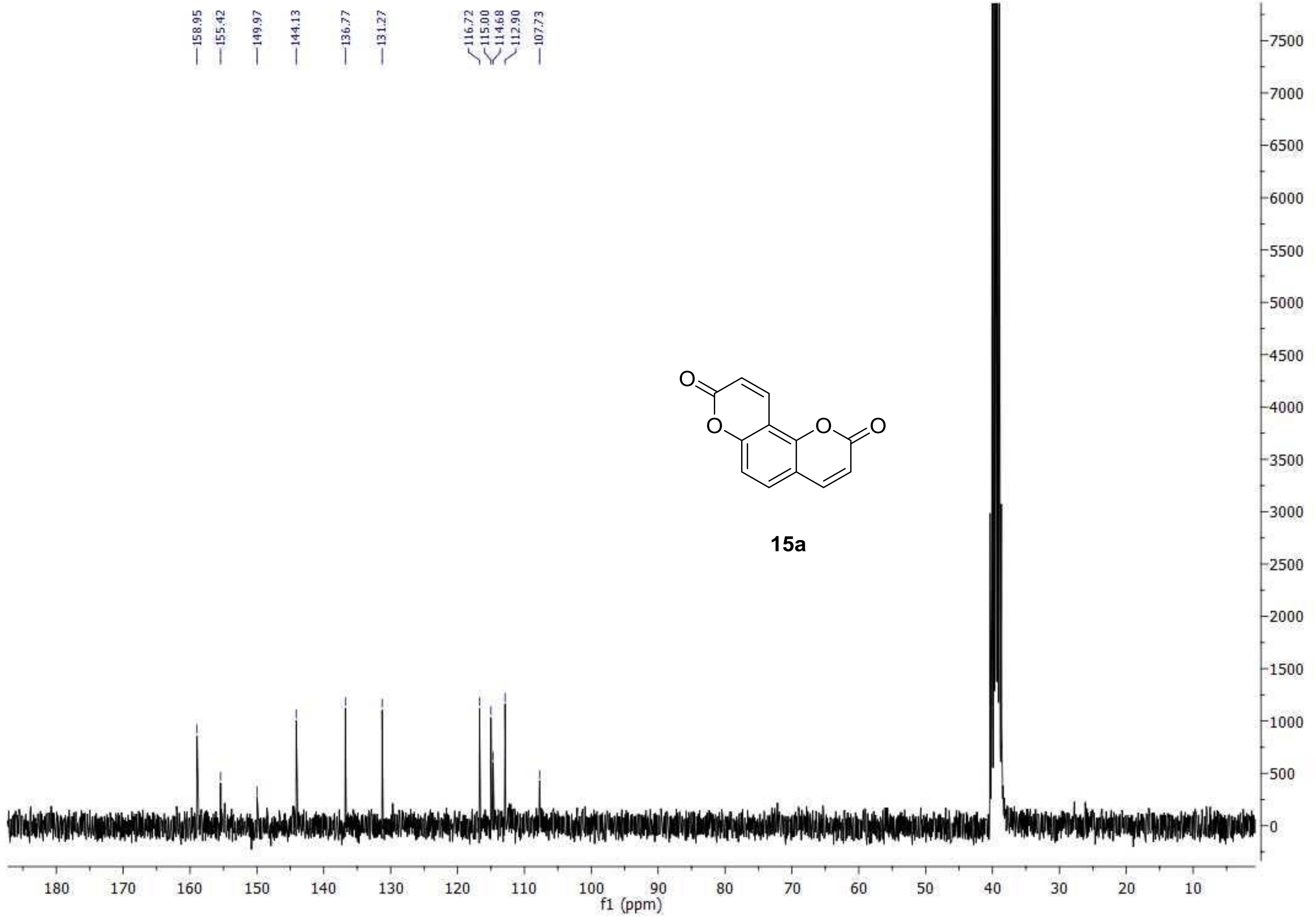

^{1}H NMR (300 MHz, $CDCl_3$) of **15b**

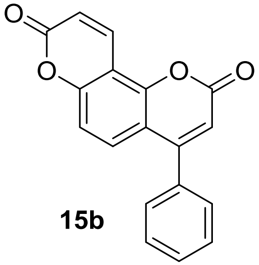

15b

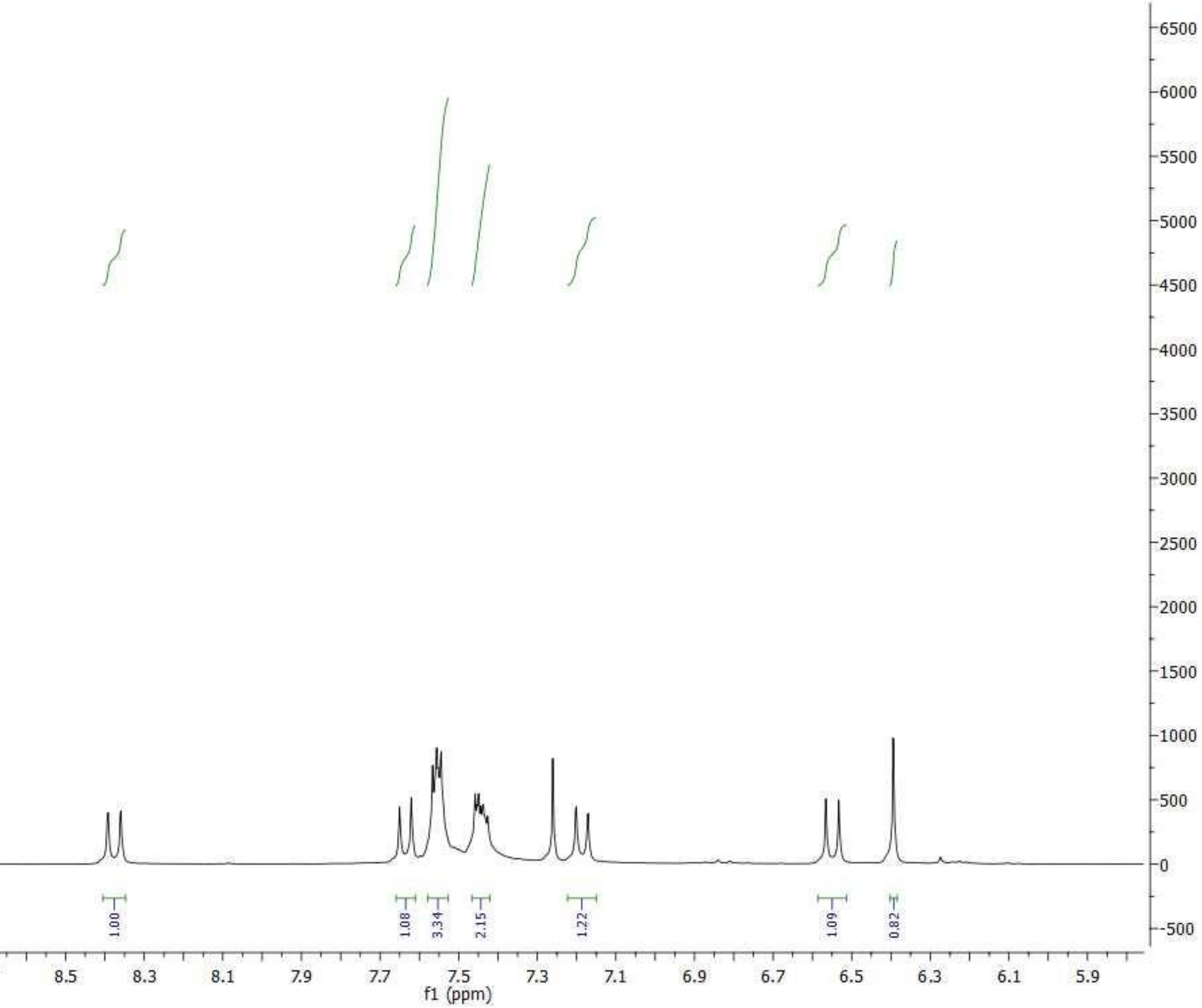

^{13}C NMR (75 MHz, $CDCl_3$) of **15b**

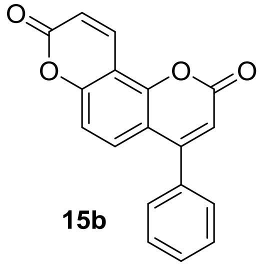

15b

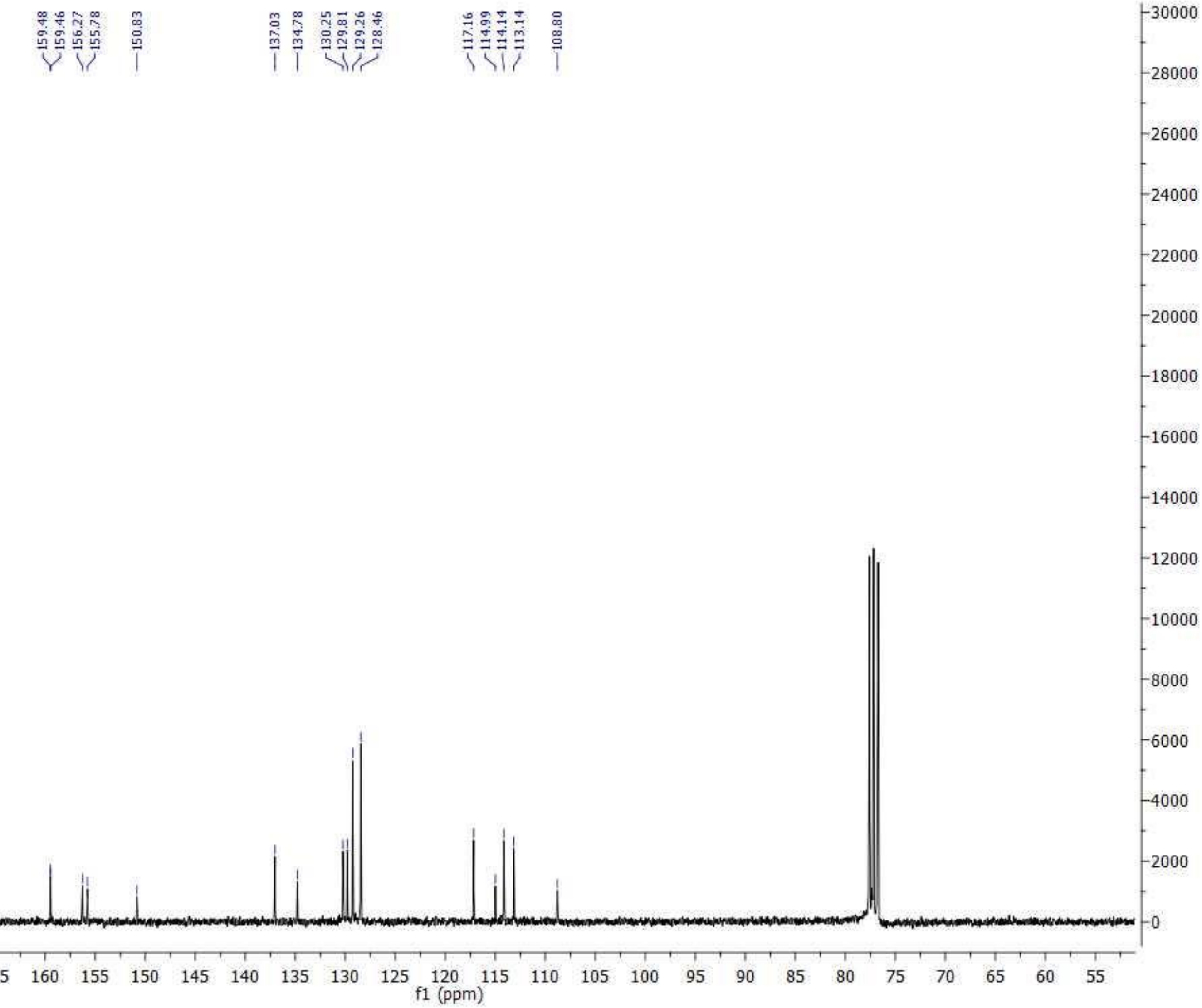

^{1}H NMR (300 MHz, $CDCl_3$) of **15c**

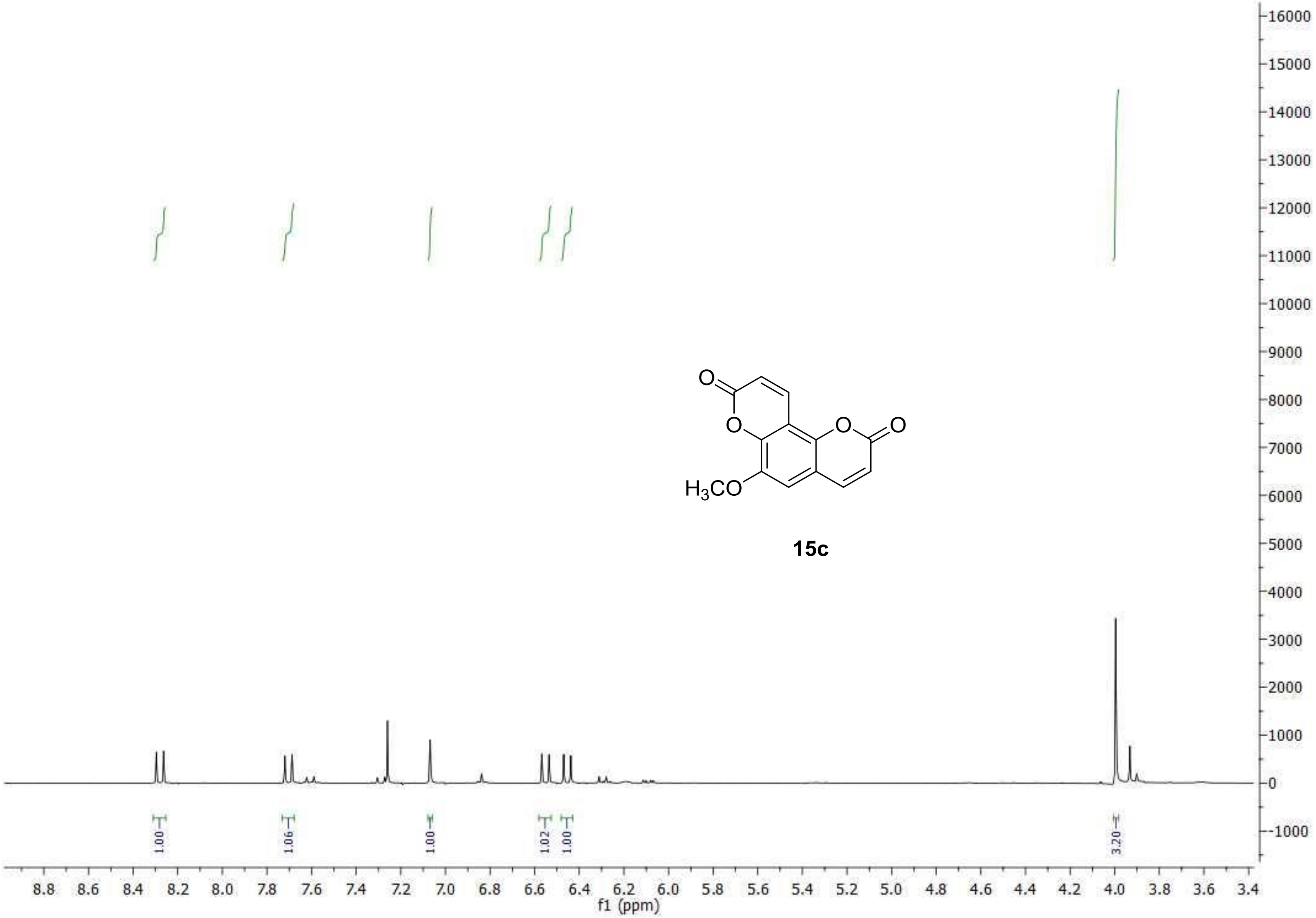

^{13}C NMR (75 MHz, $CDCl_3$) of **15c**

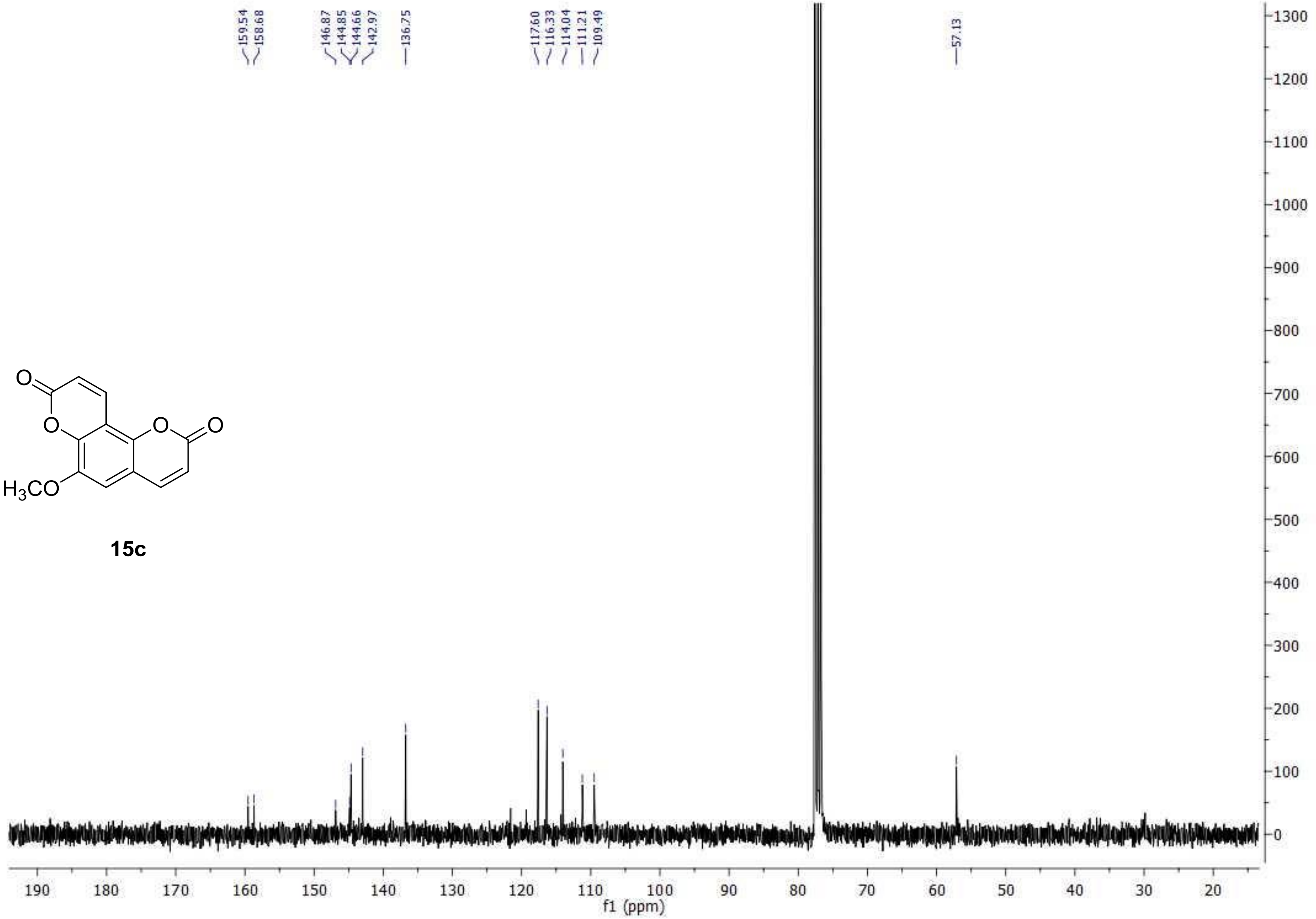

15c

^{1}H NMR (300 MHz, $CDCl_3$) of **15d**

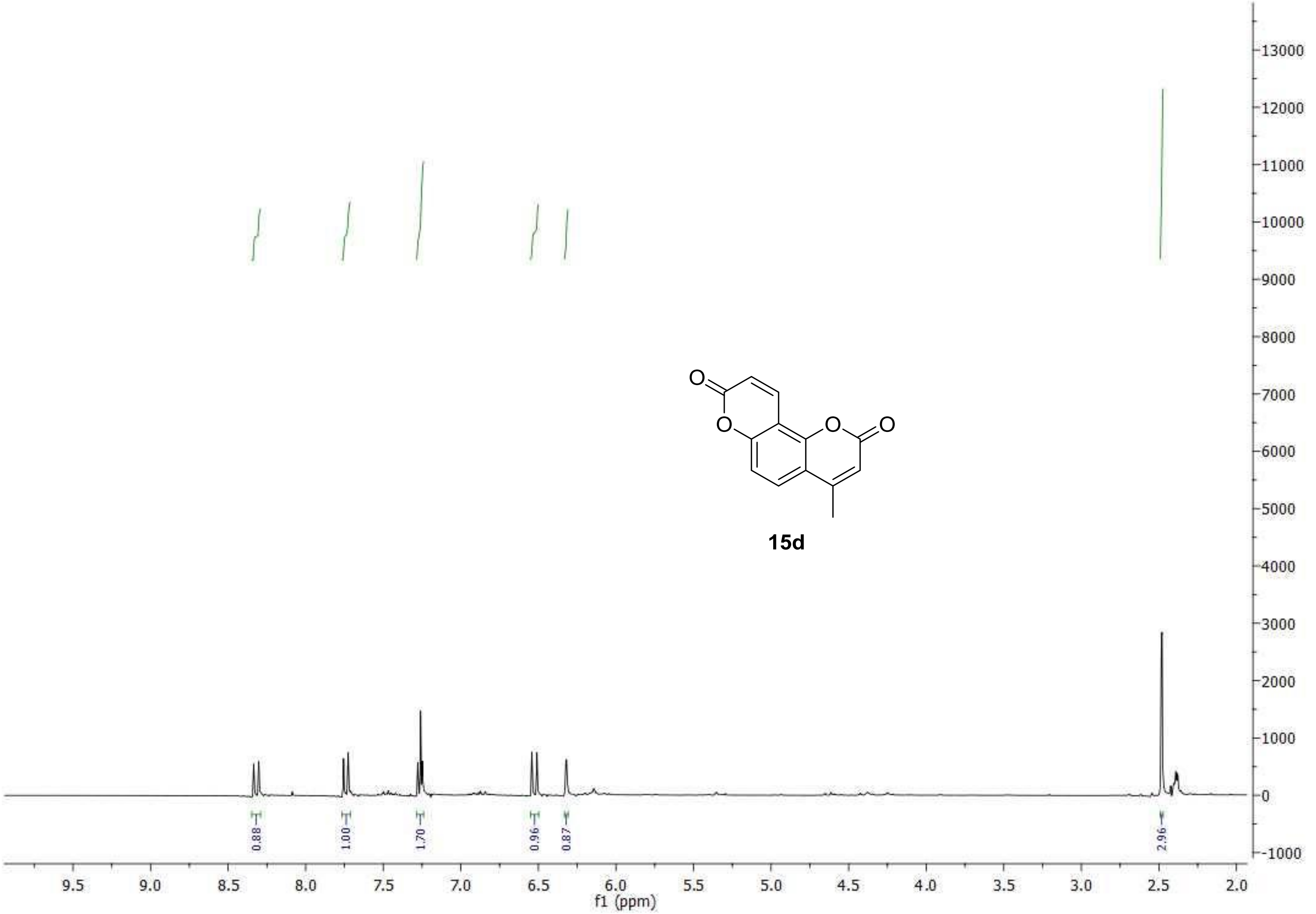

^{13}C NMR (75 MHz, $CDCl_3$) of **15d**

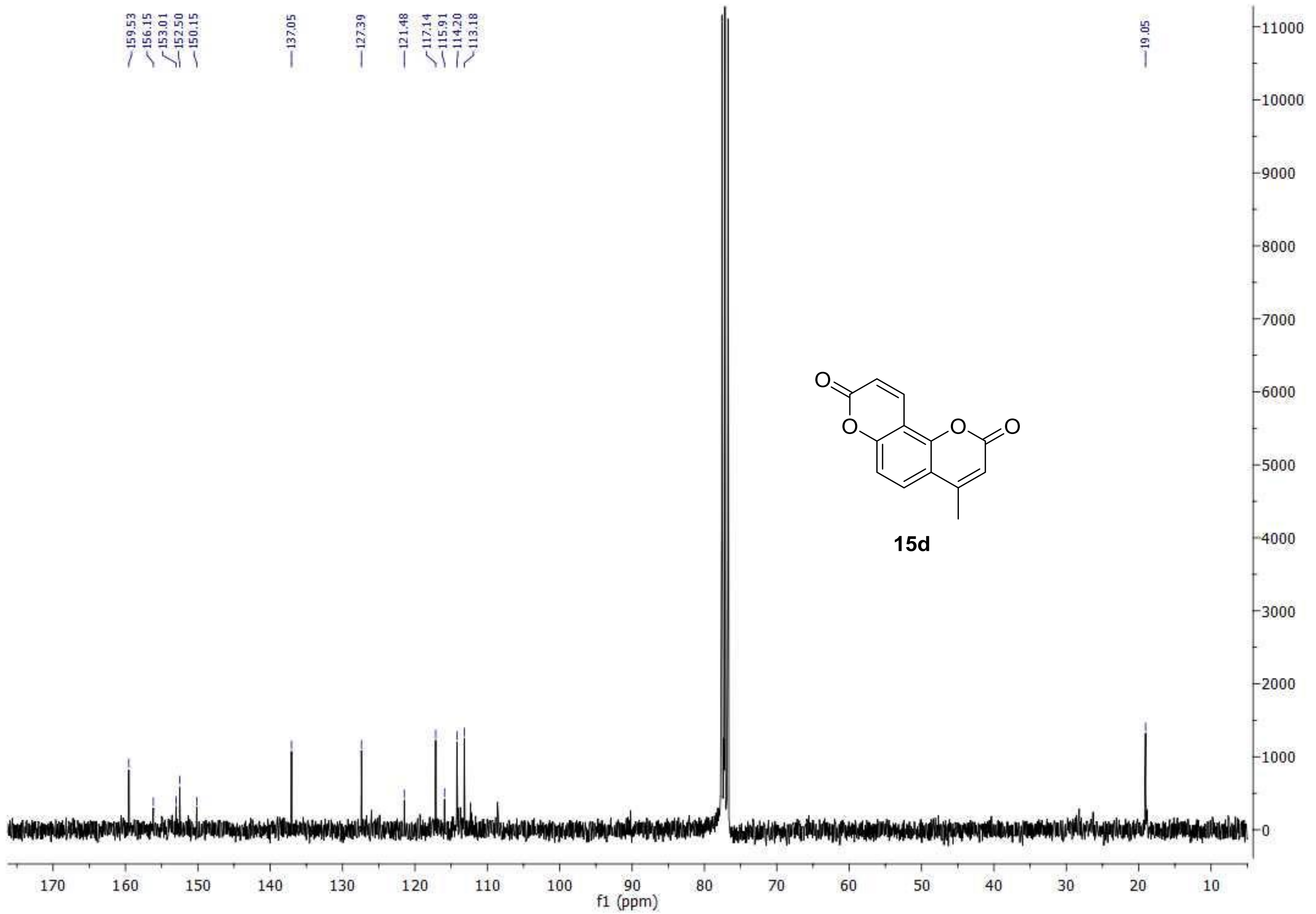

^{1}H NMR (300 MHz, DMSO-d_6) of **19**

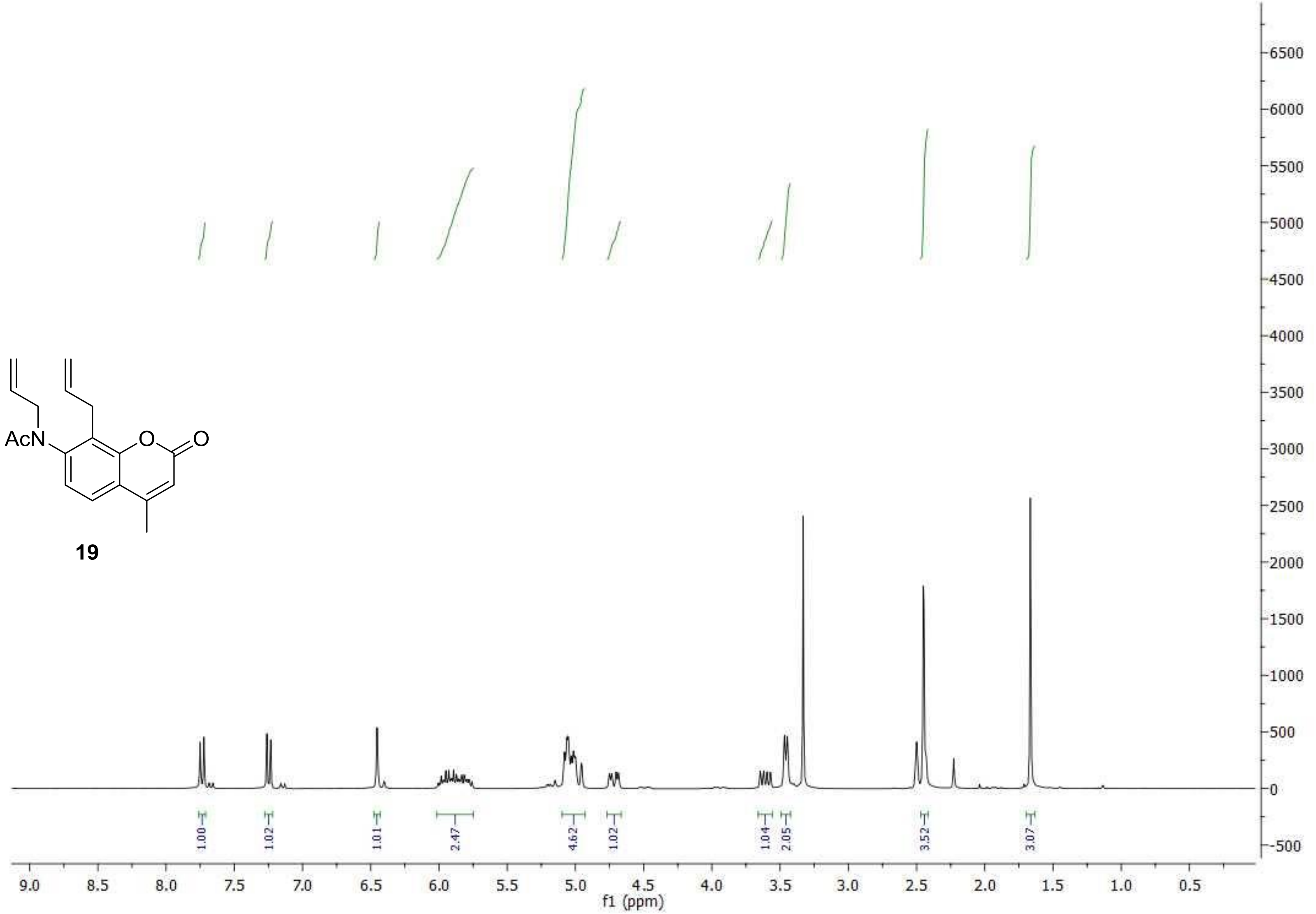

^{13}C NMR (75 MHz, DMSO-d_6) of **19**

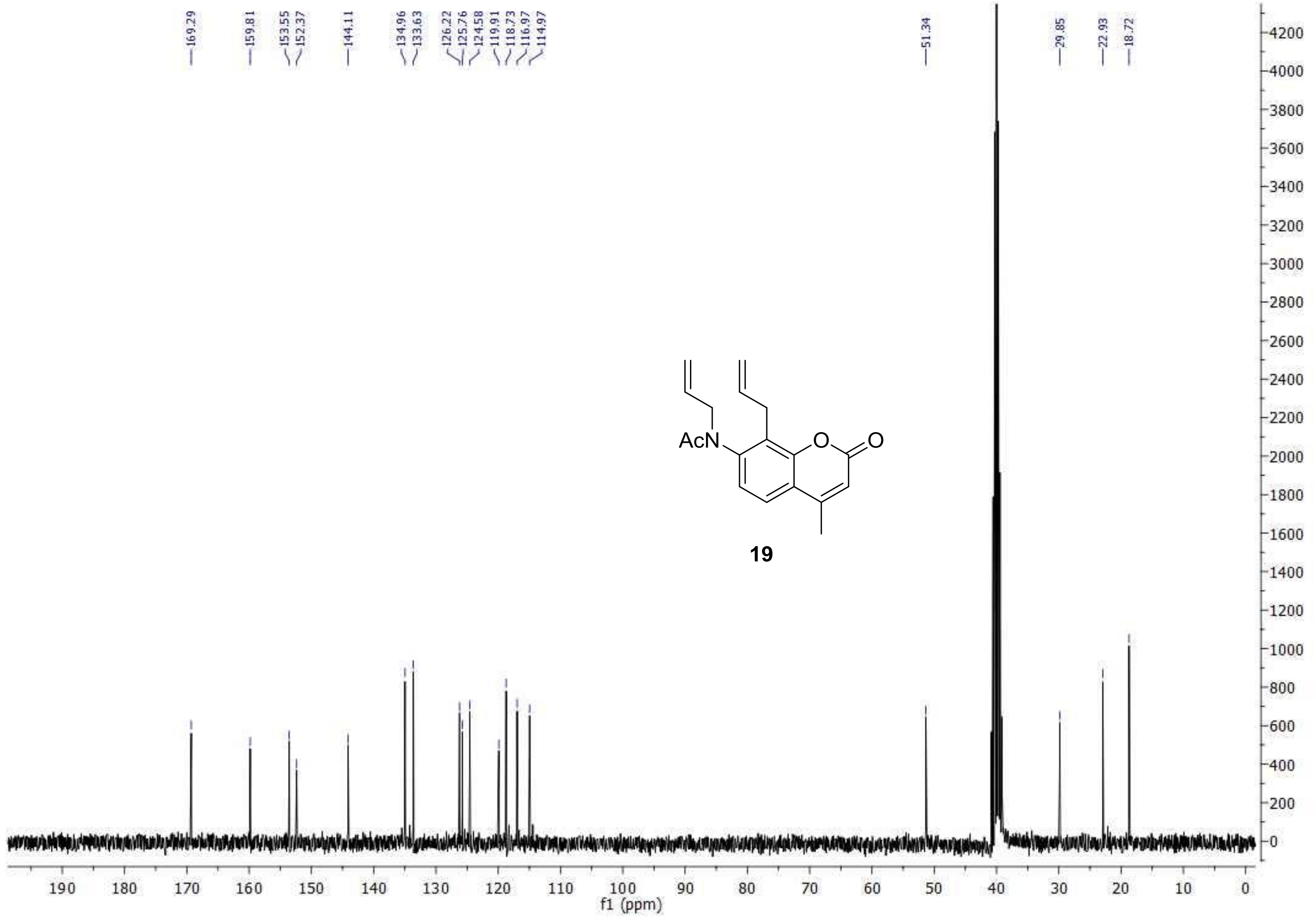

^{1}H NMR (300 MHz, $CDCl_3$) of **20**

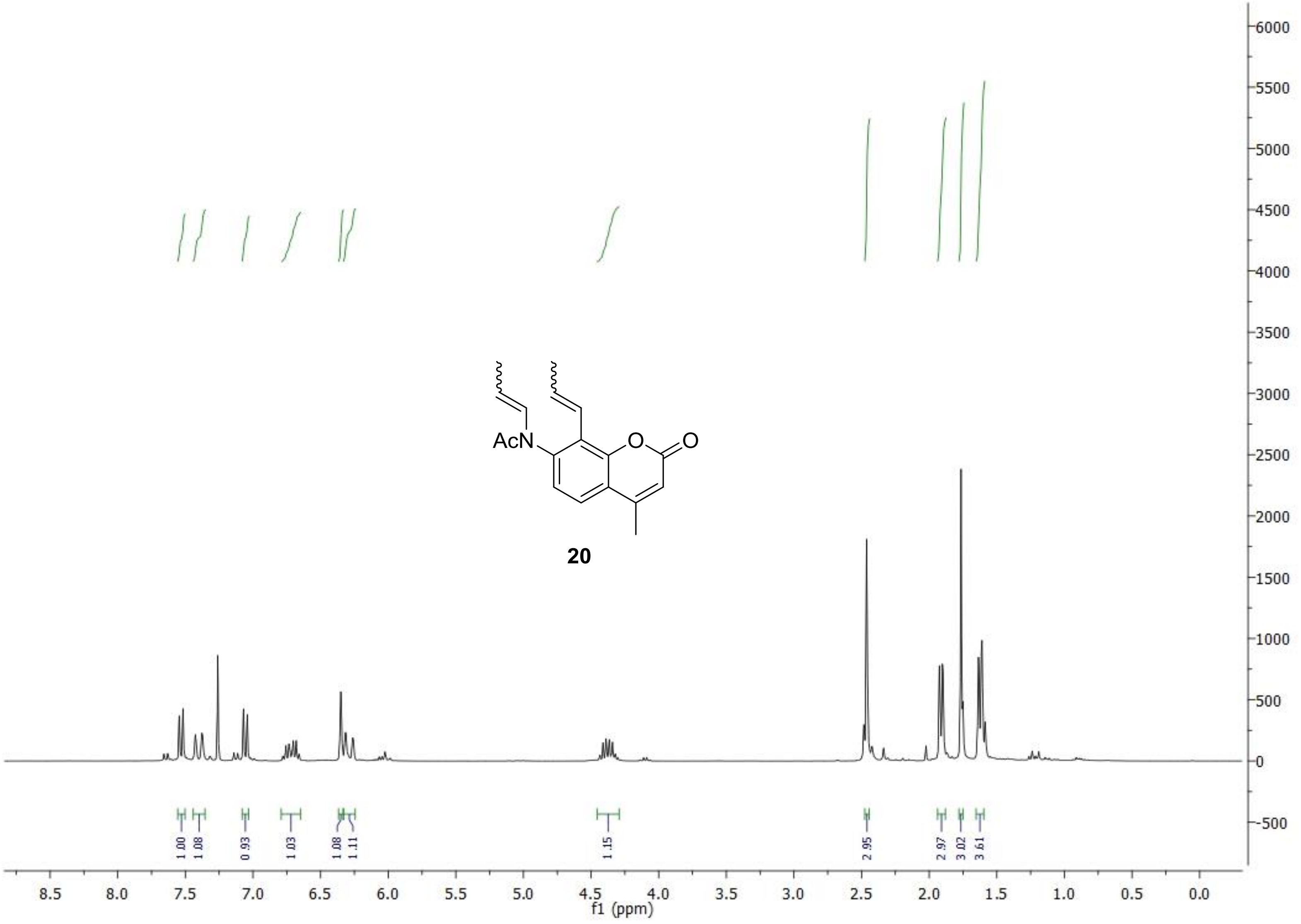

^{13}C NMR (75 MHz, $CDCl_3$) of **20**

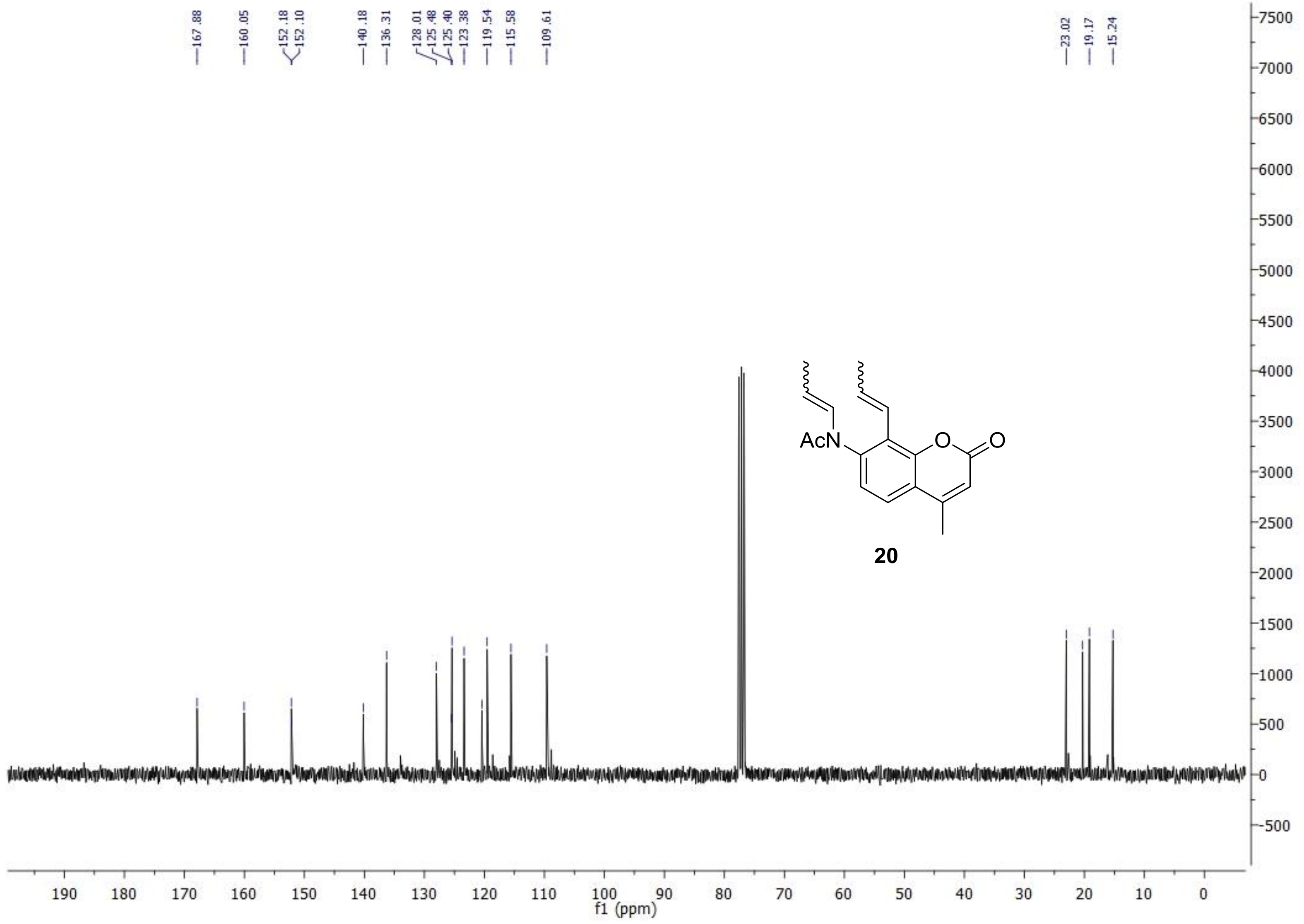

^{1}H NMR (300 MHz, $CDCl_3$) of **21**

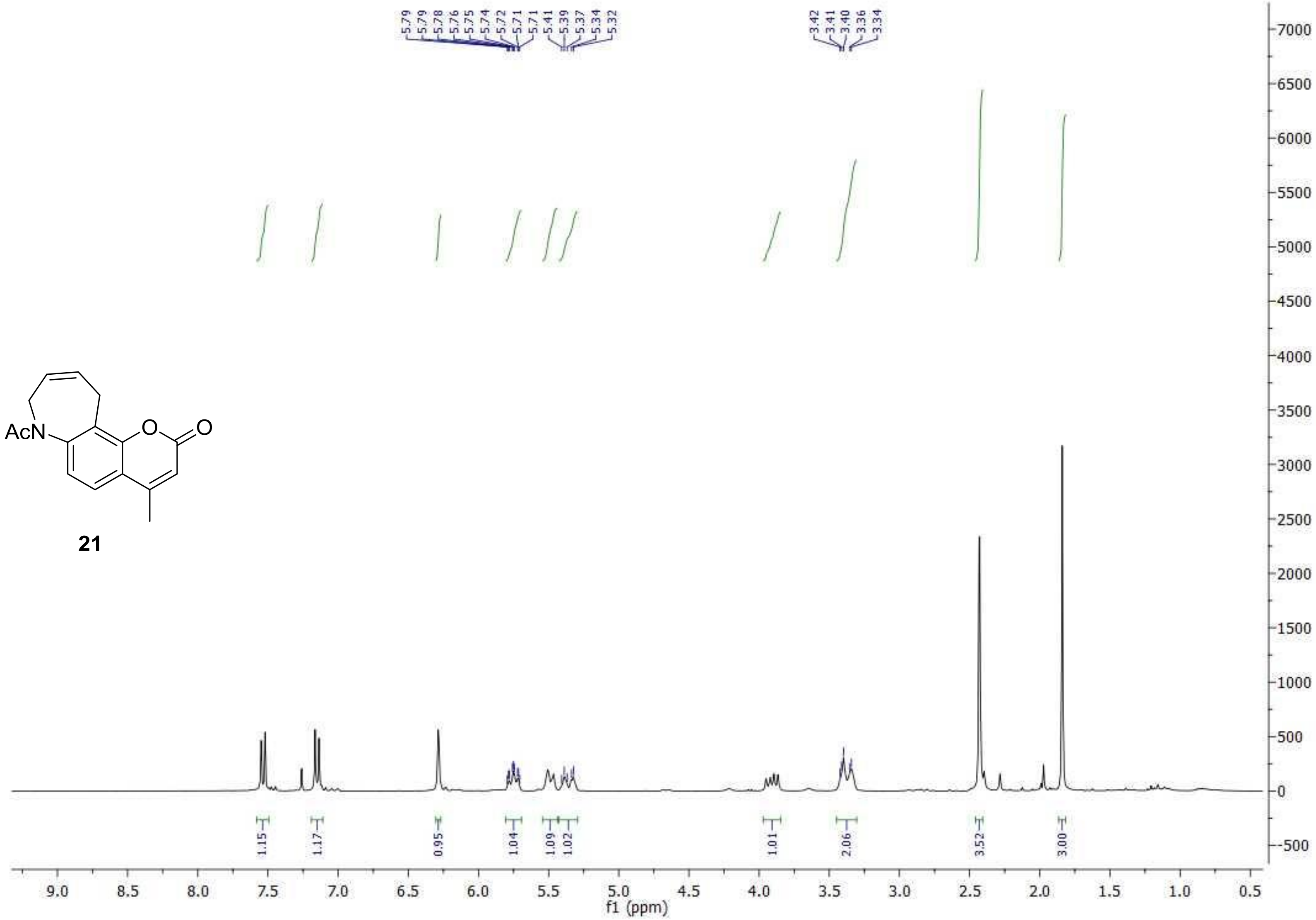

^{13}C NMR (75 MHz, $CDCl_3$) of **21**

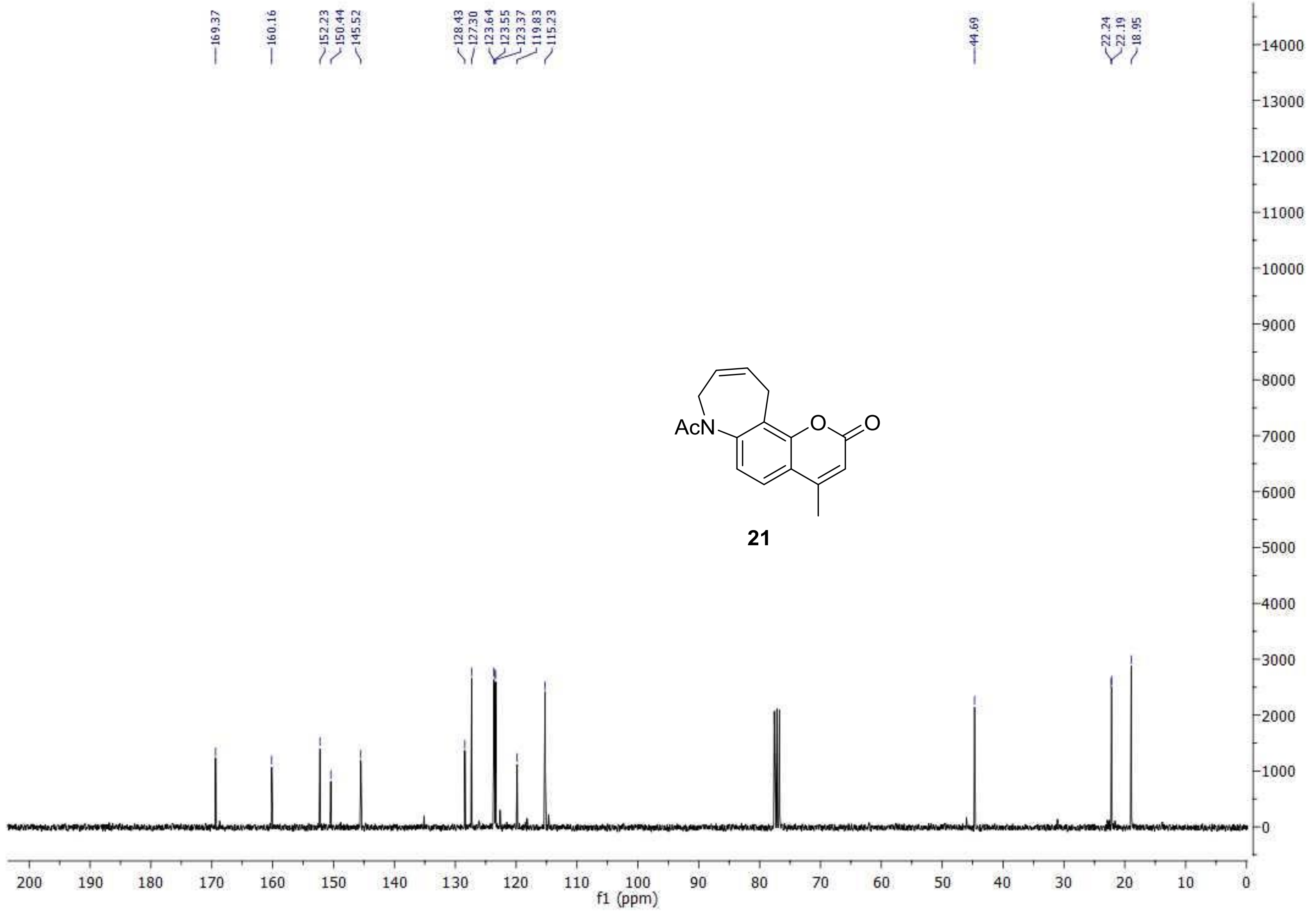

Supplement: File 1 — Full experimental procedures, characterization data and copies of 1H and 13C NMR spectra of all compounds. [file Beilstein_J_Org_Chem-14-2991-s001.pdf]
